# Identification of cross-stage, cross-species malaria CD8^+^ T cell antigens

**DOI:** 10.1038/s41586-026-10730-1

**Published:** 2026-07-01

**Authors:** Camila R. R. Barbosa, Luna B. de Lacerda, Paulo J. G. Bettencourt, David Morrow, Dhelio B. Pereira, Maya Aleshnick, Julie L. Mitchell, Nicholas C. Poulton, Cristopher Gomes, Lídia P. B. Cordeiro, Kadia Doumbia, Christina Ntalla, Charles Arama, Zezhou Zhao, Guilherme C. Maia, Gregório G. Almeida, Marie Rose Schrimpf, Thalia F. Hart, Derek Haumpy, Beatriz C. Medeiros-Rodrigues, Camila M. Costa, Annalisa Nicastri, Roxanne M. Gilbride, John B. Schell, Payton Kirtley, Lis R. V. Antonelli, Gaurav D. Gaiha, Scott G. Hansen, Judy Lieberman, Ricardo T. Gazzinelli, Moussa Niangaly, John Woodford, Joel Goldberg, Klaus Früh, Silvia Portugal, Patrick E. Duffy, Nicola Ternette, Brandon Wilder, Adrian V. S. Hill, Caroline Junqueira

**Affiliations:** 1 Instituto René Rachou, Fundação Oswaldo Cruz, Belo Horizonte, Brazil; 2 Centro de Tecnologia em Vacinas, Universidade Federal de Minas Gerais, Belo Horizonte, Brazil; 3 Jenner Institute, University of Oxford, Oxford, UK; 4 Center for Interdisciplinary Research in Health, Faculty of Medicine, Universidade Católica Portuguesa, Lisbon, Portugal; 5 Vaccine and Gene Therapy Institute, Oregon Health & Science University, Beaverton, OR USA; 6 Centro de Pesquisas em Medicina Tropical de Rondônia, Porto Velho, Brazil; 7 Laboratory of Malaria Immunology and Vaccinology, National Institute of Allergy and Infectious Diseases, National Institutes of Health, Bethesda, MD USA; 8 Departmaneto de Bioquímica e Imunologia, Instituto de Ciências Biológicas, Universidade Federal de Minas Gerais, Belo Horizonte, Brazil; 9 Institute for Research in Biomedicine, Università della Svizzera italiana, Bellinzona, Switzerland; 10 Parasites and Microbes Research and Training Center, University of Sciences, Techniques and Technologies of Bamako, Bamako, Mali; 11 Max Planck Institute for Infection Biology, Berlin, Germany; 12 Ragon Institute of Mass General, MIT and Harvard, Cambridge, MA USA; 13 Program in Cellular and Molecular Medicine, Boston Children’s Hospital, Boston, MA USA; 14 Department of Pediatrics, Harvard Medical School, Boston, MA USA; 15 Chester Beatty Laboratories, Institute of Cancer Research, London, UK; 16 Program in Health Sciences and Technology, Harvard Medical School and Massachusetts Institute of Technology, Cambridge, MA USA; 17 Division of Gastroenterology, Massachusetts General Hospital and Harvard Medical School, Boston, MA USA; 18 QIMR Berghofer, Brisbane, Queensland Australia; 19 School of Life Sciences, University of Dundee, Dundee, UK

**Keywords:** Malaria, Protein vaccines, Proteomics

## Abstract

A major limitation on the development of a malaria vaccine is the lack of validated T cell epitope targets. *Plasmodium falciparum* is the most prevalent malaria parasite affecting humans in Africa, whereas *Plasmodium vivax* is more widespread and is the main species that causes malaria in the Americas and Asia^1^. *P. vivax* exclusively infects peripheral-blood reticulocytes, which retain RNA and the capacity for host protein synthesis^2^. We previously reported that reticulocytes infected with *P. vivax* express human leukocyte antigen class I (HLA-I), which enables recognition and killing of the parasite by CD8^+^ T cells^3^. Here we use immunopeptidomics to identify *Plasmodium-*antigen-derived peptides presented by HLA-I on infected reticulocytes. We identified 453 unique peptides, mapping to 166 proteins. Seventy-five antigens were housekeeping proteins that are constitutively expressed at multiple stages of the parasite’s life cycle and are highly conserved between *Plasmodium* species. Identical peptides were presented in different individuals by the same or distinct HLA-A, HLA-B and HLA-C alleles, as well as by the non-classical HLA-E allele. The antigenicity of the newly identified epitopes was validated in samples from both *P. vivax*-infected and *P. falciparum*-infected individuals. Furthermore, T cell responses to several of these antigens were observed in the blood and liver of non-human primates after infection with *Plasmodium* or immunization with attenuated parasites. Two antigens also induced protective CD8^+^ T cell-mediated immunity in rodents. Thus, these antigens have the potential for use in a cross-stage and cross-species malaria vaccine.

## Abstract (web summary)

An immunopeptidomics analysis identifies numerous *Plasmodium* T cell antigens that are conserved across malaria parasite species and expressed at several phases of the parasite’s life cycle, with possible applications in the development of a malaria vaccine.

## Main

A promising strategy for controlling malaria is the development of a vaccine that blocks transmission and persistent infection. Most vaccine efforts focus on eliciting antibodies against *Plasmodium* proteins that prevent the parasite invading hepatocytes or red blood cells (RBCs)^4,5^. Immunization with live attenuated parasites, which elicit both neutralizing antibodies and T cell responses that kill liver-stage parasites, offers the highest protection^5,6^. However, little is known about which parasite antigens T lymphocytes recognize, which makes the development of protective T cell vaccines a major challenge.

Most malaria research has centred on *P. falciparum* (*Pf*), leaving *P. vivax* (*Pv*) relatively neglected. The World Health Organization currently recommends two *Pf*-specific vaccines: RTS,S/AS01 and R21/Matrix-M^7^. Both of these are based on the *Pf* circumsporozoite protein (CSP) and induce neutralizing antibodies. These vaccines target only the *Pf* pre-erythrocytic stage and prevent clinical malaria in children; RTS,S confers around 30% protection^8^ and R21 offers greater than 70% protection^9^. However, efficacy wanes over time, and booster doses are required. Vaccines that use attenuated live sporozoites are the only ones that provide sterilizing immunity and also induce strong CD8^+^ and γδ T cell responses^5,6^. Progress on *Pv* vaccines has been slow, focusing mainly on *Pf*-orthologous antigens or on proteins that mediate the binding of *Pv* to reticulocytes, such as Duffy-binding protein (DBP) and merozoite surface protein 1 (MSP1)^10^. An ideal vaccine would provide multi-year protection against both liver and blood stages and target antigens that are shared across species.

T lymphocytes were originally thought to recognize only the *Plasmodium* liver stage, because mature RBCs lack HLA-mediated antigen presentation. But whereas *Pf* mainly infects mature RBCs, *Pv* infects immature RBCs: reticulocytes^2^. Our previous work showed that CD8^+^ T cells can recognize *Pv*-infected reticulocytes, lyse these cells and kill the intracellular parasite^3^. Here we build on this discovery by using immunopeptidomics to identify *Pv* peptides presented by HLA-I on infected reticulocytes, uncovering an array of putative T cell malaria epitopes. Notably, 75% of the 166 identified antigens are expressed in *Pv* sporozoites, liver and blood stages, and 71% have orthologues across *Plasmodium* species. Cross-species recognition was confirmed for all 50 peptides tested in at least 2 *Plasmodium* species. These epitopes were antigenic in *Pv*- and *Pf*-infected people, *Plasmodium cynomolgi* (*Pcy*)- and *Plasmodium knowlesi* (*Pk*)-infected non-human primates (NHPs) and *Plasmodium yoelii* (*Py*)-infected mice. On the basis of these data, we hypothesize that these broadly recognized *Plasmodium* T cell antigens could be used to design a universal cross-species malaria vaccine that induces T cell-mediated immunity against liver- and blood-stage parasites.

## Profiling of HLA-I-bound *Pv* peptides

**Fig. 1:**
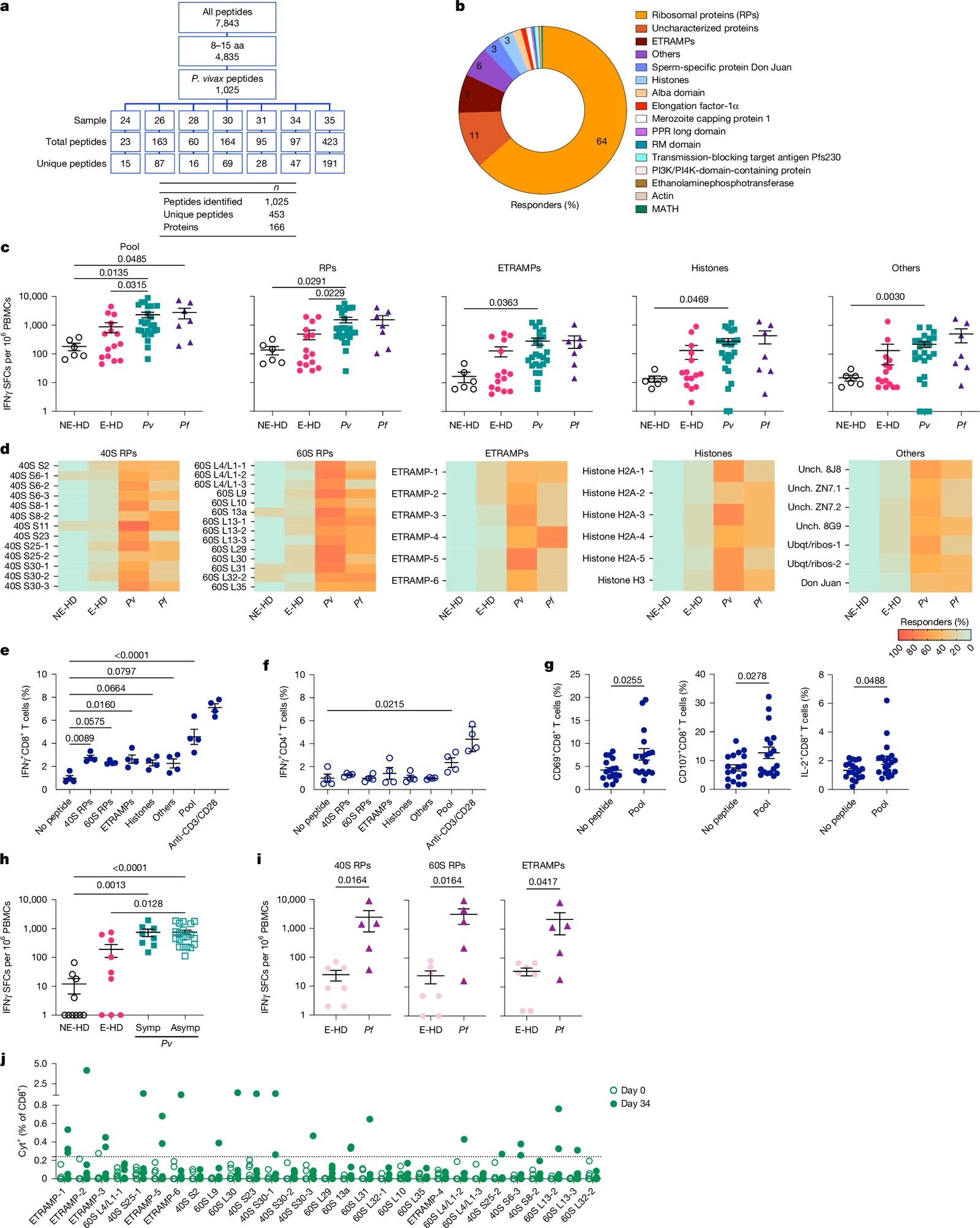
Identification and antigenic validation of *Pv* HLA-I-associated peptides. **a**, Peptides were identified by liquid chromatography–tandem mass spectrometry (LC–MS/MS) using human (UniProt UP000005640, 09-2019) and *Pv*Sal1 (UniProt UP000008333, 08-2019) reference proteomes. Samples (24, 26, 28, 30, 31, 34 and 35) represent anonymized donors. Unique peptides that mapped to the *Pv*Sal1 proteome, were 8–15 amino acids (aa) long and were absent from the human proteome were selected for analysis. **b**, Frequencies of *Pv*-derived peptides by protein family. **c**,**d**, Anti-IFNγ ELISpot responses to selected peptides using PBMCs from *Pv*-infected individuals (*n* = 24), *Pf*-infected individuals (*n* = 7), E-HD individuals (*n* = 15) and NE-HD individuals (*n* = 6). **c**, PBMCs were stimulated with a 50-peptide pool or protein family pools (RPs, ETRAMPs, histones and others). Results are shown as SFCs per 10^6^ PBMCs. **d**, Heat maps showing the percentages of responders per peptide and cohort (green, low; light orange, intermediate; dark orange, high). Ubqt/ribos, ubiquitin–ribosomal; Unch, uncharacterized. **e**,**f**, PBMCs from symptomatic patients with *Pv* infection (*n* = 4) were stimulated with peptide pools; IFNγ^+^CD8^+^ T cells (**e**) and IFNγ^+^CD4^+^ T cells (**f**) were quantified by ICS. Anti-CD3/anti-CD28 (anti-CD3/CD8) were used as positive controls, and no peptide as a negative control. **g**, PBMCs from symptomatic patients with *Pv* infection (*n* = 18) were stimulated with the full peptide pool; CD8^+^ T cell activation (CD107a, CD69) and IL-2 were measured by flow cytometry.** h**, IFNγ ELISpot responses to the total peptide pool from E-HD (*n* = 10), NE-HD (*n* = 10), *Pv* symptomatic (Symp; *n* = 8), and *Pv* asymptomatic (Asymp; *n* = 20) individuals. **i**, IFNγ ELISpot responses to selected peptides using PBMCs from Malian *Pf*-infected individuals (*n* = 5) and Malian E-HD individuals (*n* = 7). **j**, Antigen-specific CD8^+^ T cell responses in CHMI volunteers (*n* = 6) before challenge (day 0) and on day 34 after *Pv* blood-stage challenge; responses reflect IFNγ and/or TNF (Cyt^+^) after subtraction of dimethyl sulfoxide (DMSO) controls. Statistical analyses: Kruskal–Wallis test (**c**,**e**,**f**,**h**), and Mann–Whitney test (**g**,**i**). Mean ± s.e.m. and exact *P* values are shown.

**Extended Data Fig. 1:**
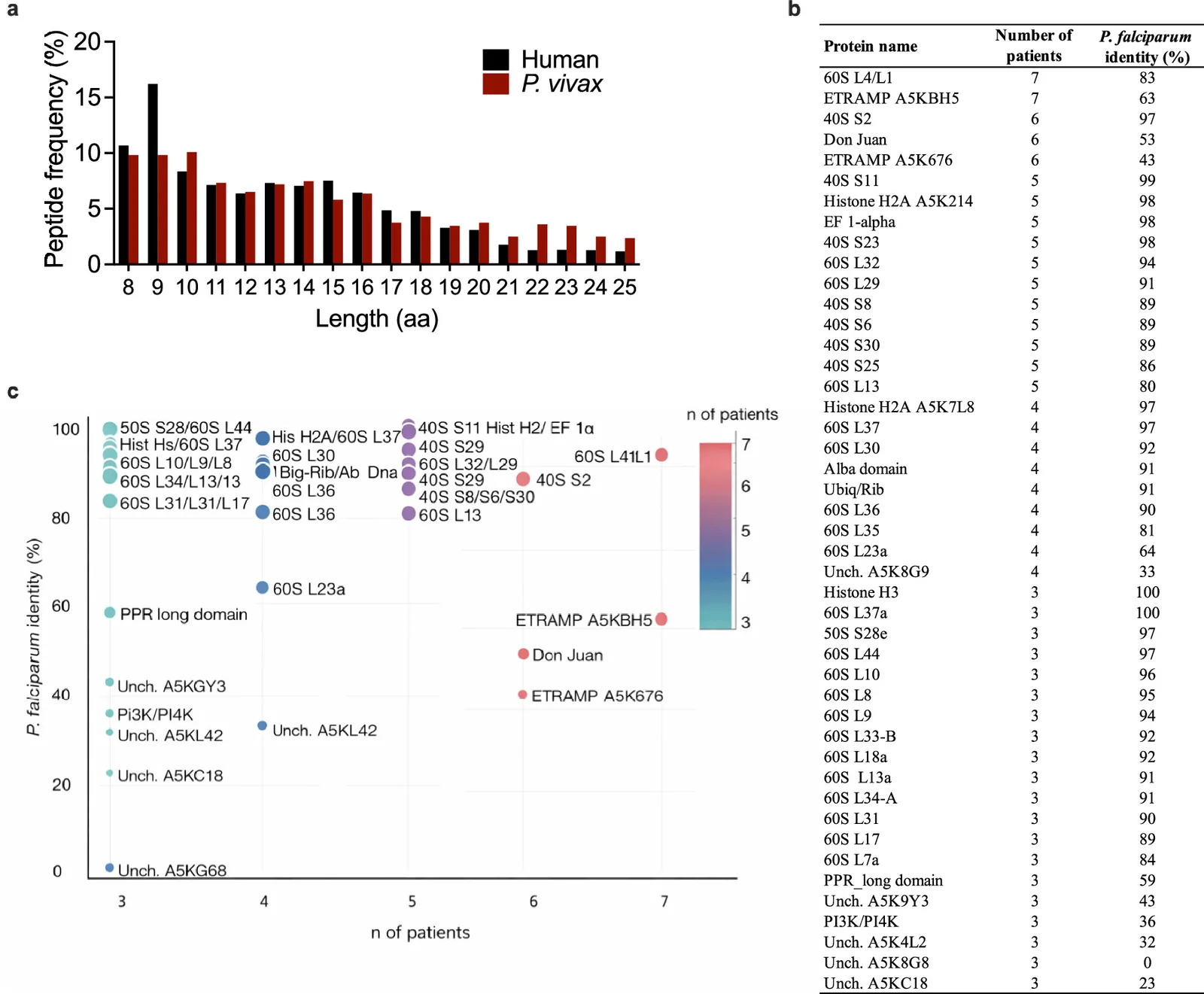
Comparative analysis of the length distribution of *Pv* and human peptides and protein identity between *Pv* and *Pf.* **a**, Distribution of peptide lengths (8–25 amino acids) for human and *Pv* peptides. **b**, Table of *Pv* proteins with the number of patients in which each protein was detected, and the percentage of sequence identity to *Pf* orthologs. **c**, Each point represents a parasite-peptide. The *y *axis indicates protein sequence identity to *Pf*, and the *x* axis shows the number of patients in which the peptide was detected. Colours denote patient frequency: pink (7 patients), orange (6), purple (5), blue (4), and green (3).

To identify *Pv* peptides presented by HLA-I on infected reticulocytes, we obtained peripheral-blood samples from seven individuals infected with acute *Pv.* The individuals, who were recruited in Brazil, had diverse HLA backgrounds. Samples were anonymized as 24, 26, 28, 30, 31, 34 and 35 (Supplementary Information 1). Highly purified samples of reticulocytes contained 4.3 × 10^7^ to 7 × 10^8^ cells, comprising 98.1 ± 1.2% infected reticulocytes, with minimal uninfected RBCs and negligible contamination from nucleated cells. Using an optimized workflow for HLA-I peptide purification and mass spectrometry^11^, we identified 7,843 peptides, of which 4,835 had a size distribution consistent with HLA-I peptide (8–15 amino acids)^12^. A total of 1,025 peptides mapped to the *Pv* proteome (proteome ID: UP000305196), with per-sample yields ranging from 23 to 423 peptides (Fig. 1a). Combining all samples, we identified 453 unique *Pv* peptides from 166 proteins (Fig. 1a and Supplementary Information 2). The length distribution of the 8–25-mer peptides indicated a typical HLA-I-restricted profile. We found that 9-mer peptides were the most abundant human peptides, but there were many longer peptides, especially 10–16-mers (Extended Data Fig. 1a).

Notably, identical peptide sequences were detected across multiple individuals, and 45 proteins shared the same peptide in 3–7 samples from the 7 *Pv*-infected individuals (Extended Data Fig. 1b,c). A sequence comparison between *Pv* and *Pf* proteomes showed that 34 of the 45 *Pv* peptide source proteins share more than 80% identity with *Pf* proteins (Extended Data Fig. 1b,c). Peptides from ribosomal protein (RP) 60S L4/L1 and ETRAMP (A5KBH5) appeared in all seven samples, with 83% and 63% identity with *Pf*, respectively. ETRAMP (A5K676), RP 40S S2 and Don Juan protein were detected in six out of seven samples and shared 43%, 97% and 53% identity with *Pf*, respectively. Peptides from 11 proteins were shared across 5 samples, mostly from the 40S RP family, with 80–100% identity to *Pf*. Additional proteins were shared across four donors (33–97% identity) and three donors (0–97% identity) (Extended Data Fig. 1b). An analysis of all 1,025 *Pv* peptides showed that the vast majority were derived from ribosomal proteins (64%), followed by uncharacterized proteins (11%), ETRAMP (7%), other proteins (6%), sperm-specific Don Juan protein (3%) and histones (3%) (Fig. 1b).

**Extended Data Fig. 2:**
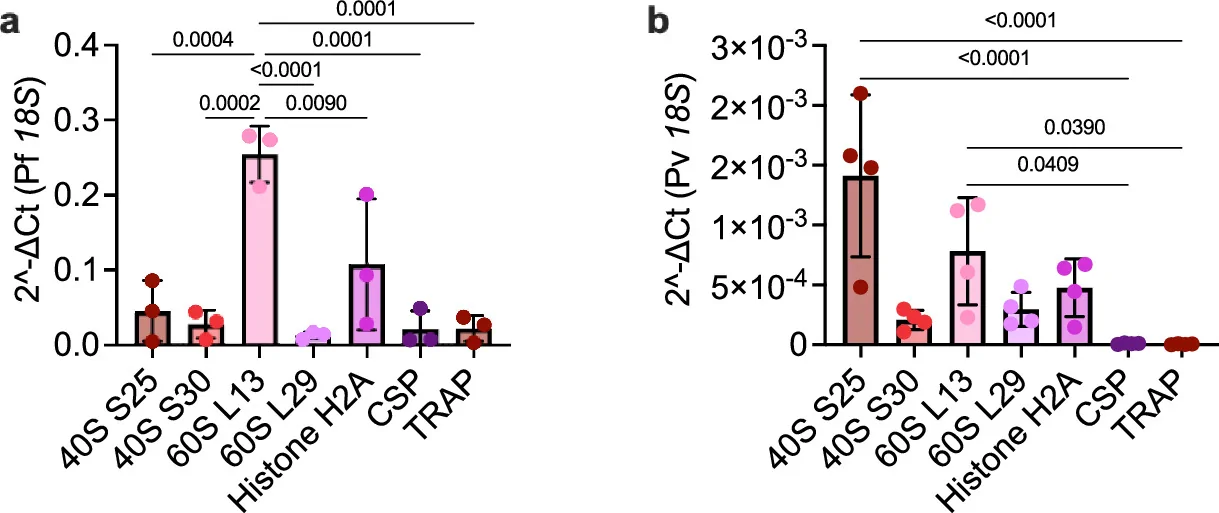
Antigen gene expression in humanized mouse livers infected with *Pf* or *Pv.* **a**,**b**, Expression of genes corresponding to antigens identified by *Pv* immunopeptidomics during liver stage infection with *Pf* (**a**) or *Pv* (**b**). Parasite 18S rRNA was used as endogenous control. CSP and TRAP genes served as positive control. Data are shown as mean ± s.e.m. Statistical analysis was performed using one-way ANOVA with Tukey’s multiple comparisons test; exact *P* values are indicated.

Given the dominance of housekeeping proteins (Supplementary Information 3), we investigated whether these proteins are expressed during other stages of the *Pv* life cycle, using published transcriptomic data for the blood (*Pv*Sal1 strain)^13–15^, liver (*Pv*P01 strain)^16^ and sporozoite (*Pv*Sal1 strain)^17^ stages. We found that all 166 genes encoding proteins are transcribed during the blood stage, 153 during the liver stage, 146 in hypnozoites and 125 in sporozoites (Supplementary Information 4). Similarly, we searched for *Pf* transcript orthologues in PlasmoDB^18^, the Malaria Cell Atlas^19^ and datasets of *Pf*-infected hepatocytes^20^. We found that 143 transcript orthologues are transcribed in the *Pf*3D7 blood stage, 143 in the liver stage and 135 in sporozoites (Supplementary Information 4). We further validated the expression of selected transcripts in vivo using quantitative PCR with reverse transcription (RT–qPCR) in humanized mice infected with *Pf* and *Pv* sporozoites (Extended Data Fig. 2). Representative members of the major protein families that were most enriched in our dataset (40S RP, 60S RP and histones) were evaluated, and CSP and TRAP were used as positive controls. The five housekeeping genes tested were expressed during the liver stage of both parasite species. Notably, *Pv* 60S RPL13 and 40S S25 showed higher expression than CSP and TRAP (Extended Data Fig. 2a). For *Pf*, the expression of 60S RP L13 and histone H2A exceeded that of CSP and TRAP (Extended Data Fig. 2b).

## Peptide validation in *Pv* and *Pf* samples

**Extended Data Fig. 3:**
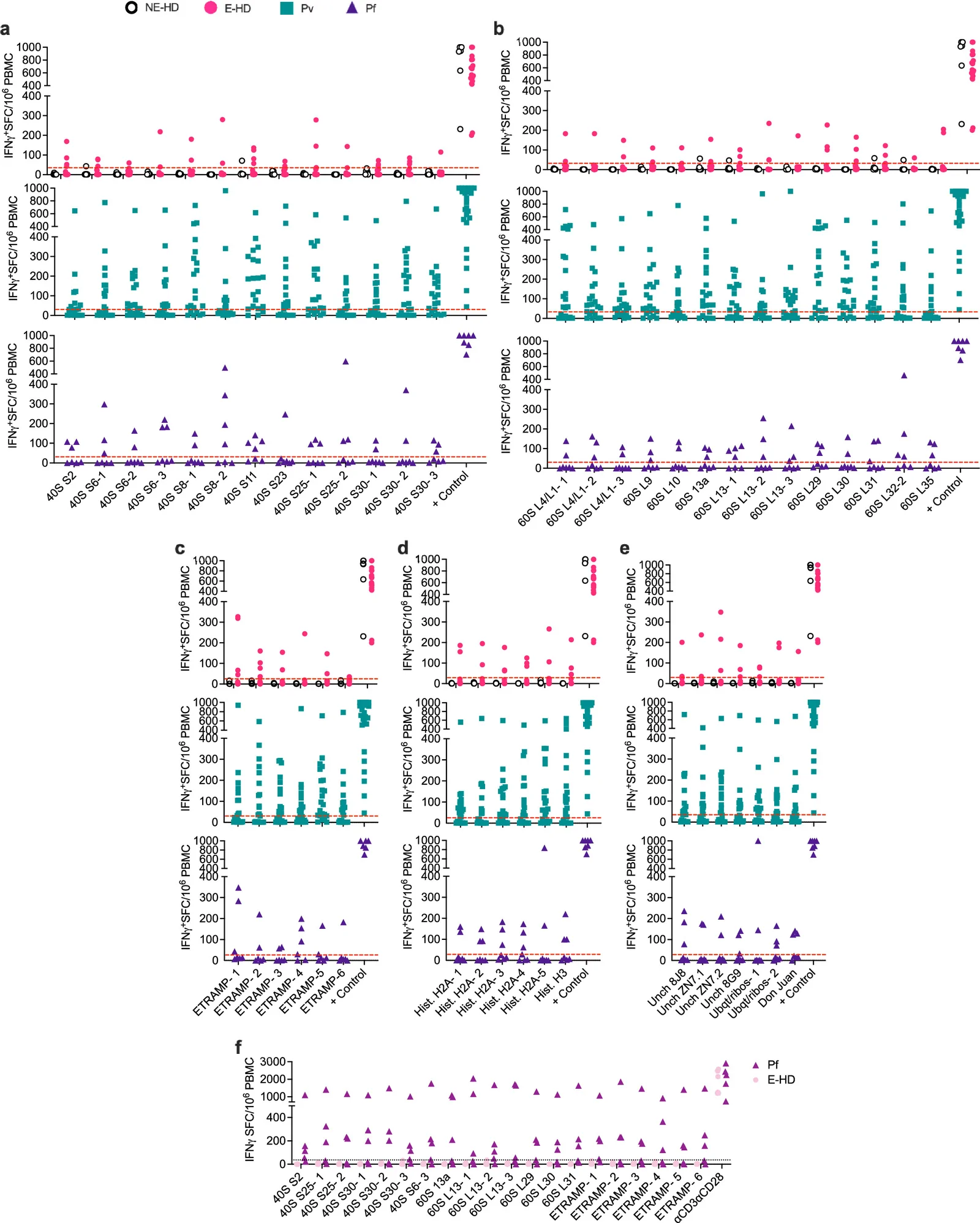
Peptide validation by ex vivo IFNγ ELISpot. **a**–**e**, PBMCs from a Brazilian cohort: *Pv*-infected (n = 24); *Pf*-infected (n = 7), endemic HD (n = 15); and non-endemic HD (n = 6). Cells were stimulated with peptides derived from **a**, 40S ribosomal proteins, **b**, 60S ribosomal protein, **c**, ETRAMPs, **d**, histone, and **e**, other proteins. **f**, PBMCs from a Malian cohort: *Pf*-infected individuals (n = 5) and endemic HD (n = 7). IFNγ responses are shown as SFC per 1 × 10^6^ PBMCs. Each symbol represents one individual. Responses ≥30 spots are considered positive (red dashed line). Anti-CD3/anti-CD28 stimulation was used as positive control.

To determine whether the identified peptides functioned as true T cell epitopes, we selected 50 peptides for further validation on the basis of the following criteria: (i) source proteins detected in three or more donors; (ii) identical peptide sequence found across multiple patients; and (iii) peptides with the highest Peaks score. All of the peptides selected (Supplementary Information 5) were synthesized and tested by spectral matching to their original biological *Pv* peptides (Supplementary Information 6). Forty-five of the 50 selected peptides were confirmed to have spectral matches and were submitted for functional assessment. Individual peptides, classified as ribosomal, ETRAMP, histone and other peptides, were tested in IFNγ ELISpot assays using peripheral blood mononuclear cells (PBMCs) from a Brazilian cohort of *Pv*-infected individuals (*n* = 24), *Pf-*infected individuals (*n* = 7) or endemic healthy donors (E-HD; *n* = 15), and non-endemic healthy donors (NE-HD; *n* = 6) (Fig. 1c,d and Extended Data Fig. 3a–e). The mean number of IFNγ-producing spot-forming cells (SFCs) from samples stimulated with the pool of 50 peptides was statistically higher in *Pv*-infected patients than in E-HD or NE-HD individuals, and in *Pf*-infected patients when compared with NE-HD individuals (Fig. 1c). There was no difference in the IFNγ-producing SFCs in samples from *Pv-* and *Pf-*infected patients. The same pattern was observed in the ELISpot results for ribosomal peptides: samples from *Pv-* and *Pf-*infected patients showed similar responses; *Pv*-infected patients had significantly higher responses than did E-HD or NE-HD individuals; and the responses of *Pf*-infected patients differed from those of NE-HD individuals (Fig. 1c). Samples from *Pv-*infected patients responded significantly more to ETRAMP, histone and other peptides, compared with samples from NE-HD individuals, whereas *Pf*-infected patients showed heightened but not statistically significant responses—potentially owing to the small sample size for *Pf* (*n* = 7). Individual peptide responses across all groups are shown in Extended Data Fig. 3a–e.

Heat maps of responder frequencies revealed broad recognition of both 60S ribosomal and 40S ribosomal peptides by *Pv-* and *Pf-*infected patients (Fig. 1d). The proportion of *Plasmodium-*infected patients who responded to ETRAMP, histone and other peptides was moderate. Notably, no NE-HD individual responded to any peptides, whereas some E-HD individuals with a previous history of *Pv* infection showed reactivity to some peptides (Fig. 1c,d).

We further evaluated peptide-specific T cell responses by flow cytometry. CD8^+^ T cells from *Pv*-infected individuals produced significantly higher levels of IFNγ after stimulation with the pooled peptides, ribosomal 40S, ribosomal 60S, ETRAMP, histone or other peptides, compared with the no-peptide control (Fig. 1e). CD4^+^ T cells responded only to the combined peptide pool (Fig. 1f), consistent with the HLA-I origin of the peptides. Functionally, antigen-specific CD8^+^ T cells had a varied profile, including CD69 upregulation, IL-2 production and CD107a expression (Fig. 1g).

We next asked whether responses differed between symptomatic and asymptomatic *Pv*-infected individuals. In our actively monitored Brazilian cohort, around one-third of infections were asymptomatic owing to naturally acquired immunity^21,22^. PBMCs from asymptomatic *Pv-*infected individuals exhibited IFNγ responses comparable with those of PBMCs from symptomatic donors when stimulated with the pooled peptides; by contrast, IFNγ responses were minimal or absent in PBMCs from NE-HD and E-HD individuals (Fig. 1h).

To validate these findings across independent geographical cohorts, we next evaluated PBMCs from *Pf*-infected individuals (*n* = 5) and local E-HD individuals (*n* = 7) in Mali. Twenty peptides were tested by IFNγ ELISpot (Fig. 1i and Extended Data Fig. 3f). Compared with E-HD individuals, *Pf*-infected people showed a significantly higher number of SFCs in response to 40S RP, 60S RP and ETRAMP peptides (Fig. 1i), supporting species-transcending antigenicity.

Moreover, peptide immunogenicity was evaluated in a controlled human malaria infection (CHMI) study in the USA, using blood-stage *Pv*. PBMCs collected before challenge (day 0) and at day 34 were stimulated with 29 peptides. Seventeen peptides elicited detectable IFNγ and/or TNF (cytokine-positive; Cyt^+^) CD8^+^ responses (Fig. 1j), and 7 peptides were recognized by at least 2 of the 6 volunteers (33%), highlighting the high frequency of antigenicity of the peptides even in volunteers with random HLA repertoires.

## *Pv* peptides bind to HLA-A, HLA-B, HLA-C and HLA-E

**Extended Data Fig. 4:**
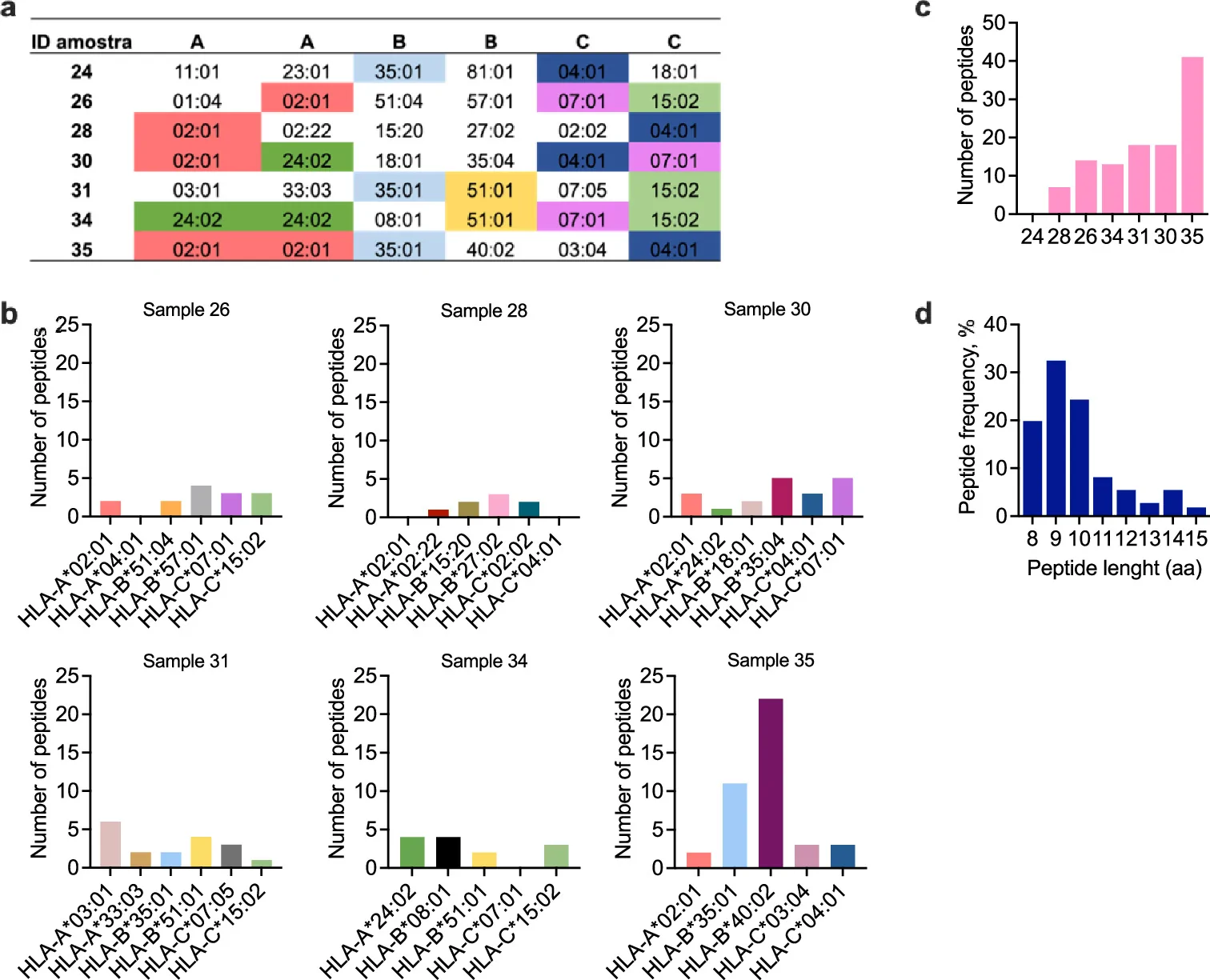
*Pv* peptides are presented by different HLA molecules. **a**, HLA-ABC genotyping of all 7 patients in the immunopeptidome analysis. **b**–**d**, NetMHC bind analysis of peptides identified in each sample to their donor HLA type. **b**, Number of peptides identified as HLA-ABC binders for each sample. HLA-ABC genotyping of the donor of samples used in the immunopeptidomics assays. Alleles represented with same colour mean that they were shared across different samples. **c**, Total number of peptides predicted to bind to each sample. Bars represent the overall peptide counts for each individual. **d**, Length distribution of binder peptides. Histogram shows the frequency of peptides according to their amino-acid length, highlighting the predominance of 8–11-mer peptides typical of HLA class I binding.

We were surprised that many of the peptides were recognized by most of the individuals. To determine whether the malaria peptides and proteins were presented by different HLA alleles, we HLA-typed each of the seven *Pv*-infected donors whose samples were used in the immunopeptidomics analyses (Extended Data Fig. 4a). The NetMHCpan-4.0^23^ tool was used to predict how many of the 50 chosen peptides were likely to bind to the classical HLA-A, HLA-B and HLA-C molecules of each donor (Extended Data Fig. 4b). Applying a relaxed 10% rank threshold, 6 donors showed the predicted binding of 5–40 peptides each, whereas donor 24 had no predicted binding peptides (Extended Data Fig. 4c). Predicted binders were mainly 8–10-mers, as expected for HLA-I peptides (Extended Data Fig. 4d).

**Fig. 2:**
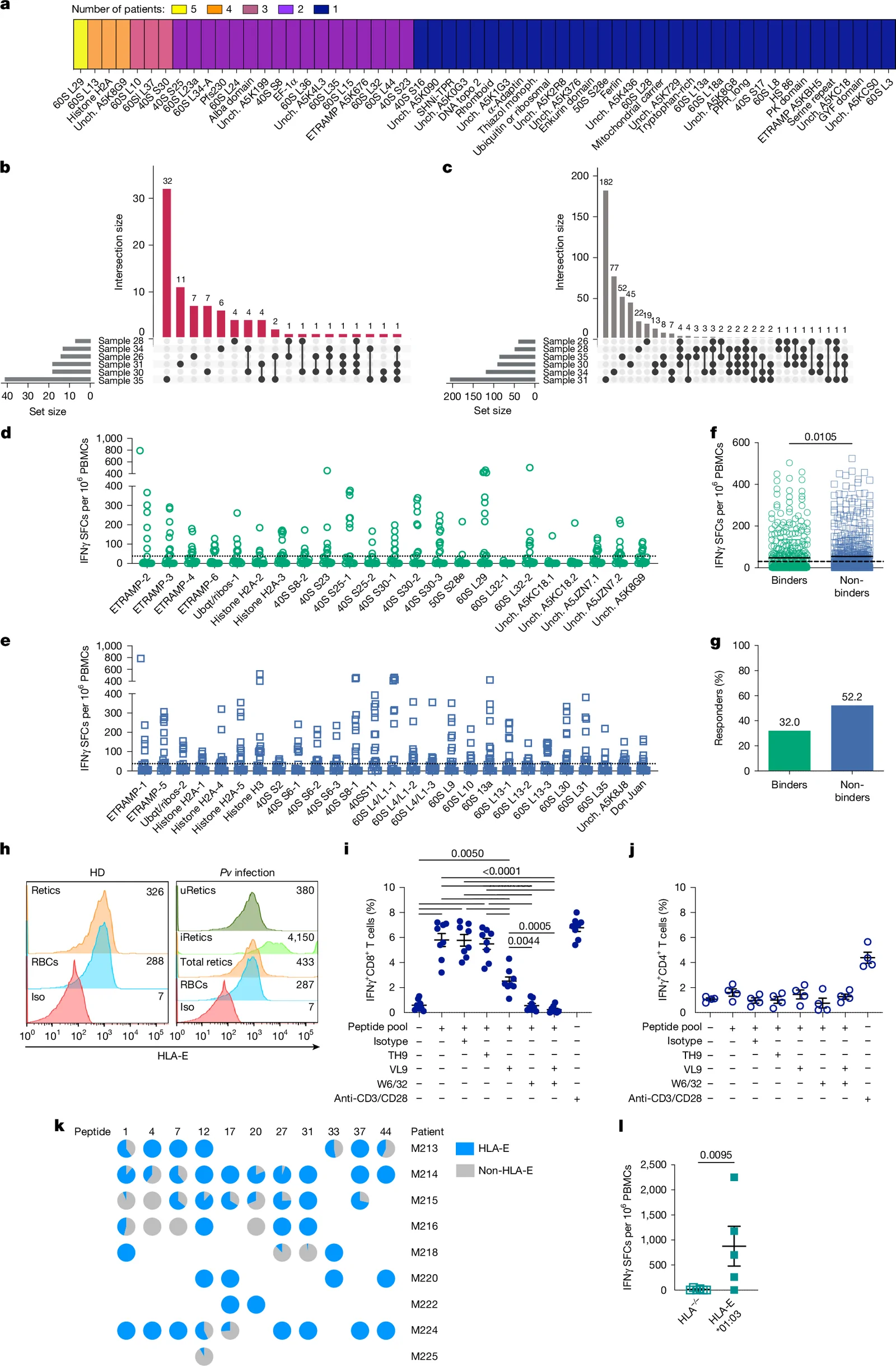
*Plasmodium* peptides are presented by classical and non-classical HLA-I molecules. **a**, Proteins containing peptides predicted to bind to classical HLA-I molecules (HLA-A, HLA-B or HLA-C) were identified using NetMHC. Colour intensity indicates the number of patients for whom binding was predicted. HS, heat shock; monoph., monophosphate; PK, protein kinase. **b**,**c**, UpSet plots showing the intersections of *Pv*-derived peptides identified across samples. Columns represent peptide intersections; rows represent individual samples. Horizontal bars indicate total peptides per sample. Connected dark circles indicate shared peptides; single circles represent peptides unique to one sample. **b**, *Pv* peptides. **c**, Human peptides. **d**–**g**, Anti-IFNγ ELISpot responses of *Pv*-infected patients to individual peptides predicted to bind (**d**) or not (**e**) to classical HLA-I. Data are expressed as SFCs per 10^6^ PBMCs; the dashed line denotes the positivity threshold (≥30 SFCs). **f**, IFNγ ELISpot response to all peptides pooled and subdivided into binders versus non-binders. **g**, Percentage of *Pv*-infected patients with positive ELISpot responses (binders and non-binders). **h**, Histogram of HLA-E surface expression on uninfected reticulocytes (uRetics), uninfected RBCs and *Pv-*infected reticulocytes (iRetics); MFI values are shown. Iso, isotype control. **i**,**j**, PBMCs from *Pv*-infected patients (*n* = 8) were incubated with blocking reagents targeting HLA-E (VL9 peptide) or pan-HLA-I (W6/32 monoclonal antibody), or their respective controls (TH9 peptide or isotype monoclonal antibody), then stimulated with the 50-peptide pool. IFNγ production by CD8^+^ T cells (**i**) and CD4^+^ T cells (**j**) was measured by ICS. No peptide and anti-CD3/anti-CD28 conditions served as negative and positive controls. **k**, Pie charts summarizing HLA-E-dependent (VL9 blocked) versus non-HLA-E-dependent responses for individual peptides across donors. **l**, IFNγ ELISpot assay assessing HLA-E-restricted CD8^+^ T cell responses: purified CD8^+^ T cells were primed with the peptide pool and cocultured with peptide-pulsed HLA-deficient K562 cells expressing HLA-E*01:03. Responses are shown as SFCs per 10^6^ CD8^+^ T cells. Statistical analyses: Mann–Whitney test (**f**,**l**) and Tukey’s multiple comparisons test (**i**,**j**). Mean ± s.e.m. and exact *P* values are shown.

Despite distinct HLA haplotypes, many identical or nested peptides were predicted to bind to several HLA molecules (Supplementary Information 7). For example, peptides from 60S RP L29 had predicted high affinity to HLA molecules from five individuals, and peptides from 60S RP L13, histone H2A and uncharacterized proteins (A5K8G9) were predicted to bind to HLA molecules from four individuals. Peptides from 60S RP L10, 60S RP L37 and 40S RP S30 were predicted to bind to HLA molecules from three donors. Seventeen antigens had predicted high binding affinity for HLA-A, HLA-B and HLA-C from two donors (Fig. 2a and Supplementary Information 7).

To further assess overlap, we performed an intersection analysis of predicted binders (Fig. 2b), in which each vertical bar represents the number of shared peptides in a specific sample intersection, and the connected dots below indicate which samples share the same peptides predicted to bind to their HLA. Sample 24 was not included in this analysis, because it did not share any peptide that was predicted to bind to HLA-A, HLA-B or HLA-C (Fig. 2b). The first six red bars, which do not form intersections, represent peptides that were predicted to bind exclusively to the HLA-A, HLA-B or HLA-C molecules of individual samples. Specifically, 32 peptides were predicted to bind exclusively to the HLA-A, HLA-B or HLA-C of sample 35, and 11 peptides were predicted to bind exclusively to the HLA-A, HLA-B or HLA-C of sample 31. Samples 26 and 30 each exhibited seven unique predicted HLA-A-, HLA-B- or HLA-C-binding peptides, whereas six peptides were unique to sample 34 and four peptides were unique to sample 28. With respect to shared peptide repertoires, the largest intersections among *Pv* peptides were observed between samples 34 and 30, which shared four predicted HLA-A, HLA-B or HLA-C binders, and between samples 31 and 35, which also shared four peptides. Intersections of one or two peptides were observed across several donor combinations. This analysis reveals both common and unique peptide repertoires across the cohort, and suggests that immunodominant epitopes exist that drive shared immune responses.

A similar intersection analysis was performed for human-derived peptides identified in this study (Fig. 2c). A total of 182 human peptides bound exclusively to the HLA molecules of donor 31, 77 to donor 34, 52 to donor 35 and smaller sets of peptides to donors 30, 28 and 26. Human peptides also showed several shared intersections, including 13 peptides between donors 30 and 34 and 8 between donors 30 and 35. Together, these results show that human antigenic epitopes are less likely to be shared than are malaria epitopes.

Because shared malarial peptide sequences across donors might arise from contamination and coprecipitation, or from presentation by distinct HLA alleles, we stratified peptides into binders and non-binders using NetMHCpan predictions. Of the 50 peptides tested in the ELISpot assay, 23 were predicted binders (Fig. 2d) and 27 were predicted non-binders (Fig. 2e). Notably, non-binder peptides had significantly higher IFNγ responses than did binder peptides (Fig. 2f), and triggered T cell responses in a greater proportion of donors (55.2% versus 32%) (Fig. 2g), indicating that most of the immunostimulatory malarial peptides are not predicted to bind to the classical HLA-A, HLA-B and HLA-C.

**Extended Data Fig. 5:**
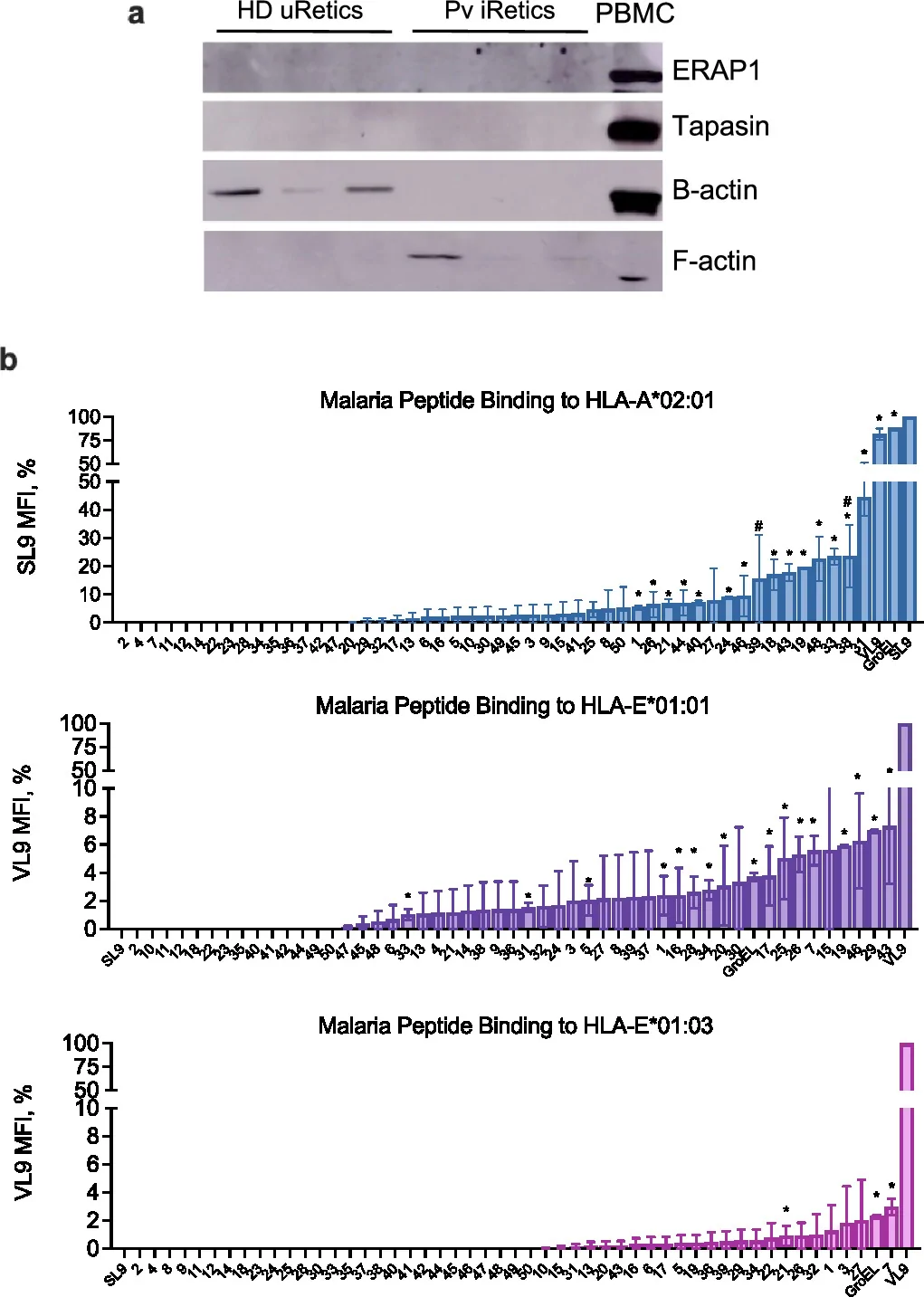
Expression of tapasin and ERAP1 on reticulocytes. **a**, Western blot of cell lysates from uninfected reticulocytes (uRetics) from three healthy donors, and cell lysates from *Pv*-infected reticulocytes (*Pv* iRetics) from three patients, were probed for the antigen-processing protein tapasin and ERAP1. PBMC was used as a positive control. Anti-β-actin and anti-F-actin were used as endogenous protein expression control for uninfected and infected cells, respectively. **b**, In vitro binding stability assay of all selected peptides pulsed in an HLA-A*02:01, HLA-E*01:01, and HLA-E*01:03 cell lines, including control peptide (SL9 for HLA-A*02:01 and VL9 for HLA-E*01:01 and HLA-E*01:03). Peptides marked with (*) whose mean MFI are higher than s.d. are considered to be binders. Peptides marked with (#) were in silico predicted to be HLA-A*02:01 binders. Source data

Because peptides that were not expected to bind to the canonical HLA class I had higher IFNγ responses and unusually long peptides were eluted from the HLA proteins of *Pv*-infected reticulocytes, we investigated whether this finding could be explained by a deficiency in the expression of antigen-presentation-pathway proteins in reticulocytes, which might favour the loading of unconventional peptides. Although reticulocytes express TAP^3^, neither tapasin nor ERAP1 was detected in uninfected or infected reticulocytes (Extended Data Fig. 5a)—suggesting that there is an antigen-processing abnormality that affects peptide quality control and trimming in reticulocytes.

Given this altered processing machinery, we next assessed whether non-canonical antigen-presentation pathways might contribute to the observed responses—specifically, the non-classical HLA class I molecule HLA-E. HLA-E typically presents a leader sequence^24–26^, but during infection or stress, it can present longer, low-affinity peptides^27–31^. We found that HLA-E is expressed on reticulocytes and upregulated on infected reticulocytes (Fig. 2h), as previously reported^3^ for pan-HLA-I. HLA-E–peptide complexes were also captured during our immunopeptidomics analysis through W6/32 immunoprecipitation, and HLA-E was identified by mass spectrometry. Notably, no VL9 peptides were detected, including sequence variants VMAPRT(L/V)(V/L/I/F)L (refs. ^24–26^) (Supplementary Information 2). This prompted us to investigate whether the screened malaria peptides could be presented by HLA-E.

PBMCs from *Pv*-infected individuals were stimulated either with the peptide pool or with individual peptides, and were treated with either a W6/32 monoclonal antibody (pan-HLA class I blockade) or the VL9 peptide (selective blockade of HLA-E binding), together with appropriate controls (an isotype control antibody or an irrelevant control peptide (TH9; TVCVIWCIH), respectively) (Fig. 2i,j). As expected, W6/32 abolished the production of IFNγ in CD8^+^ T cells IFNγ. Of note, VL9 significantly reduced the production of IFNγ, by 50% (Fig. 2i). Because CD4^+^ T cells did not produce IFNγ (Fig. 1f), the blocking conditions had no effect in these cells (Fig. 2j). Testing of individual peptides confirmed that CD8^+^ T cell responses were consistently impaired after HLA-E blockade (Fig. 2k).

To directly assess whether HLA-E-expressing cells activate *Pv*-primed CD8^+^ T cells, isolated CD8^+^ T cells were primed with the peptide pool and cocultured with peptide-pulsed K562 cells lacking all classical HLA (HLA^–/–^) or engineered to express HLA-E*01:03 (Fig. 2l). Strong IFNγ responses were observed only with HLA-E-expressing targets, confirming that HLA-E-presented peptides elicit the production of IFNγ in CD8^+^ T cells.

Notably, no VL9-like peptides were identified in the immunopeptidomics dataset (Supplementary Information 2). Because algorithms for predicting HLA-E-binding peptides are trained on VL9-like motifs, their performance on non-canonical pathogen-derived peptides is limited. We next performed an in vitro binding stability assay in mono-allelic HLA-A*02:01, HLA-E*0101 and HLA-E*0103 cell lines (Extended Data Fig. 5b). The mean fluorescence intensity (MFI) of each peptide was normalized to the MFI of the known HLA binder (SL9 for HLA-A*02:01 and VL9 for HLA-E*01:01 and HLA-E*01:03) and ranked^32^. Despite their capacity to stimulate CD8^+^ T cells, the 17 *Pv* peptides that bound to HLA-E*01:01 or HLA-E*01:03 had a binding score at least 1-log lower than the positive control VL9, and 13 peptides also showed measurable binding for HLA-A*02:01.

Notably, a *Salmonella*-derived peptide (GroEL), which is known to bind to HLA-E and elicit strong CD8^+^ T cell responses^33^, also appeared weak in this assay (Extended Data Fig. 5b). Consistent with studies^23,34^ showing that antigenic pathogen-derived peptides have low binding scores and/or affinity to HLA-E, several *Pv* peptides that exhibited low or undetectable in vitro binding nonetheless induced functional CD8^+^ T cell responses that were eliminated under HLA-E blockade (Fig. 2k).

Together, these findings suggest that the broad CD8^+^ T cell recognition observed across *Pv*-infected donors reflects the simultaneous presentation of parasite peptides by multiple HLA-I molecules, including both the classical HLA-A, HLA-B and HLA-C and the non-classical HLA-E. The atypical antigen-processing environment of reticulocytes, combined with the low polymorphism of HLA-E, provides a mechanistic explanation for why many peptides are shared, immunogenic and recognized across individuals with diverse HLA genotypes.

## Peptide-specific CD8^+^ T responses in NHPs

**Fig. 3:**
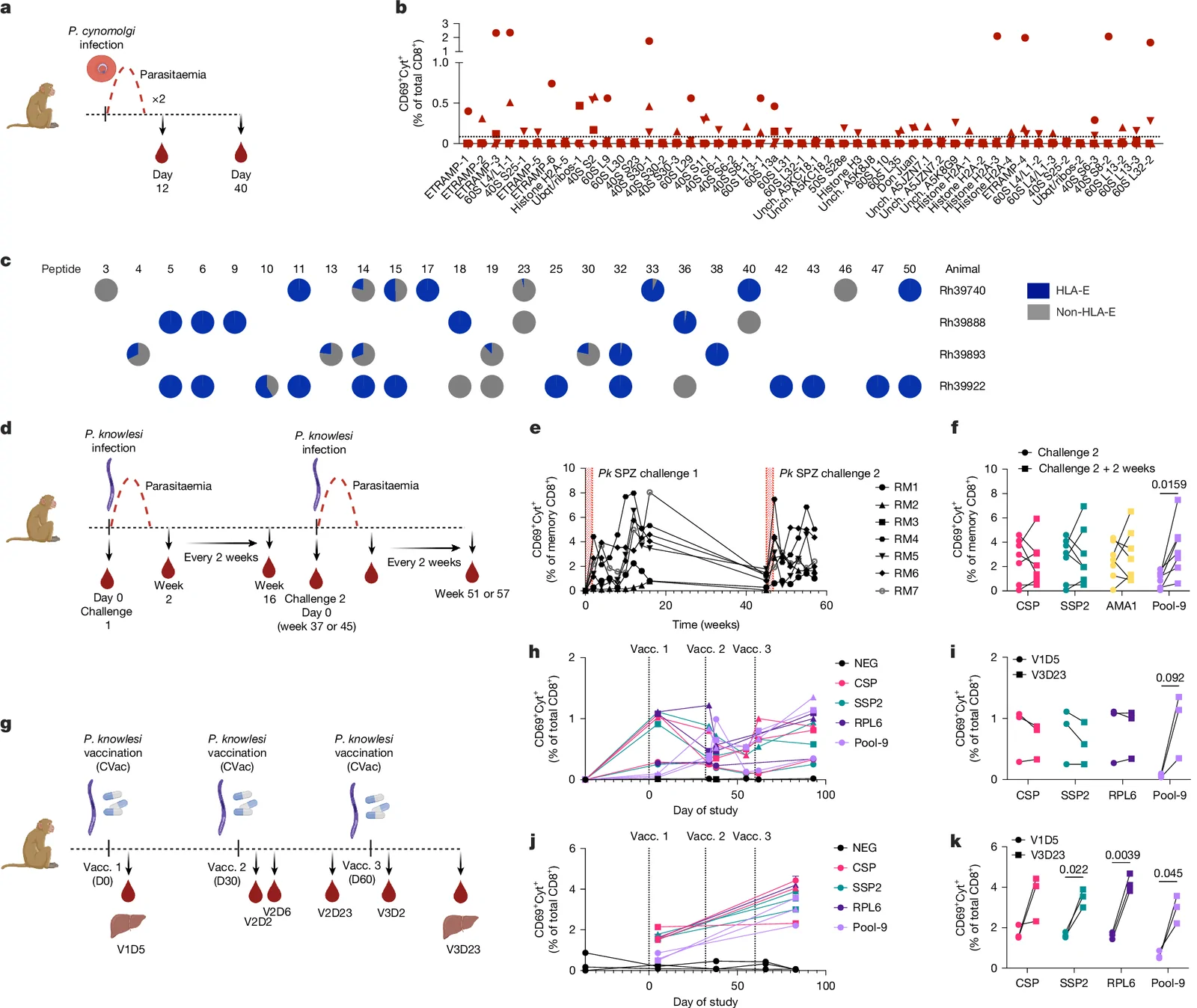
Novel *Plasmodium* antigens are recognized by T cells elicited during *Pcy* and *Pk* infections in NHPs. **a**, Repeated *Pcy* blood-stage infection of rhesus macaques (*n* = 4). Macaques received cryopreserved *Pcy* Berok-infected RBCs intravenously. Peripheral blood was collected 12 and 40 days after infection, and infection was repeated after at least two months. **b**, CD8^+^ T cell responses to 50 individual peptides measured 12 days after *Pcy* infection. Responding CD8^+^ T cells expressed CD69 and IFNγ and/or TNF (Cyt^+^); responses are shown after subtraction of DMSO controls. Each symbol represents one macaque. **c**, Proportion of HLA-E-restricted CD8^+^ responses after secondary *Pcy* infection. Antigens eliciting primary responses were retested ± HLA-E-blocking peptide (VL9). Responses at day 12 (macaques 39740, 39888 and 39992) or day 40 (macaque 39893) are shown (blue, HLA-E-restricted; grey, non-restricted). **d**, Repeated *Pk* sporozoite infection of rhesus macaques (*n* = 7). Macaques received 2,500 cryopreserved *Pk* sporozoites intravenously; parasitaemia higher than 2% triggered treatment on days 10–12. PBMCs were collected every two weeks. **e**, CD8^+^ T cell responses (from PBMCs) to a pool of nine conserved *Pv* and* Pk* peptides (pool-9) after two sporozoite (SPZ) infections. Responding CD8^+^ T cells expressed CD69 and IFNγ and/or TNF (Cyt^+^); longitudinal responses are shown per macaque (RM; rhesus macaque), with pink shading indicating parasitaemia. **f**, Pairwise comparison of CD8^+^ T cell responses (from PBMCs) to overlapping peptides from the indicated *Pk* antigens (CSP, SSP2 or AMA1) or pool-9 after secondary infection. **g**, CVac: rhesus macaques (*n* = 3) received three monthly intravenous inoculations of 50,000 *Pk* sporozoites under chloroquine prophylaxis plus Coartem treatment on days (D) 7–9. **h**,**i**, Longitudinal (**h**) and pairwise (**i**) analyses of peripheral-blood CD8^+^ T cell responses to overlapping peptides in after CVac. **j**,**k**, Longitudinal (**j**) and pairwise (**k**) analyses of intrahepatic CD8^+^ T cell responses after CVac. NEG, negative control. Statistical analyses: paired *t*-test (**f**,**i**,**k**). Exact *P* values are shown. Illustrations in **a**,**d**,**g** created in BioRender; Junqueira, C. https://BioRender.com/w4s73tg (2026). Source data

**Extended Data Fig. 6:**
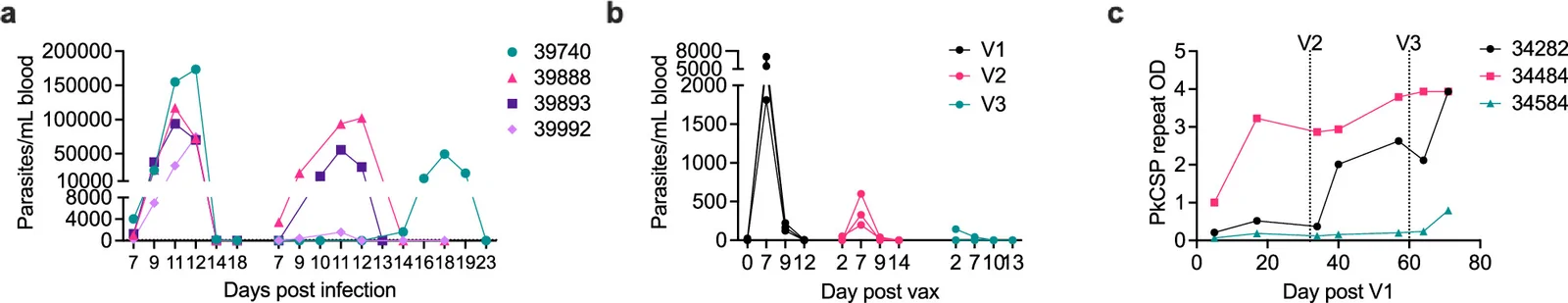
Parasitaemia during *Pk* and *Pcy* infection of NHPs. **a**, Parasitaemia of *Pk* as measured by qRT–PCR after each immunization. **b**, Antibodies to *Pk* CSP repeat region throughout immunization as measured by single dilution ELISA OD value. **c**, Parasitaemia of *Pcy* as measured by Giemsa-stained thin smears.

To assess CD8^+^ T cell cross-species responses to these novel antigens, we used the NHP model of *Pcy*, which recapitulates key features of *Pv*, including tropism for reticulocytes and hyponozoite formation, and can also infect humans^35,36^. Rhesus macaques (*n* = 4) were infected twice by *Pcy* blood-stage inoculation, with three months between infections (Fig. 3a). Parasitaemia was confirmed for all macaques (Extended Data Fig. 6a).

The responses of CD8^+^ T cells to 50 peptides were determined by intracellular cytokine staining (ICS) of peptide-stimulated PBMCs during acute primary infection. At least 35 out of 50 peptides elicited responses in at least one macaque, with variable magnitude (Fig. 3b). During the second infection, PBMCs were stimulated with previously recognized peptides in the presence or absence of the VL9 peptide, which binds to the rhesus macaque homologue of HLA-E (Mamu-E)^37^. Resembling our human data (Fig. 2l), the responses of CD8^+^ T cells to multiple peptides were fully or partially blocked by VL9, indicating dependence on Mamu-E (Fig. 3c). Responses to specific peptides (14, 18, 36 and 40; Supplementary Information 5) were blocked by VL9 in some macaques but not in others. These data indicate that a substantial fraction of the CD8^+^ T cell responses to conserved peptides after *Plasmodium* infection are Mamu-E restricted and not strictly peptide dependent.

We next assessed peptide antigenicity in a sporozoite-induced infection model using *Pk*, which more closely reflects *Pf* infection, with an approximately six-day liver stage and no reticulocyte restriction. Rhesus macaques (*n* = 7) were inoculated intravenously with 2,500 cryopreserved *Pk* sporozoites on two occasions and developed high levels of parasitaemia after each challenge (Fig. 3d). Given the limited cell availability, CD8^+^ T cell responses were monitored over one year during two sporozoite challenges, using a pool of nine peptides (pool-9; peptides 1, 4, 7, 20, 27, 31, 32, 33 and 37; Supplementary Information 5) that were chosen on the basis of them being completely conserved in *Pk* and highly antigenic in humans (Supplementary Information 4). All macaques developed CD8^+^ T cell responses to pool-9 with variable magnitude and durability. Responses persisted up to 16 weeks after challenge and stabilized before the second challenge (37 or 45 weeks later) (Fig. 3e). After rechallenge, the responses of CD8^+^ T cells to pool-9 showed an anamnestic boost (Fig. 3f). By contrast, responses to overlapping peptides from canonical known antigens (CSP, SSP2 and AMA1) did not show comparable boosting (Fig. 3f).

Because there is evidence that these proteins are expressed during the liver stage^38^ (Extended Data Fig. 2), we next investigated immune responses after liver-directed immunization. Chemoprophylaxis vaccination (CVac), involving the intravenous administration of live sporozoites under drug cover, generates a robust, highly protective immunity, producing sterilizing protection in mice and NHPs^6,39^. Here, rhesus macaques (*n* = 3) were immunized with 5 × 10^4^ cryopreserved *Pk* sporozoites, three times at monthly intervals, with longitudinal sampling of blood and liver biopsies (Fig. 3g).

Consistent with previous observations^40^, most macaques developed functional liver-directed immunity, indicated by reduced or absent parasitaemia through immunizations (Extended Data Fig. 6b) and induction of anti-CSP IgG (Extended Data Fig. 6c). The responses of CD8^+^ T cells to established pre-erythrocytic antigens (CSP, SSP2 and RP L6) and to the newly identified pool-9 were monitored in blood and liver (Fig. 3h–k). Responses to established antigens were detectable in peripheral blood after the first immunization, but showed limited boosting with subsequent immunizations (Fig. 3h,i). This is consistent with previous reports, which found that repeated immunization did not enhance the number of circulating CD8^+^ T cells, owing to limited recirculation of liver-resident memory T cells^39–42^.

By contrast, pool-9 elicited CD8^+^ T cell responses that were significantly boosted by subsequent immunizations in both blood and liver (Fig. 3i,k). An analysis of intrahepatic CD8^+^ T cells at two time points (day 5 after vaccine 1 (V1D5) and day 24 after vaccine 3 (V3D24)) confirmed increased antigen-specific responses after repeated immunizations for all antigens except CSP (Fig. 3k). The lack of CSP boosting was driven mainly by a single macaque with unchanged responses across immunizations (Fig. 3j,k). These results show that a subset of newly identified antigens is conserved in *Pk* and elicits CD8^+^ T cell responses in both sporozoite-induced infection and liver-directed immunization.

## Activation of mouse CD8^+^ T cells by novel antigens

**Fig. 4:**
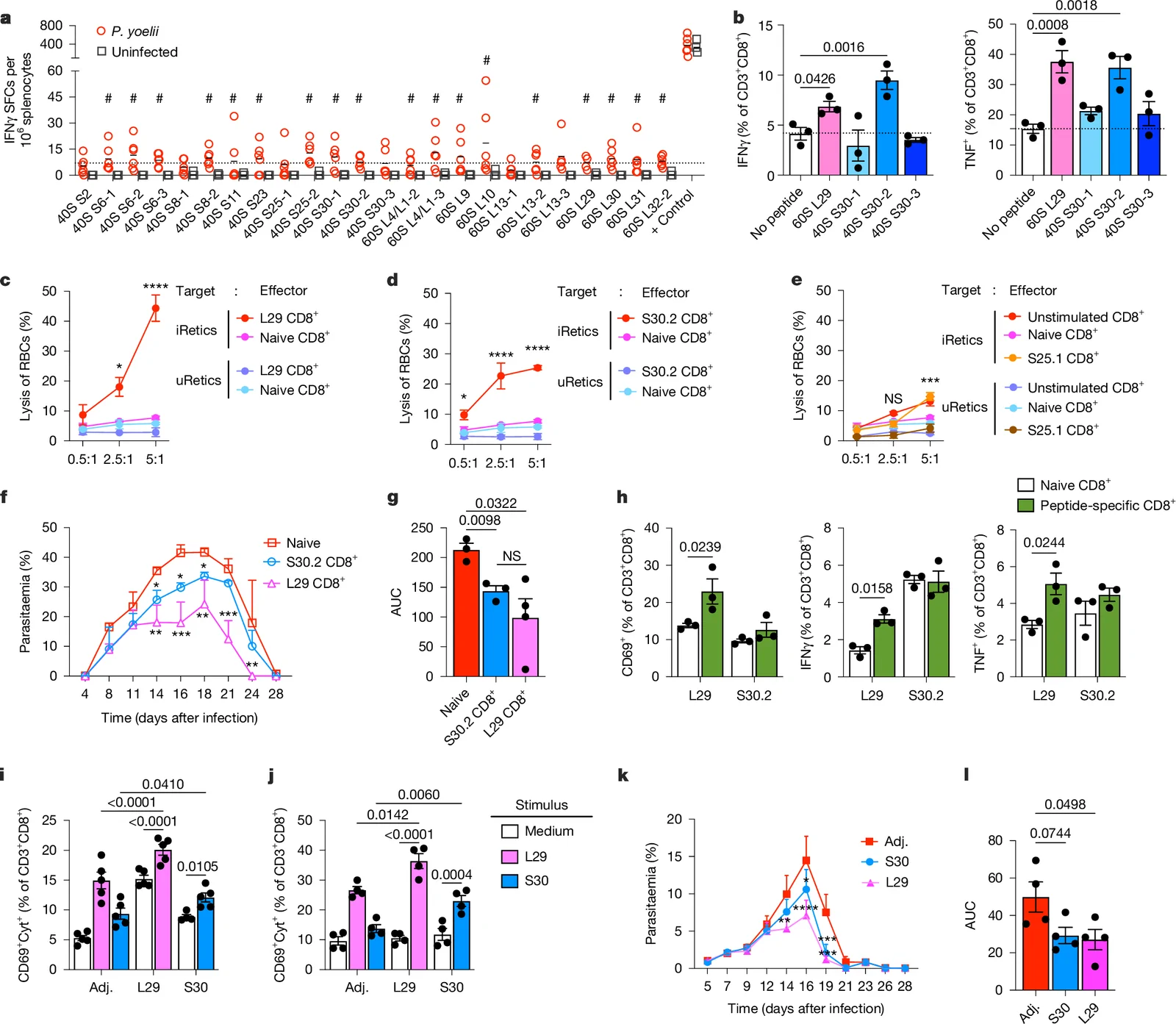
Immunogenic and protective validation of novel *Plasmodium* antigens in the *Py* model. **a**, IFNγ ELISpot responses of splenocytes from *Py-*infected mice (*n* = 6) or uninfected controls (*n* = 3) stimulated at day 12 after infection, after stimulation with the indicated peptides. Results are expressed as SFCs per 10^6^ splenocytes. The dashed line indicates the positivity threshold (>7 SFCs); peptides were considered positive (#) when the group average was higher than 7 spots. **b**–**e**, Generation and functional characterization of antigen-specific CD8^+^ T cells. **b**, IFNγ and TNF production by CD3^+^CD8^+^ T cells after in vitro expansion and restimulation with the indicated peptides. **c**–**e**, Cytotoxicity assays showing lysis of *Py*-infected reticulocytes or uninfected reticulocytes by L29.1-specific (**c**), S30.2-specific (**d**) and S25.1 (non-specific) or unstimulated (**e**) CD8^+^ T cells at increasing effector-to-target ratios. **f**–**h**, Adoptive transfer of antigen-specific CD8^+^ T cells. Mice (*n* = 4 per group) were challenged with 10^5^ *Py*-infected RBCs and received 1.5 × 10^6^ antigen-specific CD8^+^ T cells on days 0 and 4 after infection. **f**, *Py* parasitaemia was monitored for 28 days. **g**, Parasitaemia represented as area under the curve (AUC). **h**, Activation (CD69) and cytokine production (IFNγ and/or TNF: Cyt^+^) by splenic CD8^+^ T cells at 30 days after infection. **i**–**l**, Protective immunity induced by immunization with recombinant ribosomal proteins (L29 or S30). Mice (*n* = 10 per group) were immunized three times with 60S RP L29 (L29) or 40S RP S30 (S30) plus adjuvant, or with adjuvant alone (Adj.), with a 14-day interval. Antigen-specific CD8^+^ T cell responses in the liver (**i**) and in the spleen (**j**) were assessed by ICS. **k**, *Py* parasitaemia curve after blood-stage *Py* challenge. **l**, Parasitaemia represented as AUC. Statistical analyses: one-way analysis of variance (ANOVA) followed by Sidak’s multiple comparisons test (**b**); two-way ANOVA followed by Sidak’s multiple comparisons test (**c**–**e**,**h**–**j**), Tukey’s multiple comparisons test (**f**,**k**), or Dunnett’s multiple comparisons test (**l**); and unpaired *t-*test (**g**). Mean ± s.e.m. *P* values are shown either as the exact values or with asterisks: *P < 0.05, **P < 0.01, ***P < 0.001, ****P < 0.0001; NS, not significant. All experiments were performed at least twice with similar results. Source data

**Extended Data Fig. 7:**
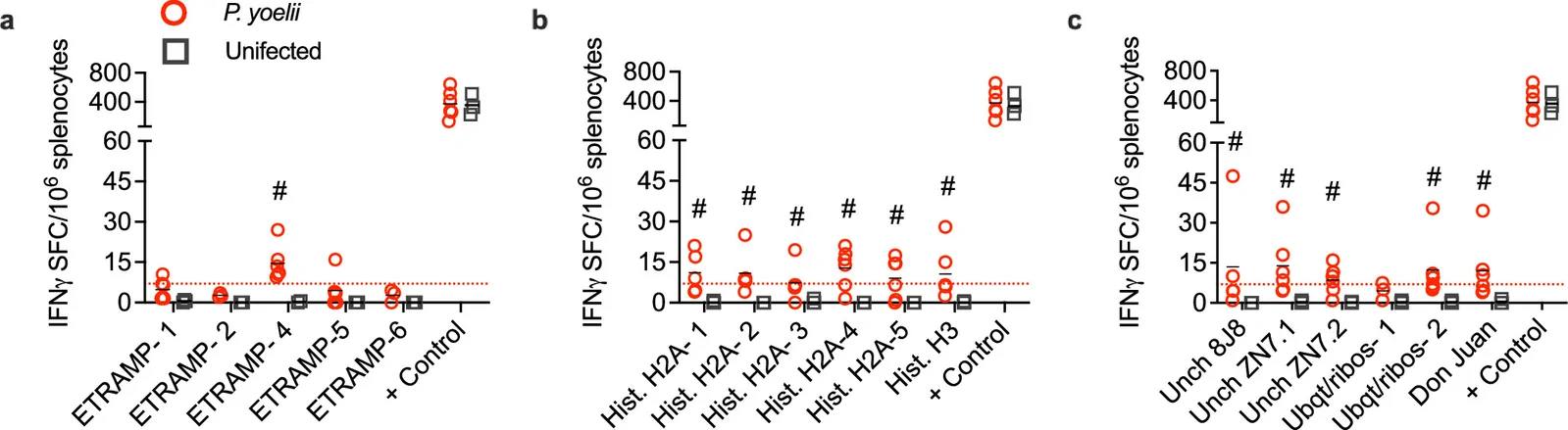
Peptide validation by *ex vivo* IFNγ ELISpot in a mouse model. **a–c**, Splenocytes from *Py*-infected mice were isolated at day 12 after infection following intraperitoneal inoculation of 10^5^ infected reticulocytes and stimulated with peptides derived from **a**, ETRAMPs, **b**, Histone and **c**, other proteins. Red symbols indicate infected mice (n = 6) and black symbols indicate uninfected controls (n = 3). Each symbol represents one mouse. IFNγ responses are shown as SFCs per 10^6^ splenocytes. Responses ≥10 SFC are considered positive (#, red dashed line). Statistical analysis was performed using multiple unpaired Student’s *t*-tests; *P* values are indicated.

Peptide antigenicity was further validated in a mouse model, using the reticulocyte-prone, non-lethal *Py* 17XNL strain. Mice were infected with *Py*-infected reticulocytes, and splenocytes were collected at peak parasitaemia (day 12) for ex vivo stimulation with individual peptides. IFNγ-producing cells were quantified by ELISpot (Fig. 4a and Extended Data Fig. 7a–c). Approximately 75% of the RP-derived peptides elicited stronger IFNγ responses in infected mice than they did in uninfected controls. Among non-RP peptides, one out of six ETRAMP peptides, all six histone-derived peptides and five out of six peptides from other protein classes were antigenic (Extended Data Fig. 7a–c).

To define the role of antigen-specific CD8^+^ T cells during the blood stage of *Py* infection, we selected two RPs—60S L29 and 40S S30—for in-depth characterization. These antigens were prioritized on the basis of (i) detection across multiple immunopeptidomics datasets (at least five samples; Supplementary Information 2 and Extended Data Fig. 1b,c); (ii) expression during the blood and liver stages (Extended Data Fig. 2); (iii) predicted binding to HLA-I (Fig. 2a); and (iv) immunogenicity across human and animal models (Supplementary Information 8 and Figs. 1c,d, 3b and 4a).

Peptide-specific CD8^+^ T cells were generated by repeated in vitro stimulation of splenocytes from *Py*-infected mice with L29.1 or S30.2 ribosomal peptides in the presence of IL-2. After stimulation, both populations produced increased levels of IFNγ, relative to unstimulated controls (Fig. 4b). Cytotoxic activity was assessed using in vitro killing assays across a range of effector-to-target ratios^43^. L29.1- and S30.2-specific CD8^+^ T cells lysed *Py*-infected reticulocytes efficiently in an effector-dependent manner (Fig. 4c–e). Cytotoxicity was antigen specific, because naive CD8^+^ T cells and uninfected reticulocytes showed minimal activity. CD8^+^ T cells that were cultured with IL-2 alone or stimulated with a non-responsive-peptide (S25.1) exhibited limited killing, significant only at the highest ratio tested (5:1) (Fig. 4e).

To assess in vivo function, L29.1- or S30.2-specific CD8^+^ T cells, or naive CD8^+^ T cells, were adoptively transferred into naive mice before challenge with *Py*-infected reticulocytes. Transfer of antigen-specific CD8^+^ T cells reduced parasitaemia, compared with naive controls, with L29.1-specific cells showing greater efficacy than S30.2-specific cells (Fig. 4f,g). After infection, transferred L29.1-specific CD8^+^ T cells were activated and produced IFNγ and TNF, confirming functional engagement in vivo (Fig. 4h).

Given that CD8^+^ T cells specific to a single peptide were sufficient to reduce parasitaemia, we next assessed whether immunization with full-length proteins could elicit protective responses. C57BL/6 mice were immunized three times with recombinant 60S L29 or 40S S30 proteins formulated with the vaccine adjuvants alum and CpG. Antigen-specific CD8^+^ T cell responses were detected in spleen and liver, characterized by activation (CD69^+^) and cytokine production (IFNγ and TNF) (Fig. 4i,j). After challenge with *Py*-infected reticulocytes, both antigens reduced peak parasitaemia, and L29 reduced the overall parasite burden significantly (Fig. 4k,l).

Together, these findings identify malarial RPs as conserved antigens that induce functional, cytotoxic CD8^+^ T cell responses across several *Plasmodium* species and experimental systems, supporting their potential as broadly protective malaria vaccine targets.

## Discussion

This study provides unbiased identification and validation of *Plasmodium* HLA-I-associated peptides presented on infected human reticulocytes, constituting what is, to our knowledge, the largest immunopeptidomics dataset for any *Plasmodium* species or stage. Combining high-resolution mass spectrometry and a strict bioinformatic pipeline^11^, we defined parasite peptides presented by HLA-I during *Pv* blood-stage infection, which enabled the direct discovery of physiologically relevant CD8^+^ T cell targets. Immunopeptidomics has enabled novel antigen discovery in cancer and infectious diseases^44–49^. Similar approaches in parasitic infections, such as *Leishmania*^48^ and *Trypanosoma cruzi*^50^, have identified T cell antigens with proven antigenicity. Our findings establish immunopeptidomics as a scalable approach for the discovery of T cell antigens in malaria.

We focused on HLA-I antigen presentation to CD8^+^ T cells, because reticulocytes lack HLA-II (ref. ^3^). From our 7 samples, we identified 7,843 HLA-I-bound peptides, of which 1,025 were parasite-derived and 453 were unique, mapping to 166 *Pv* proteins. Many peptide sequences were shared across donors, despite HLA polymorphism. Consistent with their high degree of sequence conservation across *Plasmodium* species^51,52^, peptides identified in three to seven donors were tested for antigenicity and elicited robust IFNγ responses in samples from *Pv*- and *Pf*-infected patients, as well as in NHP and mouse models of *Plasmodium* infection.

In the Brazilian cohort, T cells from both *Pv*- and *Pf*-infected individuals showed higher responses to the pool of all 50 peptides and to RP-derived peptides, when compared with NE-HD individuals. Enhanced responses to ETRAMP, histone and other peptides were observed only in *Pv-*infected individuals, a finding that is likely to be influenced by the lower prevalence of *Pf* in Brazil, which limited the statistical power in *Pf*-specific analyses. Some previously exposed E-HD individuals also showed peptide-specific responses, indicating persistent T cell memory. Likewise, asymptomatic individuals who controlled parasitaemia without treatment exhibited peptide-specific responses comparable in magnitude with those observed in symptomatic patients, and these data were confirmed in longitudinal NHP cohorts, indicating that these T cell responses are sustained rather than being limited to symptomatic disease.

We also tested peptide-specific responses in *Pf*-infected individuals from Mali and in participants in a CHMI study in the USA, who were infected with blood-stage *Pv*. Both groups had robust antigen-specific T cell responses, confirming the broad applicability of the peptides. Responses from Malian *Pf*-infected individuals show that immunogenicity is independent of previous *Pv* exposure, whereas CHMI data indicate that blood-stage infection alone can induce CD8^+^ T cell immunity, suggesting that blood-stage antigen presentation has a direct role in shaping these responses^3^.

Identical peptides elicited CD8^+^ T cell responses across genetically diverse individuals. Given the high polymorphism of HLA-A, HLA-B and HLA-C alleles in the Brazilian population^53^, one possible explanation for this observation is the ability of certain peptides to bind to several HLA-I molecules^54^. In silico prediction, based on the patient alleles matching their eluted peptides, revealed that *Pv* peptides could bind to multiple HLA-I molecules. A comparison of anti-IFNγ ELISpot results between HLA-I binder and HLA-I non-binder peptides (derived from NetMHC) revealed stronger responses to the predicted non-binder peptides. Because some of these peptides are longer than those usually considered HLA-A, HLA-B and HLA-C ligands^12^, we investigated the antigen-processing machinery of reticulocytes. Our results revealed that reticulocytes are deficient in the expression of key peptide-trimming and loading components, including ERAP1 and tapasin^55^. These deficiencies are likely to favour the presentation of longer, lower-affinity peptides, prompting us to consider a role for epitope restriction by non-classical HLA-I.

HLA-E is the least polymorphic allele of the HLA-I complex. In Brazil, only two HLA-E alleles are expressed by around 90% of individuals, HLA-E*01:01 and HLA-E*01:03 (ref. ^56^). Indeed, functional blocking experiments using VL9 peptide showed that a substantial fraction of CD8^+^ T cell responses to some peptides were restricted to HLA-E (Mamu-E in NHPs). Although the peptides exhibited low or no stability when binding to HLA-E*01:01 or HLA-E*01:03, their ability to activate CD8^+^ T cells underscores the exquisite sensitivity of T cells to antigen. HLA-E-restricted presentation could provide a compelling mechanistic explanation for the broad antigenicity and cross-donor recognition observed in this study.

Most of the peptides found were from housekeeping proteins such as RPs and histones. RPs are expressed throughout the parasite lifecycle^38^ and are highly conserved among *Plasmodium* species. CD8^+^ T cell responses to RP peptides were seen in samples from *Pv*- and *Pf*-infected patients, *Pk*- and *Pcy*-infected NHPs and *Py*-infected mice, providing evidence of cross-species recognition. Previous work identified *Plasmodium* 60S RP L6 (RPL6) as a potent CD8^+^ T cell antigen that provides liver-stage immunity in a rodent model^57^. Our study expands this substantially by identifying other ribosomal antigens—including 60S subunits (L13, L29 and L10) and 40S subunits (S25 and S30)—presented during blood-stage infection that elicit functional CD8^+^ T cell responses in *Pv*- and *Pf-*infected patients, NHPs (during the liver and blood stages) and a mouse model, demonstrating cross-species and cross-stage antigenicity. Collectively, although RPL6 remains a benchmark for liver-stage vaccine design, the newly identified ribosomal antigens expand the landscape of targets with the potential to induce broad-spectrum immunity encompassing both liver and blood stages, supporting their inclusion in next-generation multistage malaria vaccines.

In addition to RPs, we identified ETRAMPs as another class of antigenic proteins. Although ETRAMPs have limited sequence conservation between *Pv* and *Pf*, epitope-specific T cell responses were detected in samples from both infections. Previous studies show that ETRAMPs can confer partial species-specific and cross-species protection, and that their association with another partial protective antigen, *Pf*TRAP, further increases sterile protection^58^. Together, our study highlights ETRAMPs as a potential vaccine target for vivax malaria, suggesting that they could be included in a multivalent vaccine to elicit broad cellular immunity.

Finally, repeated *Pk* sporozoite challenge in NHPs significantly boosted the responses of CD8^+^ T cells to conserved peptides during the blood stage of the infection. Responses to the pool of nine peptides conserved between *Plasmodium* species were particularly boosted, whereas responses to more conventional antigens such as CSP, SSP2 and AMA1 remained relatively unchanged. Liver-directed CVac immunization further enhanced T cell responses to both established antigens (SSP2 and RPL6) and pool-9 antigens, suggesting that CD8^+^ T cells that target these newly identified antigens could contribute to multistage immunity and help overcome limitations associated with the poor boosting of conventional targets.

In summary, this study identifies a new set of *Pv* antigens—especially RPs—naturally presented by HLA-I on infected human reticulocytes. These peptides are highly antigenic in response to *Plasmodium* infection, bind to various HLA-I alleles and strongly activate CD8^+^ T cells. The identified antigens are expressed in the pre-erythrocytic phase of the parasite cell cycle and are highly conserved across *Plasmodium* species. Future studies evaluating vaccine formulations and adjuvants optimized to induce memory, blood- and liver-targeted responses and HLA-E antigen presentation will be crucial for the design of improved malaria vaccines. Such studies will clarify how the conserved ribosomal and non-ribosomal antigens identified here can be harnessed to elicit long-lasting, protective CD8^+^ T cell memory against malaria.

This is the first, to our knowledge, identification of *Plasmodium* antigens and epitopes that are conserved across parasite species and developmental stages. By inducing broad-spectrum, cross-stage and cross-species immunity, our findings provide a conceptual framework for developing next-generation, multistage and potentially universal malaria vaccines.

## Methods

### Malaria-infected patients and healthy donors

*Pv*- and *Pf-*infected patients and endemic healthy donors were recruited in the Tropical Medicine Research Center in Porto Velho, Brazil. Asymptomatic *Pv*-infected donors were identified by active screening and followed up for clinical and parasitaemia evaluation. Single-species infections were confirmed by qPCR. Healthy donors from a non-endemic malarial region were recruited in Belo Horizonte, Brazil. The seven *Pv* samples used in the immunopeptidome workflow were obtained from patients with random HLA types. The samples were confirmed to be infected with *Pv*, not *Pf*, with parasitaemia ranging from 501 to 100,000 parasites per mm^3^. All participants provided written informed consent to participate in the study, which has a protocol approved by the institutional review boards of the Oswaldo Cruz Foundation and the National Ethical Council (CAAE: 59902816.7.0000.5091). For the *Pf*-infected patients from Mali, the clinical samples come from a longitudinal cohort study in Torodo, including 250 individuals aged from 3 months to 16 years. Samples from healthy Malian donors were obtained from people living or working in Torodo. Written informed consent was obtained from all participants or the parents or guardians of participating children. The Ethics Committee of Charité (EA2/264/21), and the Faculty of Medicine, Pharmacy and Odontostomatology (FMPOS) at the University of Bamako, Mali (2022/20/CE/USTTB/24.01.22) approved this study. Clinically relevant information are provided in Supplementary Information 1.

For the CHMI study, healthy, malaria-naive US volunteers aged 18 to 54 were recruited for participation (ClinicalTrials.gov identifier NCT06607003) at the National Institutes of Health (NIH) Clinical Center in Bethesda, Maryland under a protocol approved by the NIH institutional review board (001970). Inclusion criteria included good overall health and positivity for the Duffy antigen receptor. Exclusion criteria included a history of *Pv* malaria or recent travel to an area in which *Pv* is endemic. Volunteers were inoculated with cryopreserved RBCs of the blood-stage *Pv* HMP013 isolate^59^. This method of challenge bypasses the liver stage of infection, exposing participants to blood-stage antigens only. All participants developed PCR-confirmed parasitaemia and early clinical symptoms of malaria. Volunteers participated in mosquito transmission assays before completing curative antimalarial treatment beginning approximately 11 days after inoculation.

### Isolation of PBMCs and *Pv-*infected reticulocytes

One hundred millilitres of blood was collected from infected individuals and control individuals. First, mononuclear cells were separated from peripheral blood. For this, the blood was diluted in a 1:1 ratio and gently added to a tube containing 15 ml of Ficoll (GE Healthcare), and a previously described protocol was followed^60^.

The RBC pellet resulting from PBMC purification was resuspended in RPMI culture medium in a 1:4 ratio. Diluted blood was added to a 50-ml tube containing Percoll 45% (GE Healthcare) at five times the volume of the RBC pellet. Samples were centrifuged at 2,000 rpm for 15 min. After centrifugation, the interface was collected. Purified samples of reticulocytes had 99% purity, identified by Giemsa-stained thin blood smear. All reagents used in this study are listed in Supplementary Information 9.

### HLA-I peptide purification

The detailed protocol for performing immunopeptidomics using mass spectrometry to identify MHC-bound peptides has been previously described^11^. In brief, *Pv-*infected reticulocytes were lysed in 500 µl lysis buffer (1% Igepal, 300 mM NaCl and 100 mM Tris, pH 8.0) supplemented with cOmplete Inhibitor Cocktail (Roche). HLA complexes were immunoprecipitated using 1 mg of W6/32 (a monoclonal antibody against pan-HLA class I complexes; BioLegend) cross-linked to protein A sepharose beads using dimethylpimelimidate (DMP, Sigma). Cell lysates were incubated overnight with W6/32 beads. Beads were subsequently washed with 10 column volumes of 2× 150 mM NaCl in 50 mM Tris, 1× 450 mM NaCl in 50 mM Tris and 50 mM Tris buffer without salt. Peptides bound to the HLA groove were released with 5 ml 10% acetic acid. To separate peptides from the α-chain and β2-microglobulin, samples were further purified by high-performance liquid chromatography (Ultimate 3000, Thermo Fisher Scientific) on a ProSwift RP-1S 4.6 × 50-mm column (Thermo Fisher Scientific) by applying a linear gradient of 2–35% (v/v) acetonitrile in 0.1% (v/v) formic acid in water over 10 min. Samples were dried in a speed vacuum and suspended in 20 μl buffer A (1% acetonitrile and 0.1% trifluoroacetic acid (TFA) in water). Eluted peptide fractions were analysed by nano-ultra-performance liquid chromatography–tandem mass spectrometry (nUPLC–MS/MS).

### nUPLC–MS/MS and data analysis

An UltiMate 3000 RSLCnano system coupled with an Orbitrap Fusion Lumos Tribrid mass spectrometer (Thermo Fisher Scientific) were used to perform mass spectrometry. The mass spectrometry proteomics data have been deposited to the ProteomeXchange Consortium via the PRIDE^61^ partner repository with the dataset identifier PXD077321. Sequence interpretation of MS/MS spectra was performed using a database containing all annotated human UniProt entries (Uniprot UP000005640 (09-2019) and *Pv* UniProt entries (UniProt UP000008333, 08-2019) combined using PEAKS 7 (Bioinformatics Solutions). The Peaks score was used to indicate the statistical significance of the peptide-spectrum match. This value is converted to −10×log_10_(*P*), so a more significant match will have a higher −10logP value. In this study, a Peaks score of 15 was used as the threshold. Peptide length was restricted to 8–15 amino acids to select HLA-I peptides. Sequence homologies between *Pv* peptides and the human proteome were analysed using BLAST+ v.2.16.0. Peptides with at least two amino acids different from the human proteome were included. The same BLAST analyses were performed, comparing peptides found in *Pv* samples to the *Pf* proteome reference strain 3D7 (UP000001450, 08-2019).

### Transcriptome analysis of *Plasmodium* public data

The transcriptome analyses were performed using publicly available databases. Orthologous genes for the 166 *Pv* (Sal-1) proteins identified in the immunopeptidomics analysis were identified using PlasmoD^18^. There were 158 orthologous genes for the *Pv* strain PVP01; 144 for the *Pf* strain Pf3D7; 126 for the *Py* strain PY17X, 129 for the *Plasmodium berghei* (*Pb*) strain PbANKA; 146 for the *Pcy* strain PcyM; and 129 for *Pk *strain PkNH.

Gene-expression data for the species and parasite stages were obtained from PlasmoDB^18^ or published articles. The liver-stage gene expression of *Pf* and *Pv* was obtained from published data^20^. For all stages of *Pf*, two RNA sequencing (RNA-seq) datasets were used^62,63^. Published datasets for *Py*^64^ and *Pk*^65^ were also used. For *Pb*, the analysis was based on two published RNA-seq transcriptomes^66,67^. Two RNA-seq transcriptomes were also used for *Pcy*^68,69^. For RNA-seq analysis, the cut-off used was less than 1 transcript per million (TPM), and for DNA microarrays, the cut-off was a gene without detectable transcript.

### Synthetic peptides

Synthetic peptides were synthesized by GenScript. Peptides were dissolved in dimethyl sulfoxide (DMSO) to 5 mM for ELISpot and spectral match validation.

### ELISpot assay

The ex vivo IFNγ ELISpot assays were performed using 1 × 10^5^–5 × 10^5^ fresh PBMCs per well from *Pv-* and *Pf*-infected patients, endemic healthy donors (E-HD), non-endemic healthy donors (NE-HD) and *Pv* asymptomatic donors. Cells were plated in duplicate into 96-well ELISpot plates (Merck Millipore) precoated with 4 µg ml^−1^ anti-human-IFNγ (clone 1-D1K; Mabtech). A total of 46 peptides were tested at a concentration of 10 µg ml^−1^ with stimulation for 18–20 h at 37 °C, 5% CO_2_. Anti-CD3/anti-CD28 antibodies were used as a positive control and cells cultured with medium only were the negative control (subtracted from all conditions). After cell removal, plates were developed for 2 h in the same temperature condition as the stimulus in the presence of 0.2 µg ml^−1^ anti-IFNγ (7-B6 biotin; Mabtech). Spot detection was performed after incubation for 30 min in the dark with BCIP/NBT alkaline phosphatase substrate (Sigma). SFCs were counted using the ImmunoSpot automated ELISpot counter. Samples were considered positive if they had 30 SFCs or more per million PBMCs.

To assess HLA-E-restricted CD8^+^ T cell responses, CD8^+^ T cells were isolated from PBMCs from *Pv*-infected individuals. The CD8^−^ PBMC fraction, used as antigen-presenting cells, was pulsed overnight with the pooled peptides (10 µg ml^−1^). The following day, antigen-presenting cells were treated with mitomycin C (20 µg ml^−1^) to prevent proliferation and then cocultured with autologous CD8^+^ T cells. Cultures were maintained for approximately 7 days. The medium was supplemented with IL-2 and refreshed every 2 to 3 days. One day before ELISpot analysis, K562 target cells, either lacking HLA class I expression (HLA^−/−^) or overexpressing HLA-E*01:03, were pulsed overnight with the peptide pool. Peptide-pulsed K562 cells were then cocultured with primed CD8^+^ T cells at an effector-to-target ratio of 2:1 and incubated for 24 h in IFNγ ELISpot plates. ELISpot assays were subsequently performed as described above. Both K562 HLA^−/−^ cells and K562 HLA^−/−^ cells overexpressing HLA-E*01:03 were obtained from F. Salusto, and were tested for mycoplasma contamination before use.

### Intracellular staining of PBMCs

Total PBMCs from *Pv* symptomatic patients, *Pv* asymptomatic individuals or healthy donors were stimulated with pools of peptides for the protein class subgroups (40S ribosomal, 60S ribosomal, ETRAMPs, histones, other or a pool of all the 50 peptides). The negative controls was a condition in which no peptide was added, and anti-CD3/anti-CD28 monoclonal antibodies were used as positive controls. After overnight incubation, brefeldin and monensin were added for 4 h. The cells were collected and stained with the viability dye Zombie Yellow (BioLegend), then stained with anti-CD3, anti-CD4, anti-CD8 and anti-CD69 antibodies. Cells were fixed and permeabilized with Cytofix/Cytoperm (BD Biosciences) and stained with anti-IFNγ or anti-IL-2 antibody. For assessing CD107a degranulation, the anti-CD107a antibody was added at the start of peptide stimulation, and brefeldin A and monensin were not included in the culture. The samples were acquired on a FACSCelesta with FACSDiva software v.8.0 (BD Biosciences), and subsequent analysis was performed using FlowJo v.10 (Tree Star). Representative gating strategies are shown (Supplementary Information 10). For HLA-blocking assays, total PBMCs from *Pv*-infected patients were blocked with an anti-HLA-I antibody (W6/32) or a VL9 peptide (for HLA-E blocking) for 30 min. Isotype antibody or TH9 peptide was used as the blocking control, respectively. Next, the pool with all 50 peptides was added to the PBMC culture for overnight stimulation, and intracellular staining was performed as described above. The negative control was a condition in which no peptide was added, and anti-CD3/anti-CD28 monoclonal antibodies were used as positive controls.

### Spectral match validation

A spectral match validation experiment was performed to confirm that the peptide sequences were assigned correctly. Fifty synthetic peptides were selected, produced and compared with the original identified sequences, referred to as the biological peptides. Synthetic peptides were run in the same experimental conditions as biological peptides. The parameters used to confirm the sequence identity were the mass-to-charge ratio (*m*/*z*) for each peptide; the charge number of each peptide (*z*); the intensity and distribution of each peak within each peptide sequence; and the peptide-specific retention time. These were compared between synthetic and biological peptides, and the sequences matched (Supplementary Information 6).

### HLA class I genotyping

To determine the typing of HLA-A, HLA-B and HLA-C alleles from the samples used in the immunopeptidomics assay, the total genomic DNA was extracted from 5 × 10^6^ PBMCs using the QIAamp DNA Mini Kit (QIAGEN) according to the manufacturer’s instructions. HLA four-digit typing was performed by Sanger sequencing on a 48-capillary ABI-3730 DNA analyser.

### Prediction of HLA-A, HLA-B and HLA-C binders

For data curation, the binding affinity of all HLA-I eluted peptides was analysed using NetMHCpan-4.0^23^ locally. Predictions were performed for each sample according to its HLA-A, HLA-B and HLA-C types. The best HLA-binding allele and the best score were identified for all *Pv* peptides. Peptides with a threshold below 10% were considered as ligands.

### Cell-based HLA class I peptide stability assay

Concentration-based classical and non-classical HLA class I peptide stability assays were performed as previously described^32^. In brief, 5 × 10^4^ TAP-deficient mono-allelic HLA-A*02:01-, HLA-E*01:01- or HLA-E*01:03-expressing 721.221 cells were incubated with 100 μM peptides in RPMI 1640 medium in a 96-well plate overnight at 26 °C, 5% CO_2_ for 18 h. A DMSO-only control and a positive control with known HLA binders were performed in parallel, then subtracted from all sample results. After overnight incubation, cells were incubated at 37 °C, 5% CO_2_ before staining for viability and HLA class I surface expression with anti-HLA-ABC (W6/32) or anti-HLA-E (3D12) antibodies (1:100), and were subsequently analysed by flow cytometry. The 721.221 cell lines were originally obtained from ATCC (CRL-1855). HLA-expressing cells were generated in the laboratory of G. D. Gaiha^32^. Cells were tested for mycoplasma contamination before experimental use.

### Immunoblotting

Cell lysates of infected and uninfected reticulocytes, obtained by lysis in radioimmunoprecipitation assay buffer (Sigma) in the presence of complete protease inhibitor (Roche), were analysed by immunoblots probed for both tapasin and ERAP1 (Abcam), after the removal of haemoglobin using HemogloBind (Biotech Support Group). Anti-β-actin and anti-F-actin antibodies (Abcam) were used as loading controls for uninfected and infected cells, respectively^3^. Each lane of reticulocytes was loaded with 50 μg of protein, whereas the PBMC control sample contained 20 μg of protein. Two mirrored 10% SDS–PAGE gels and one 12% SDS–PAGE gel were run simultaneously with equivalent sample lysates. The gels were simultaneously transferred to nitrocellulose membranes for respective blotting. Anti-β-actin blotting was performed after stripping the membrane, after anti-tapasin blotting. The secondary anti-mouse or anti-rabbit immunoglobulin G antibody was detected by chemiluminescence.

### HLA-E staining

Total RBCs from healthy donors or *Pv*-infected individuals were stained with SYBR green (parasite nuclei), anti-CD235a–APC (RBC marker), anti-CD71–PE (reticulocyte marker) and anti-HLA-E–PE–Cy7 (3D12), then analysed by flow cytometry. HLA-E expression was evaluated for the uninfected RBC population (CD235a^+^CD71^−^SYBR^−^), uninfected reticulocytes (CD235a^+^CD71^+^SYBR^−^) and infected reticulocytes (CD235a^+^CD71^+^SYBR^+^), in comparison with total RBCs stained with isotype APC.

### *Pv* and *Pf* liver-stage gene expression

To verify whether the proteins identified in the *Pv* blood stage immunopeptidome are also expressed in the *Pf* and *Pv* liver stages, liver samples were taken from humanized mice infected with the *Pf* strain NF135. All procedures involving humanized mice were reviewed and approved by the Institutional Animal Care and Use Committee at Oregon Health and Science University (IACUC protocol number: IP00002077). Mice were infected as previously described^70,71^. The highly infective strain *Pf*NF135 was produced and supplied by R. Sauerwein^72^. The *Pv* sporozoites were from a Thai field isolate provided by A. Vaughan. Five genes were quantified through RT–qPCR: 40S RP S25, 40S RP S30, 60S RP L30, 60S RP L13 and histone H2A. In addition, two genes known to be expressed during the liver stage, CSP and TRAP, were used as positive controls.

For *Pf*, livers from mice intravenously infected with five million sporozoites were collected five days after infection. For RNA extraction, the RNeasy Plus Mini Kit (QIAGEN) was used according to the manufacturer’s protocol. For the conversion of RNA into complementary DNA (cDNA), 1 µg of RNA was used, and the SuperScript IV First-Strand Synthesis System (Invitrogen) protocol was followed. SYBR green was used as a fluorescent DNA intercalant with 5 µl cDNA and 300 nM of each primer added to the reaction (Supplementary Information 9). Tests were performed on the StepOne Real-Time PCR System (Applied Biosystems) thermal cycler under the following cycling conditions: 1 cycle at 50 °C for 2 min, 1 cycle at 95 °C for 2 min, followed by 40 cycles of 95 °C for 15 s and 60 °C for 60 s.

For *Pv*, the methods followed the same general protocol with the following exceptions. Mice were intravenously infected with 400,000 sporozoites, and livers were collected 9 days after infection. PowerTrack SYBR Green Master Mix (Applied Biosystems) was used for qPCR, with 2 µl cDNA and 400 nM of each primer added, using unique primers designed for *Pv* (Supplementary Information 9). The thermal cycling programme began with a hold stage at 95.0 °C for 2 min, followed by 40 amplification cycles consisting of denaturation at 95.0 °C for 15 s and annealing optimized according to the gene for 1 min.

Samples were normalized by calculating ΔCt (Ct of the target gene − Ct of the endogenous gene). *Plasmodium* 18S rRNA was used as a normalizer. For relative quantification of gene expression, the 2^−∆Ct^ calculation was performed.

### NHP studies

Indian-origin rhesus macaques were obtained from and housed at the Oregon National Primate Research Center (ONPRC) for these studies. Macaques were housed in pairs with a suitable partner whenever possible and had visual, auditory and olfactory contact with others. Rhesus macaques received commercially prepared primate chow twice daily and received supplemental fresh fruit or vegetables daily. Fresh, potable water was provided by automatic water systems. Physical exams, including body weight and complete blood counts, were performed at scheduled protocol time points. Macaques were sedated with ketamine HCl with the addition of dexmedetomidine and atipamezole as a reversal agent for some procedures, including some blood draws and intravenous parasite challenge. For parasitaemia assessment, capillary blood samples were obtained cage-side by standardized ear-prick procedures, or were collected in EDTA-coated plastic vacutainer tubes. All experimental protocols and procedures were approved by the IACUC at Oregon Health and Science University under protocols IP00003877, IP00001114 and IP00001169. ONPRC is a category I facility, and the Laboratory Animal Care and Use Program at the ONPRC is fully accredited by the American Association for Accreditation of Laboratory Animal Care (AAALAC) and has an approved assurance (A3304-01) for the care and use of animals on file with the NIH Office for Laboratory Animal Welfare. The IACUC adheres to national guidelines established in the Animal Welfare Act (7 USC 2131–2159) and the Guide for the Care and Use of Laboratory Animals (8th edition).

### Infection and immunization of NHPs with *Plasmodium* parasites

In studies of *Pk* infection, control macaques from unrelated rhesus cytomegalovirus (CMV) vaccine studies were used. These macaques were either completely naive (*n* = 3) or immunized with non-malarial vaccine vectors (*n* = 4). Each infection was initiated by the intravenous injection of 2,500 purified, aseptic, cryopreserved sporozoites (obtained from Sanaria). Cryopreserved sporozoites were thawed in a 37 °C water bath for 30 s and resuspended in sterile PBS containing 1% human serum albumin to 1 ml per animal. Sporozoites were injected within 30 min of thaw. Parasitaemia was monitored daily by Giemsa-stained thin smears from day 6 onwards, until it reached 2%, when treatment with artesunate (8 mg kg^−1^ intramuscularly, once) plus chloroquine (15 mg kg^−1^ once daily for three days) was initiated. Parasitaemia was monitored daily until cleared and twice weekly for four weeks to ensure treatment efficacy.

For studies of *Pk* immunization by CVac, macaques were injected intravenously with 50,000 sporozoites per animal per dose (purified, aseptic, cryopreserved sporozoites were obtained from Sanaria). Cryopreserved sporozoites were thawed in a 37 °C water bath for 30 s and resuspended in sterile PBS containing 1% human serum albumin to 1 ml per animal. Sporozoites were injected within 30 min of thaw. During the immunization period, macaques were administered a 10 mg kg^−1^ loading dose of chloroquine followed by 5 mg kg^−1^ weekly beginning the week of immunization. In addition, macaques were administered one tablet of Coartem twice a day (morning and evening) on days 6, 7 and 8 after each immunizing inoculation. Parasitaemia was monitored by Giemsa-stained thin smears on days 7, 9 and 12 after each immunization, with weekly monitoring thereafter. Blood was also collected in EDTA and in nucliSENS lysis buffer (Biomerieux, 200292) for qRT–PCR assessment of parasitaemia.

For *Pcy* studies, macaques were infected with cryopreserved RBCs from the *Pcy* Berok strain by intravenous injection. Glycerolyte-preserved stocks were thawed in a 37 °C water bath, and a 0.2× volume of sterile 12% saline solution was added dropwise with mixing. After a 5-min incubation at room temperature, 12 volumes of 1.6% saline were added dropwise while mixing. Cells were then pelleted at 800*g* for 4 min, the supernatant was removed and the pellet was washed twice with 10 ml of 0.9% saline, adding the saline dropwise while mixing. After the final wash, the supernatant was removed, and the cells were resuspended in 1 ml RPMI per animal for injection. Parasitaemia was monitored in peripheral blood by Giemsa-stained thin smears after ear prick or sedated blood draw into EDTA.

### Laparoscopic biopsy

Macaques were fasted overnight and sedated with an intramuscular injection of 10 mg kg^−1^ ketamine HCl (Ketathesia, Henry Schein Animal Health) with 0.015 mg kg^−1^ dexmedetomidine hydrochloride (Dexmedesed, Dechra). After sedation, macaques were intubated and placed on isoflurane (0.8–2%). All macaques received 0.2 mg kg^−1^ sustained-release buprenorphine (Buprenorphine SR, ZooPharm) subcutaneously for postoperative analgesia.

Once the macaques had been prepared for surgery, three incisions were made to allow the insertion of a 5-mm camera port just caudal to the umbilicus and two 5-mm midabdominal instrument ports (right and left). A 5-mm Caiman vessel sealer (Aesculap) was used to perform a partial lobectomy (5–15 g) of the left medial or left lateral liver lobe. Incisions were closed with 4-0 monofilament absorbable sutures, and a local block with bupivacaine was performed. After the procedure, sedation was reversed with atipamezole hydrochloride (Antisedan, Zoetis, Kalamazoo) and the macaques were extubated and allowed to recover. For liver pinch biopsies, the procedure was performed as described^73^.

### ICS

For T cell analysis, peripheral blood was collected in ACD anti-coagulant, and PBMCs were isolated using a Percoll gradient. For isolation of liver lymphocytes, biopsies (4–20 g) were stored in cold Servator B (Global Transplant Solutions) before isolation. The tissue was processed as soon as possible after surgical removal (within 2 h). Tissue was weighed and rinsed in base buffer (Hanks’ balanced salt solution (HBSS), 1.5% fetal bovine serum (FBS), 2 mM CaCl_2_ and 5 mM HEPES), followed by vigorous mechanical dissociation into a minced slurry using a vegetable chopper. Dissociated tissue was transferred to a 125-ml vented Erlenmeyer flask containing 25 μg ml^−1^ liberase TL (Roche) in base buffer (75 ml liberase buffer per 15 g tissue). The flask was agitated at 250 rpm for 30 min at 37 °C, and the digested tissue was strained through a 70-μm cell strainer (Falcon). The resulting liquid suspension was centrifuged at 50*g* for 2 min to remove hepatocytes. The supernatant was pelleted at 350*g* for 5 min, and the pellet was resuspended in ACK buffer (KD Medical) to remove RBCs. Cells were washed, pelleted by centrifugation and resuspended in 10 ml 20% OptiPrep (iodixanol, Sigma) overlaid with 10 ml PBS containing 2% FBS. NPCs were isolated from the gradient interface after centrifugation at 1,500*g* for 25 min without brake.

Antigen-specific CD8^+^ T cell responses were measured by ICS^74–76^. In brief, PBMCs were incubated with peptides for 1 h, followed by the addition of brefeldin A (Sigma-Aldrich) for a further 8 h. Incubation without peptides served as the background control. Stimulated cells were fixed, permeabilized and stained using combinations of the following fluorochrome-conjugated monoclonal antibodies: SP34-2 (CD3; Pacific Blue, Alexa700), L200 (CD4; AmCyan, BV510), SK-1 (CD8; PerCP–Cy5.5), MAB11 (TNF; FITC, PE), B27 (IFNγ; APC) and FN50 (CD69; PE–Texas Red). Data were collected on an LSR-II with FACSDiva v.8.0 (BD Biosciences), and subsequent analysis was performed using FlowJo (Tree Star). Lymphocytes were gated for CD3^+^ and then further gated for CD4^−^ and CD8^+^ T cells. Antigen-responding cells in the CD8^+^ T cell subset were identified by their co-expression of one or more cytokines with CD69. Gating strategies are shown in Supplementary Information 10.

### Rodent model of *Py* malaria

For the mouse study, procedures were performed in accordance with the National Council for the Control of Animal Experimentation (CONCEA). The protocols were approved by the Oswaldo Cruz Foundation (FIOCRUZ) Animal Experimentation Council (CEUA protocol LW-2/21).

C57BL/6 female mice, 8–10 weeks old, were purchased and maintained at the FIOCRUZ Minas animal facility, under a 12-h light–dark cycle, relative humidity of around 50% and temperature of 20–25 °C. Mice were infected with *Py* 17XNL PYGFP, a GFP-expressing strain purchased from MR4 at ATCC. Cryopreserved parasites were thawed and passaged in vivo twice before being used in experimental infections. A non-lethal inoculum of 10^5^ infected RBCs was administered intraperitoneally.

### Mouse IFNγ ELISpot assay

To validate peptides in a mouse model, mice were inoculated with 10^5^ RBCs infected with a non-lethal strain of *Py* that preferentially infects reticulocytes^77^. Twelve days after infection, splenocytes from mice were collected for the ex vivo IFNγ ELISpot assay. A total of 10^6^ cells were plated in duplicates on 96-well ELISpot plates (Merck Millipore) precoated with 5 µg ml^−1^ anti-mouse-IFNγ (clone 1-D1K; Mabtech). Fifty peptides were tested at a concentration of 10 µg ml^−1^ and cells were stimulated for 20 h at 37 °C, 5% CO_2_. PMA (1 µg ml^−1^) and ionomycin (100 µg ml^−1^) were used as positive controls, and the medium alone as a negative control. After cell removal, plates were developed for 2 h in the same temperature conditions that were used for the stimulus, in the presence of 0.2 µg ml^−1^ anti-IFNγ (7-B6 biotin; Mabtech). SFCs were counted using the ImmunoSpot automated ELISpot counter. The results are expressed as SFCs per 10^6^ splenocytes. Peptides were considered positive when the group mean was 7 SFCs or more per 10^6^ splenocytes. The cut-off was established using the response of uninfected mice to each peptide, with the value calculated on the basis of average response ≥ 0 plus three times the standard deviation.

### Generation of epitope-specific cytotoxic T cells

The procedure used to generate epitope-specific cytotoxic T lymphocytes (CTLs) was adapted from that of previous studies^78,79^. Mice were infected with 10^5^ *Py*-infected RBCs, at the peak of parasitaemia (30–40% infected RBCs). The spleen was collected, and splenocytes were seeded at 1 × 10^7^ cells in a 24-well culture plate in 1.5 ml RPMI 1640 supplemented with 10% FBS (complete RPMI; Sigma) plus 1 μg ml^−1^ of each peptide. Twenty-four hours later, 100 U ml^−1^ of recombinant IL-2 (BioLegend) in 0.5 ml complete RPMI was added to the culture. Restimulation occurred every 12 days by 1:1 split of the cultures by supplying 1×10^6^ peptide-pulsed and Mitomycin-treated splenocytes from uninfected mice, as stimulator cells^80^. One day later, 100 U ml^−1^ of IL-2 was added to 0.5 ml of complete RPMI. Between restimulations, cultures were fed fresh medium containing 50 U ml^−1^ IL-2 as needed.

### Analysing cytokine production in mouse cells by ICS

Twelve hours after the second stimulation, 1 × 10^6^ cells were collected and cultured with brefeldin A (Sigma-Aldrich) for 6 h. Incubation without peptides served as the background control. Cultures were washed twice with PBS and stained with Zombie Yellow (BioLegend). Cells were permeabilized and fixed using the Fixation/Permeabilization Kit (BD) and stained using the following anti-mouse fluorochrome-conjugated monoclonal antibodies: 17A2 (CD3; APC), 53-6.7 (CD8; PerCP–Cy5.5), XMG1.2 (IFNγ; Pacific Blue) and MP6-XT22 (TNF; PE–Cy7) from BioLegend. Data were collected on an LSR-II (BD Biosciences), and subsequent analysis was performed using FlowJo v.10 (Tree Star). Lymphocytes were gated for Zombie-negative cells, then CD3^+^ and CD8^+^ T cells were gated. Gating strategies are provided in Supplementary Information 10. Antigen-responding cells in the CD8^+^ T cell subset were identified by their expression of IFNγ and TNF. Statistical comparisons with the unstimulated group were done using a two-way ANOVA.

### Antigen-specific CTL killing assay

The killing assay was performed using a previously described methodology^43^. In brief, 12 days after the second stimulation (see ‘Generation of epitope-specific cytotoxic T cells’), antigen-specific CTLs were purified using the CD8a^+^ T Cell Isolation Kit II (130-095-236; Miltenyi). *Py*-infected RBCs were isolated by magnetic separation using LS columns (130-042-401; Miltenyi) and then labelled with the CellTrace CFSE Cell Proliferation Kit (Invitrogen). The purified CTLs and labelled infected RBCs were cocultured for 4 h at 37 °C, 5% CO_2_, followed by cell-specific staining using the anti-mouse monoclonal antibodies 53-6.7 (CD8a; PerCP–Cy5.5) and TER-119 (erythroid cells; APC–Cy7) from BioLegend. Precision count beads (BioLegend) were added to set the stop gating. Data were collected on an LSRFortessa Cell Analyzer (BD Biosciences), and subsequent analysis was performed using FlowJo.

### Adoptive transfer of peptide-specific CD8^+^ T cells in a mouse model of *Py* infection

To perform the adoptive transfer, 12 days after the second stimulation (see ‘Generation of epitope-specific cytotoxic T cells’), antigen-specific CTL cells were purified using the CD8a^+^ T Cell Isolation Kit II (Miltenyi). Mice received 1.5 × 10^6^ peptide-specific CTLs on day 0, before challenge, and on day 4 after infection. Mice were challenged with 10^5^ *Py*-infected RBCs, and parasitaemia was assessed for 28 days by flow-cytometry analysis. To monitor parasitaemia, 3 μl of a tail-blood sample was collected, diluted in 20 μl PBS and heparin (Cristalia), then diluted 1:10 in FACS buffer. Samples were collected on an LSRFortessa Cell Analyzer (BD Biosciences) until 100,000 RBCs were acquired. Infected RBCs (GFP^+^) were gated on the basis of GFP expression by *Py* parasites, and parasitaemia was analysed using FlowJo.

At end-point assessment, splenocytes were collected and stained for cell activation and cytokine production. In brief, 1 × 10^6^ cells were incubated with 10 μg ml^−1^ of specific peptide for 12 h, followed by incubation with brefeldin A for 6 h. Cells were then stained as described above using Zombie Yellow (BioLegend), a fixation/permeabilization kit (BD) and the following anti-mouse monoclonal antibodies: 17A2 (CD3; Pacific Blue), 53-6.7 (CD8; Alexa Fluor 700), GK1.5 (CD4; FITC), H1.2F3 (CD69; PE), XMG1.2 (IFNγ; APC) and MP6-XT22 (TNF; PE–Cy7) from BioLegend.

### Immunization of mice with *Pv* RPs

Mice (*n* = 10) were immunized three times by subcutaneous injection with 100 µl of a solution composed of 10 μg of the target protein plus adjuvant solution^81^ (18 µg of B344 and 30% (v/v) of alum rehydragel LV solution), at 14-day intervals. The adjuvant group received only the adjuvant solution. Fourteen days after the last immunization, blood samples and spleens were collected from a subset of mice (*n* = 4) to evaluate immune responses. Cells were stimulated with 10 μg ml^−1^ of each recombinant protein, and the ICS protocol was performed as described in the previous section. Finally, 21 days after the last boost, mice (*n* = 6) were challenged with 10^4^ *Py*-infected RBCs, and parasitaemia was monitored for 28 days, as previously described.

### Graph design and statistical analysis

Graph designs were generated using GraphPad Prism v.10.4.2 or R v.4.4.1. Statistical analyses were performed using GraphPad Prism v.10.4.2. The normality of the data was evaluated using D’Agostino and Pearson normality tests before statistical methods were applied. Distributions were considered normal if *P* ≤ 0.05. Two columns fitting the normality test were compared using two-tailed paired or unpaired *t*-tests. Non-parametric data were compared by the Mann–Whitney test. Multiple columns comparisons were analysed by one-way ANOVA followed by Sidak’s or Tukey’s multiple comparisons test, or by non-parametric Kruskal–Wallis test with Dunn’s post-hoc test. Multiple groups were compared by two-way ANOVA followed by Sidak’s, Dunnett’s or Tukey’s multiple comparisons test.

No statistical methods were used to determine the sample size. The experiments were not randomized, and the investigators were not blinded to allocation during the experiments or to the outcome assessment.

### Data reporting

No statistical methods were used to determine sample size. The experiments were not randomized, and the investigators were not blinded to allocation during the experiments or to the outcome assessment.

### Reporting summary

Further information on research design is available in the Nature Portfolio Reporting Summary linked to this article.

## Online content

Any methods, additional references, Nature Portfolio reporting summaries, source data, extended data, supplementary information, acknowledgements, peer review information; details of author contributions and competing interests; and statements of data and code availability are available at https://doi.org/10.1038/s41586-026-10730-1.

## Supplementary information

Supplementary Information Supplementary Information files 1-10

Reporting Summary

Peer Review file

## Source data

Source Data Figs. 3 and 4

Source Data Extended Data Fig. 5

## Data Availability

The data that support the findings of this study are available in the Article and its Supplementary Information. The mass spectrometry proteomics data are available via ProteomeXchange with the identifier PXD077321. Source data are provided with this paper.
