# Supplementary material for: Identification of cross-stage, cross-species malaria CD8+ T cell antigens
Source: Nature. 2026 Jul 1;655(8123):747–57. doi: 10.1038/s41586-026-10730-1 (PMC13661247; doi:10.1038/s41586-026-10730-1)
Supplement: Supplementary file 1 [file 41586_2026_10730_MOESM2_ESM.pdf]

nature portfolio

Corresponding author(s): Caroline Junqueira

Last updated by author(s): May 4, 2026

Reporting Summary

Nature Portfolio wishes to improve the reproducibility of the work that we publish. This form provides structure for consistency and transparency in reporting. For further information on Nature Portfolio policies, see our Editorial Policies and the Editorial Policy Checklist.

Statistics

For all statistical analyses, confirm that the following items are present in the figure legend, table legend, main text, or Methods section.

n/a	Confirmed	
☐	☒	The exact sample size (*n*) for each experimental group/condition, given as a discrete number and unit of measurement
☐	☒	A statement on whether measurements were taken from distinct samples or whether the same sample was measured repeatedly
☐	☒	The statistical test(s) used AND whether they are one- or two-sided<br>*Only common tests should be described solely by name; describe more complex techniques in the Methods section.*
☒	☐	A description of all covariates tested
☐	☒	A description of any assumptions or corrections, such as tests of normality and adjustment for multiple comparisons
☐	☒	A full description of the statistical parameters including central tendency (e.g. means) or other basic estimates (e.g. regression coefficient) AND variation (e.g. standard deviation) or associated estimates of uncertainty (e.g. confidence intervals)
☐	☒	For null hypothesis testing, the test statistic (e.g. *F*, *t*, *r*) with confidence intervals, effect sizes, degrees of freedom and *P* value noted<br>*Give P values as exact values whenever suitable.*
☒	☐	For Bayesian analysis, information on the choice of priors and Markov chain Monte Carlo settings
☒	☐	For hierarchical and complex designs, identification of the appropriate level for tests and full reporting of outcomes
☒	☐	Estimates of effect sizes (e.g. Cohen's *d*, Pearson's *r*), indicating how they were calculated

Our web collection on statistics for biologists contains articles on many of the points above.

Software and code

Policy information about availability of computer code

Data collection	No software was used for data collection.
Data analysis	Immunopeptidome peptide sequence interpretation were performed using a database containing all annotated human Uniprot entries (Uniprot- UP000005640 -09-2019) and P. vivax Uniprot entries (Uniprot- UP000008333- Version 08-2019) combined using PEAKS 7 (Bioinformatics Solutions Inc).<br><br>Peptide binding to HLA class I was predicted by NetMHCpan-4.0.<br><br>Sequence homologies between Pv peptides and the human proteome were analysed on BLAST+ v. 2.16.0.<br><br>Transcriptome analyses were performed based on available open databases deposited in PlasmoDB (release 68)or as supplementary information in published articles. For P. vivax strain PVP01 (GCA_900093555); P. f strain Pf3D7 (GCA_000002765.6); Py strain PY17X (GCA_900002385.2), Pb strain PbANKA (GCA_900002375.2), PcyM (GCA_900180395.1), PkNH (GCA_000006355.3).<br><br>Graph design and statistical analysis were performed using GraphPad Prism v. 10.4.2 and R-4.4.1.<br><br>Flow cytometry data were aquired with FACSDiva v 8.0 (BD Biosciene) and analyzed by FlowJo v. 10.10 (Tree Star).

For manuscripts utilizing custom algorithms or software that are central to the research but not yet described in published literature, software must be made available to editors and reviewers. We strongly encourage code deposition in a community repository (e.g. GitHub). See the Nature Portfolio guidelines for submitting code & software for further information.

Data

Policy information about availability of data

All manuscripts must include a data availability statement. This statement should provide the following information, where applicable:

- Accession codes, unique identifiers, or web links for publicly available datasets
- A description of any restrictions on data availability
- For clinical datasets or third party data, please ensure that the statement adheres to our policy

The data that support the findings of this study are presented in the manuscript as figures, extended data, supplementary information and source data. The mass spectrometry proteomics data have been deposited to the ProteomeXchange Consortium via the PRIDEpartner repository with the dataset identifier PXD077321.

Research involving human participants, their data, or biological material

Policy information about studies with human participants or human data. See also policy information about sex, gender (identity/presentation), and sexual orientation and race, ethnicity and racism.

Reporting on sex and gender	All Plasmodium-infected individuals were enrolled without sex selection, aiming at include as many patients as possible. Non-endemic and endemic healthy donor were enrolled to match sex and age of the malaria patients.
Reporting on race, ethnicity, or other socially relevant groupings	Brazilian population is extremely diverse and mixed. The enrolled subjects, from all groups fell into mixed race. Socially aspects were not considered.
Population characteristics	Patients infected with P. vivax or P. falciparum and endemic healthy donors living in an endemic area of malaria in Porto Velho (Brazil) were included in our study. Both male and female individuals ranging from 18 to 65 years old, as approved by the ethical committee.<br>Patients infected with P. falciparum and endemic healthy donors living in an endemic area of malaria in or Torodo (Mali) were included in our study. Both male and female individuals ranging from 3 months t1o 6 years old, as approved by the ethical committee.<br>Infected and healthy donors should not have had malaria therapy in the past 6 months. Individuals co-infected with both Pf and Pv, pregnant women, people receiving treatment with immunosuppressive drugs, and individuals with infectious or chronic inflammatory diseases were excluded.
Recruitment	P. vivax and P. falciparum-infected patients with positive blood smears (greater than 501 parasites/mm^3) and positive PCR assays were invited to enroll in the study. After obtaining informed consent, 100 ml of blood was collected.<br>Inclusion criteria for symptomatic malaria included documented pyrexia (T> 38 ° C) or frank history of chills within 48 h before recruitment and two diagnostic tests (thick smear and PCR). All individuals who tested positive for Plasmodium were invited to participate in the study, and there was no bias in inclusion.<br>P. vivax-infected asymptomatic individuals were identified by active search in the great area of Porto Velho, Rondonia, Brazil.. To ensure the absence of symptoms, individuals were monitored for 3 weeks. All individuals who tested positive for Plasmodium and had no symptoms were invited to participate in the study, and there was no bias in inclusion. Those individuals who became symptomatic within two weeks were excluded from the study.<br>The project was developed in collaboration with researchers and physicians from the Research Center for Tropical Medicine of Rondonia (CEPEM) and FIOCRUZ Rondonia, who were in charge of enrolling patients into our study.<br><br>Individuals working at CEPEM and FIOCRUZ Rondonia were invited to participate as endemic healthy donors. All individuals were tested for Plasmodium infection by PCR.<br><br>Non-endemic healthy donor blood samples were obtained from individuals living in Belo Horizonte, Brazil, a non-endemic region for Malaria. Individuals working at FIOCRUZ Minas were invited to participate as endemic healthy donors. All individuals were tested for Plasmodium infection by PCR.<br><br>For the Pf-infected patients from Mali, the clinical samples come from a longitudinal cohort study in Torodo including 250 individuals in the age of 3 months to 16 years. Written informed consent was obtained from all participants or the parents/guardians of participating children.<br><br>For the Controlled Human Malaria Infection (CHMI) study, Healthy, malaria-naïve US volunteers aged 18 to 54 were recruited for participation in the study at the National Institutes of Health (NIH) Clinical Center in Bethesda, Maryland, USA Inclusion criteria included good overall health and positivity for the Duffy antigen receptor. Exclusion criteria included history of Pv malaria or recent travel to a Pv endemic area. Volunteers were inoculated with cryopreserved red blood cells of the blood-stage Pv HMP013 isolate. This method of challenge bypasses the liver stage of infection, exposing participants to only blood stage antigens. All developed PCR-confirmed parasitemia and early clinical malaria symptoms. Volunteers participated in mosquito transmission assays before completing curative antimalarial treatment beginning approximately 11 days after inoculation.
Ethics oversight	All participants provided written informed consent for participation in the study. Brazilian study protocol approved by the Institutional Review Boards of the Oswaldo Cruz Foundation and National Ethical Council (CAAE: 59902816.7.0000.5091).<br><br>Malian study protocol was aproved by The Ethics Committee of Charité (EA2/264/21), and the Faculty of Medicine, Pharmacy

and Odontostomatology (FMPOS) at the University of Bamako, Mali (N°2022/20/CE/USTTB/24.01.22) approved this study.

CHMI study (ClinicalTrials.gov identifier NCT06607003) was performed under a protocol approved by the NIH Institutional Review Board (#001970).

Note that full information on the approval of the study protocol must also be provided in the manuscript.

Field-specific reporting

Please select the one below that is the best fit for your research. If you are not sure, read the appropriate sections before making your selection.

☒ Life sciences ☐ Behavioural & social sciences ☐ Ecological, evolutionary & environmental sciences

For a reference copy of the document with all sections, see nature.com/documents/nr-reporting-summary-flat.pdf

Life sciences study design

All studies must disclose on these points even when the disclosure is negative.

Sample size

Our study planned to enroll 30 individuals for Plasmodium vivax-infected reticulocytes (iRetics) immunopeptidome, as we already anticipated a technical difficulty in obtaining the number of iRetics needed to proceed with the immunopeptimic workflow. From the 30 enrolled patients, we were able to isolate the minimum number of iRetics from 7 patients. For this sample collection we did not anticipate a N size, since obtaining enough cells is a methodological challenge.

For the in vitro validation of the identified peptides, we anticipated a N=50 for P. vivax and P. falciparum and a N=30 for endemic and non-endemic healthy donors. At the conclusion of the study, were able to enroll only 50 P. vivax patients, 7 P. falciparum from Brazil, 5 P. falciparum from Mali, 30 endemic healthy donors from Brazil, 7 endemic healthy donors from Mali, and 20 non-endemic healthy donors. The sample size above was established for ex vivo analysis, presented in figure 1. For each ex vivo characterization, the sample size had a minimum of 5 subjects in order to obtain significance. The N employed in this study was based on our research group previous experience with immunological assesement of Plasmodium infected individuals in endemic areas for malaria. Although we anticipated a higher N, we N here reported is enouth to obtain statistic significance and to suport our conclusion, as demonstrated

For the Controlled Human Malaria Infection (CHMI) study of P. vivax blood stage forms, it was recruited 6 healthy volunteers, who were evaluated before and after the infection.

For non-human primates and mouse studies we chose sample sizes based on pilot experiments and literature reports to achieve at least 80% power and a two-sided type I error of 5%. The chosen sample sizes were sufficient to determine statistical significance in our established human data and animal models data.

For humanized mouse data 3 mice were infected with P. falciparum, and 4 mice were infected with P. vivax.

For rodent mouse studies, a minimum of N=3 of C57BL/6 mice were employed for in vitro stimulation assays and cytokines assessment. Parasitemia curves were evaluated with a minimum of N=4 mice/ group.

Data exclusions

No data were excluded from our analysis.

Replication

All in vitro and ex vivo assays employing human and non-human primates samples were performed in duplicates for each individual. All the attempts at replication were successful.

The mouse model data presented were successful in all 3 attempts or in the case of one failure, it was performed a fourth time to confirm the results to support conclusions stated in the manuscript.

Randomization

The inclusion of participants in our study was completely randomized. Subjects were assigned as they arrived at the Research Center in Brazil and in Mali. Groups (non-endemic healthy donors, endemic healthy donor, Plasmodium vivax and P. falciparum) were solely based on their infectious status.

CHMI study enrolled malaria naïve individuals without any co-morbidity.

Animals were assigned randomly to experimental and control groups. Random allocation and quantitative measurement using instruments and kits in our experiments minimized biased assessments.

Blinding

Blinding was not possible for the all of the experiments, since for each assay performed, the same scientist was responsible to prepare, acquire and analyze.

Reporting for specific materials, systems and methods

We require information from authors about some types of materials, experimental systems and methods used in many studies. Here, indicate whether each material, system or method listed is relevant to your study. If you are not sure if a list item applies to your research, read the appropriate section before selecting a response.

Materials & experimental systems

n/a	Involved in the study	
☐	☒	Antibodies
☐	☒	Eukaryotic cell lines
☒	☐	Palaeontology and archaeology
☐	☒	Animals and other organisms
☒	☐	Clinical data
☒	☐	Dual use research of concern
☒	☐	Plants

Methods

n/a	Involved in the study	
☒	☐	ChIP-seq
☐	☒	Flow cytometry
☒	☐	MRI-based neuroimaging

Antibodies

Antibodies used

Antibodies Source Identifier (clone, Cat. #)

Flow Cytometry
Alexa Fluor® 700 Mouse Anti-Human CD3 BD Biosciences SP34-2; Cat. # 557917
BV510 Mouse Anti-Human CD4 BD Biosciences L200; Cat. # 563094
PerCP-Cy™5.5 Mouse Anti-Human CD8 BD Biosciences SK-1; Cat. # 565310
FITC Mouse Anti-Human TNF BD Biosciences MAB11; Cat. # 554512
APC Mouse Anti-Human IFN-γ BD Biosciences B27; Cat. # 554702
PE-CF594 Mouse Anti-Human CD69 BD Biosciences FN50; Cat. # 562617
APC Mouse Anti-Human CD235a/b BD Biosciences GA-R2; Cat. # 551336
PE Mouse Anti-Human CD71 BD Biosciences M-A712; Cat. # 555537
PE/Cyanine7 Mouse anti-human HLA-E Biolegend 3D12; Cat. # 342607
FITC Mouse Anti-Human CD8α Biolegend RPA-T8; Cat. # 301050
PE/Cyanine7 Rat anti-human TNF-α Biolegend W19063E; Cat. # 376210
APC mouse anti-human CD69 Biolegend FN50; Cat. # 310910
BV711 mouse anti-Human CD4 Biolegend SK3; Cat. # 344648
Alexa Fluor® 700 Mouse Anti-Human CD3 Biolegend HIT3a; Cat. # 300324
BV510 anti-human CD28 BioLegend CD28.2; Cat.#92582
Biotin anti-human CD197 (CCR7) BioLegend 93747;Cat.#G043H7
BUV395 Mouse Anti-Human CD4 BD Biosciences L200; Cat.#624165
BUV737 Mouse Anti-Human CD8 BD Biosciences SK-1; Cat.#624235
PE/Cyanine7 anti-human CD95 (Fas) BioLegend DX2; Cat.#80266
Pacific Blue™ Mouse Anti-Human CD3 BD Biosciences SP34-2; Cat.#624034
FITC anti-human TNF-α BioLegend Mab11; Cat.#624046
PE/Dazzle™ 594 anti-human CD69 BioLegend FN50; Cat.#93437
APC anti-human IFN-γ BioLegend B27; Cat.#96018
PE anti-human HLA-E BioLegend 3D12; Cat.#342604
PE anti-human IL-2 BioLegend MQ1-17H12; Cat.#500306
PE anti-human CD107a (LAMP-1) BioLegend H4A3; Cat. # 328608
PE CD49d anti-human (Integrin alpha 4) Life Technologies 9F10; Cat. # CUST03278
PerCP-Cy5.5 CD279 (PD-1) Life Technologies J105; Cat.# CUST05594
APC Rat Anti-Mouse CD3 Molecular Complex BD Biosciences 17A2; Cat. # 565643
PerCP-Cy™5.5 Rat Anti-Mouse CD8a BD Biosciences 53-6.7; Cat. # 551162
APC Rat anti-mouse IFN-γ Biolegend XMG1.2 ; Cat. # 505809
PE/Cyanine7 Rat anti-mouse TNF Biolegend MP6-XT22; Cat. # 506323
APC/Cyanine7 Rat anti-mouse TER-119 Biolegend Ly-76; Cat. # 116223
FITC Rat anti-mouse CD4 Biolegend GK1.5 ; Cat. # 100405
PE Rat anti-mouse CD69 Biolegend H1.2F3; Cat. # 104507

Blocking assays, ELISPOT assay and Western Blot
AAnti-mouse IFN-γ mAb unconjugated Mabtech AN18; Cat. # 3321-3-250
Anti-mouse IFN-γ mAb, biotin Mabtech R4-6A2; Cat, # 3321-6-250
Anti-ERAP1 Abcam EPR6069; Cat. # MABF747
Anti-TAPSIN Merck 7F6; Cat. # MABF249
Anti-F actin Abcam NH3; Cat. # ab205
Anti-b actin Sigma Cat. # A2066
Ultra-LEAF™ Purified anti-human HLA-A,B,C Biolegend W6/32; Cat. # 211441
VL9 peptide Genscript N/A
TH9 peptide Genscript N/A
Ultra-LEAF™ Purified anti-human CD3 Antibody Biolegend HIT3a; Cat. # 300334
Ultra-LEAF™ Purified anti-human CD28 Biolegend CD28.2; Cat. # 302933
Anti-human IFN-γ mAb, unconjugated Mabtech 1-D1K; Cat. # 3420-3-250
Anti-human IFN-γ mAb , biotin Mabtech 7-B6-1; Cat. # 3420-6-250
Ultra-LEAF™ Purified anti-human HLA-DR Antibody BioLegend L243; Cat.#307665

Validation

Each antibody and dye were validated as per the manufacturer's instructions. Antibodies were all validated for species specificity and

Validation	application by verifying staining for antigen-expressing cells but no staining for non-expressing cells. The dilution of antibodies and dyes were titrated to obtain the best concentration to use that stained positive cells but not negative cells. Gates were set using unstained and isotype control antibodies and/or just secondary antibodies.

Eukaryotic cell lines

Policy information about cell lines and Sex and Gender in Research

Cell line source(s)	721.221 cell lines were originally obtained from ATCC (CRL-1855). HLA-expressing cells were generated in the laboratory of Dr. Gaurav D. Gaiha.<br><br>K562 cells knockout for classical HLA (K562 HLA-/-); and K562HLA-/- overexpressing HLA E*01:03. Both cell lines were obtained from Dr. Federica Salusto (Institute for Research in Biomedicine, Switzerland).
Authentication	Cells were intracellular and extracellular stained with antibodies anti-pan-HLA-I (W6/32) or anti-HLA-E (3D12). The expression was accessed by flow cytometry.
Mycoplasma contamination	All cell lines were tested for mycoplasma contamination, being negative for it.
Commonly misidentified lines<br>(See ICLAC register)	*Name any commonly misidentified cell lines used in the study and provide a rationale for their use.*

Animals and other research organisms

Policy information about studies involving animals; ARRIVE guidelines recommended for reporting animal research, and Sex and Gender in Research

Laboratory animals	For non-human primates, Indian origin, captive-bred macacca mulatta (rhesus macaques) were used and between the ages of 2.5-7 years old.<br><br>Humanized-mice (n=3) at 10-12 weeks old.<br><br>C57BL/6j female mice 8-10 weeks old.
Wild animals	No wild animals were used in the study.
Reporting on sex	For non-human primates, an equal ratio of male and females was requested although not specifically included in study design or power analysis as a variable. Mixes of sexes were obtained for the n=7 cohort of Fig. 3a (3 male; 4 female) and the n=4 cohort of Fig. 3i (2 male; 2 female), but not for the n=3 cohort of Fig. 3d (3 male).<br><br>Humanized-mice (2 males and 1 female).<br><br>For wild type C57BL/6j experiments, only female mice were employed in this article. Base in our previous studies, male and female respond equally to P.yoelii chanlenge.
Field-collected samples	No field-collected samples were used in the study.
Ethics oversight	For non-human primate studies, the Oregon Health and Science IACUC approved all studies (protocols IP00003877, IP00001114, and IP00001169).<br><br>For humanized liver mice the study was reviewed and approved by the Institutional Animal Care and Use Committee of the Oregon Health and Science University (IACUC protocol number: IP00002077).<br><br>For C57BL/6 mice studies, procedures were performed following the National Council for the Control of 1087 Animal Experimentation (CONCEA). The protocols were approved by the Oswaldo Cruz Foundation 1088 (FIOCRUZ) Animal Experimentation Council (CEUA protocol LW-2/21).

Note that full information on the approval of the study protocol must also be provided in the manuscript.

Plants

Seed stocks: NA

Novel plant genotypes: NA

Authentication: NA

Flow Cytometry

Plots

Confirm that:

☒ The axis labels state the marker and fluorochrome used (e.g. CD4-FITC).

☒ The axis scales are clearly visible. Include numbers along axes only for bottom left plot of group (a 'group' is an analysis of identical markers).

☒ All plots are contour plots with outliers or pseudocolor plots.

☒ A numerical value for number of cells or percentage (with statistics) is provided.

Methodology

Sample preparation: Human PBMC were isolated from blood from healthy donors or Plasmodium vivax or P. falciparum infected patients using Ficoll (GE Healthcare) density gradient centrifugation (40 min, 500g) and cultured for 12 h in the presence of peptides. In some cases, PBMC were subject to blocking reagents (mAb W6/32, isotype control, VL9 ot TH9 peptides) for one hour before adding the stimulatory peptides. After incubation, brefeldin A was added and incubated for 4 hours.

Non-human primates PBMC were isolated and processed as per human PBMC.

Non-human primates liver resident T cells were obtained from liver biopsy. Tissue was weighed and rinsed in base buffer, followed by vigorous mechanical dissociation into a minced slurry using a vegetable chopper. Liberase TL was added to tissue homogenate, incubated at 37°C for 30' and strained through a 70-micron cell strainer. The resulting liquid suspension was centrifuged at 50xg for 2 minutes to remove hepatocytes. The supernatant was pelleted at 350xg for 5 minutes and pellet resuspended in ACK buffer to remove red blood cells. Cells were washed, pelleted by centrifugation and resuspended in 10mL 20% Opti-prep overlayed with 10mL PBS containing 2% FBS. NPCs were isolated from the gradient interface after centrifugation at 1500xg for 25 minutes with no brake. T cell stimulation was performed as described for human PBMC.

Mouse splenocytes were obtained after mechanical dissociation of spleens and passage through a 40 µm cell strainer. In some cases, CD8+ T cells were positivelly using an CD8a+ T Cell Isolation Kit (Miltenyi Biotec). Murine CD8+ T cells were cultured in RPMI 1640 supplemented with 10% FCS, 1% Pen/Strep and 1% L-glutamine, and 100 units of IL-2 in the presence or absence of stimulatory peptides. To assess cytokine productions, mouse splenocytes or CD8+ T cells were stimulated as per human PBMC.

Instrument: Flow cytometry aquisitions were performed using a BD FACSCelesta (3 lasers), a BD LSRFortessa (3 lasers) or a BD LSR II.

Software: Data were acquired by BD FACSDiva software v 8.0, then analyzed using FlowJo software V.10.10 (BD).

Cell population abundance: The purity of isolated cells was evaluated by flow cytometry after staining with the appropriate antibodies (CD8+ and CD4+ T cells for human and non-human PBMC, liver T cells and mouse splenocytes. Mouse isolated CD8+ T cells displayed a minimum of 90% purity.

Gating strategy: FSC/SSC gates were used to select mononuclear cells. FSC/FSH gates were then used to gate on single cells. Cells were then gated on live cells. Live cells were further gated on CD3+ leukocytes and then different populations of immune cells based on their expression of distinct markers. When needed, isotype control antibody staining was used to define positive/negative cell populations.

☒ Tick this box to confirm that a figure exemplifying the gating strategy is provided in the Supplementary Information.
