# Supplementary material for: Identification of cross-stage, cross-species malaria CD8+ T cell antigens
Source: Nature. 2026 Jul 1;655(8123):747–57. doi: 10.1038/s41586-026-10730-1 (PMC13661247; doi:10.1038/s41586-026-10730-1)
Supplement: Supplementary file 2 [file 41586_2026_10730_MOESM3_ESM.pdf]

nature portfolio

Peer Review File

Identification of cross-stage, cross-species malaria CD8+ T cell antigens

Corresponding Author: Professor Caroline Junqueira

This file contains all reviewer reports in order by version, followed by all author rebuttals in order by version.

Version 0:

Reviewer comments:

Referee #1

(Remarks to the Author)
Review of Junqueira et al.

The study of Junqueira et al. is an important body of work identifying a suite of potential immunogenic T-cell antigens that could form the foundation for future malaria vaccines against a wide-range of Plasmodium species that cause the disease.

The study builds on their previous work demonstrating that CD8+ lymphocytes recognise and lyse P. vivax (Pv) infected erythrocytes, pointing to antigen presentation on these immature erythrocytes (as compared to normocytes which are thought to be HLA null). Whilst in the published literature, CD8+ mediated immunity against malaria is thought of as liver-specific, this previous work presents an alternative lens on cellular immunity, i.e. where this is also relevant for blood stages (at least in Pv). The presence on both tissues is intertwined in the current work.

Overall, this work represents the most extensive HLA-I-bound Plasmodium peptide dataset to date and underscores the importance of CD8+ T cell immunity. It points to an underappreciated role for non-classical HLA antigen presentation pathways in malarial immunity, and provides a foundational step toward a cross-stage, cross-species vaccine strategy.

The authors should be commended for the extensive work, the number of Plasmodium models incorporated. The study will no doubt have impact well beyond this initial work.

Given the complexity of the work, I will walk through my understanding (in case I make an error) interjecting key questions I would be keen to hear responses from. I am not suggesting any major revision is required in terms of additional experiments, however, some changes or embellishments in text may aid understanding – which I have pointed to below:

Overview and key questions:

The study commences with the identification of peptides by mass spectrometry from the surface of Pv infected, patient-derived reticulocytes. This leads to the identification of 166 Pv proteins that are the targets of antigen presentation by retics – a herculean effort by any standard, significantly boosting our knowledge of antigen presentation in malaria.

Among peptides discovered, most are derived from housekeeping proteins such as ribosomal subunit proteins, histones, and ETRAMPs. Given their key cellular roles it is not surprising these are also expressed in other lifecycle stages and that there is wide conservation not just in Pv but also with orthologues in P. falciparum (Pf) [which is only distantly related phylogenetically]. Following this, over 30 Pv proteins identified share >80% sequence identity with Pf counterparts, highlighting their potential as cross-species vaccine candidates.

Functional validation of immunogenicity revealed that peripherally isolated T cells from Pv- and Pf-infected individuals, CD8+ especially, robustly recognized a subset of 50 conserved peptides (when compared to various healthy donors), supporting the notion of cross-species T-cell epitopes.

1. In the back of my mind is that Pf infects both normocytes and reticulocytes. Is there any surprise that peripherally isolated PBMCs are recognising these proteins or do the authors feel the degree of reticulocyte infection by Pf is sufficient (as per Pv)

to elicit a cellular immune response in this way (i.e. independent of the liver). Could the authors, for example, have done the same experiment with Pf-reticulocyte infected cells? Clarification around this (using Pf to validate but their biological differences) would be good.

The classification of HLA presented antigens into in silico defined binders and non-binders is then used to highlight that many of the target peptides are actually non-classical binders, pointing a finger of suspicion at non-classical presentation (i.e. HLA-E).

2. The logic here just about works, but the reliance on in silico definition of binding / non-binding is only as good as the NetMHCpan algorithm – which isn't perfect. It might have been good at this point to do some SPR analysis on a few peptides just to show their binding. Is there a danger here that it's being used as a slightly disingenuous segue from identification to HLA-E?

Building further on the non-classical HLA presentation, blocking HLA-E / T-cell interactions with an inhibitory peptides (VL9) definitively reduces some of the CD8+ T cell responses (50% of those tested), underscoring the key functional role this HLA-E presentation plays in blood stage immunity for malaria.

Having established the identity of target proteins conserved between Pv and Pf, and the role of HLA-E presentation, the authors switch to non-human models, starting with non-human primates.
Using P. knowlesi (Pk) NHP infections via sporozoite challenge – giving rise to blood patency and curing – generates a lymphocyte data set that can then be probed against pools of peptides (focussed on those that are 100% conserved). CD8+ T-cell responses confirmed the cross-species recognition of these key peptides (to varying degrees) between human and primate malarias, with infected-dependent boosting.

3. I am curious why Pk was chosen as the first model, and that infection was established using sporozoite immunisation. Like Pf, Pk also shows no tropism to reticulocytes, so will have a predominant of normocyte blood stages of infection. Further confounding the picture here, the route of immunization is via sporozoites, so there will be an element of circulating immunity deriving from liver infection. Does this conflation of blood/liver/retic/normocyte make it hard to genuinely interpret what is happening here in terms of boosting and by what route of antigen presentation? Is there a risk that liver stage might impact blood stage immunity? Some clarification around this feels important to address any potential conflation arising.

Speaking somewhat to my question, chemoprophylaxis vaccination (CVac) in NHPs is used to isolate the liver-specific response. Investigation of liver-derived lymphocytes showed a predictable response with boosting to known liver-specific T-cell targets (e.g. RPL6). Blood-derived lymphocyte responses, however, only showed boosting to the novel peptide pools.

4. Are the authors claiming here then that, for example, SSP2 and RPL6 are bona fide liver-specific T-cell targets, whereas the peptide pool (and it's antigens) are presented during both stages? I feel this could be more cleanly stated – de-conflating some of the intertwining of blood/liver in this section.

The final work with NHP involves related P. cynomolgi (Pcy, closely related to Pv and reticulocyte trophic) to validate some of the cross-species conservation of targets (35/50 peptides tested) and the involvement of HLA-E (which is conserved as Manu-E in rhesus macaques). Given this is delivered via blood stage inoculation, I find this section much clearer and more compelling in terms of conservation of targets.

5. I wonder whether if the focus of the work is HLA-E presentation, and conservation whether the Pcy work might not have taken priority over the Pk work?

Finally, leaving human and non-human primate models, the final analysis of antigen targets and their conservation is focussed on the rodent malaria model, P. yoelii (Py) which favours murine reticulocytes and is self-resolving.

As with Pcy, Pv infections are established via blood stage inoculation to explore the reactivity of mouse lymphocytes against the conserved peptides (several of which reacted); and then a focus on two specific targets 60S RPL29 and 40S RPLS30 (as broad conserved responders across the species models tested).

Splenocytes stimulated with conserved peptides from these two proteins elicited in vitro killing of Py infected erythrocytes and, as expected, reduced parasitemia when adoptively transferred to infected mice. The final experiment, then immunised mice with recombinant versions of these 2 proteins, demonstrating their capacity to reduce – albeit modestly – infection levels in the blood stages.

6. The modest level of reduction is somewhat disappointing, and it would be good to comment on why a more potent response wasn't seen, e.g. sterile protection (perhaps this is the nature of any future blood stage vaccine incorporating a CD8 T-cell component – it'll require many antigens). Also, it might have been good to test liver samples in these immunised animals, as my understanding is CpG whilst not naturally liver-tropic can influence liver immune responses – e.g. RPL6, combined with a CpG adjuvant, can induce robust liver-resident memory $CD8^{+}$ T cell responses in mice.

The authors conclude that these two proteins, and likely more from their broad study, are conserved across species and lifecycle stages, and make compelling candidates for exploring a universal, T-cell focussed malaria vaccine.

7. I would have liked to see use in the context of a lethal infection like P. berghei to explore the potential of these antigen

targets to elicit a properly protective response and in particular to isolate the liver-specific response (eluded to in the NHP work) which may make a very powerful and attractive vaccine approach (emulating RPL6). i.e. sporozoite immunization.

8. Finally, I would have loved to see a head-to-head comparison with RPL6 in this study as it feels like it would be important in benchmarking where this data will eventually land.

Some outstanding questions/points:

9. If HLA-E is so important, and there's a degree of cross species reactivity, for Pf, is this based on reticulocytes as well (even though Pf has no preference for retics) or is there an assumption here that this is also true of liver stages?

10. I am struck by the recollection that many in the field have talked a lot about how exported proteins will likely be T-cell targets. Yet here, the repertoire of hits is dominated by house-keeping cytoplasmic proteins. What then is the likely mechanism the authors feel underlies how these proteins get presented? Leakage across the parasitophorous vacuole?

11. I think the authors need to do some clarification around immunogenic versus protective – as it's the latter that we desperately need to identify without being distracted by the former. Is there anything in their work, beyond the 2 tested and the iRBC lysis assay, that could advance some of the other targets further as protective antigens for inclusion in a vaccine? Will we have to test them one at a time? What is our best predictor to date?

Some minor points:

Line 59: "making inducing T cells a major challenge". It might be good from the start to clarify that it's not just T-cell induction, but induction of protective T-cell responses that is desired.

Throughout: There is a bit of inconsistency with the titling of ELIspot/ELISpot, I believe the correct abbreviation for the Enzyme-Linked Immunospot assay is ELISpot (capital S).

Line 203: One protein is listed as coming from 50S RP3? Is this a mito- or apiocoplast ribosomal protein?

Line 245: "NHP were infected with 2,500 cryopreserved P. knowlesi (Pk)". Presumably IV?

Line 355: "CD4+ T cell antigens derived from Leishmania presented on mouse dendritic cell Major histocompatibility complex (MHC) II molecules were characterized by andem mass spectrometry (MS/MS)". Very minor grammatical errors: Major should be major, andem should be tandem.

Line 439: "T cell dependent." Should this be T-cell dependent manner?

Figure 3: I found panels b and e particularly hard to interpret – is there an easier way of displaying this data?

Referee #2

(Remarks to the Author)
This manuscript reports the first unbiased identification of Plasmodium spp. antigens presented via HLA class I on Plasmodium-infected reticulocytes, by immunopeptidomic analysis. Of the 166 proteins identified, most were housekeeping genes. Of the others, many were highly conserved between P. falciparum and P. vivax. The authors further showed that some were immunogenic in nonhuman primates following P. knowlesi infection and attenuated parasite immunization; and that two antigens were protective against P. yoelii challenge in mice. The authors suggest their data support the potential of a cross-stage and cross-species malaria vaccine.
This is an impressive study that addresses a critical gap in malaria vaccine development by identifying conserved Plasmodium vivax T cell antigens presented via HLA class I on infected reticulocytes. The use of immunopeptidomics to map naturally presented epitopes, combined with rigorous validation across Plasmodium species and infection models, including human patients, non-human primates, and mice, adds considerable strength to the findings. The demonstration of cross-stage and cross-species immunogenicity significantly enhances the translational relevance of these antigens for future vaccine design. The methods are clearly described and well presented, and in general the text is well written although there are a number of areas where clarity and impact should be improved (see below). Overall, the work is technically robust, conceptually innovative, and of high relevance to the field of malaria immunology and vaccinology.

• The Abstract states that most of the 166 different proteins identified by immunopeptidomic analysis were housekeeping proteins, and that many peptide sequences were highly conserved in P. falciparum and P. vivax. For clarity, the authors should specify exact numbers: how many non-house-keeping proteins were identified; how many non-house-keeping proteins were tested for antigenicity in P. vivax- and P. falciparum infected humans, or Plasmodium-infected/immunized primates (and what Plasmodium species); how many were tested for protection in mice, and what the results were.

• The vast majority of identified peptides were derived from essential housekeeping proteins (line 409), and more than half from ribosomal proteins; but arguably use of housekeeping proteins as vaccine targets may not be a biologically appropriate vaccine strategy. The authors should clarify and expand their presentation (in abstract and in main text) of findings with the non-housekeeping proteins as potential vaccine candidates; and justify how vaccine based on housekeeping genes could be developed into an effective vaccine for humans.

• The study does not explore $CD4^+$ T cell responses or HLA class II restricted epitopes, which are important for the induction and maintenance of $CD8^+$ T cell responses, for providing help for optimal antibody responses, and for coordinating broader long-lasting protective immunity. Given the importance of $CD4^+$ T cells in effective vaccine-induced protective immunity, the authors should clarify their rationale for focusing exclusively on HLA class I epitopes and address whether class II restricted responses were considered or assessed. This focus exclusively on CD8+ T cells seems to be a deficiency in an otherwise comprehensive study, especially since many longer peptides (10-16mers) were identified (line 103).

• The sample IDs in Figure 1a (e.g., 24, 26, 28) are not clearly explained. Manuscript text (line 94) states that "Patient samples were anonymized as 24, 26, 28, 30, 31, 34, and 35". This should, therefore, be stated explicitly in the figure legend to avoid confusion about potential experimental or temporal ordering. Suggested rewording: "Samples (labeled 24, 26, 28, 30, 31, 34, and 35) were anonymized identifiers assigned arbitrarily to each sample."

• In Figure 1a, the transition from All peptides to P. vivax–specific peptides is logical, but it is not explicitly stated that the per-sample counts shown (Total and Unique) represent only peptides matching the P. vivax Sal-1 proteome. Please clarify this in the figure legend to avoid confusion.

• Legend Figure 1c "indicated by colour intensity where light blue is the minimum and dark pink the maximum" is vague - minimum of what? It seems to refer to number of patients, which is already on the x-axis. The figure legend should be revised for clarity, e.g., to explicitly state that: (i) the x-axis represents the number of patients, (ii) the y-axis shows percent identity to P. falciparum orthologs, (iii) dot size reflects the level of sequence identity, and (iv) dot color ranges from light blue (minimum patients) to dark pink (maximum). Also, including a color bar and size scale legend within the figure would further enhance clarity for the reader

• Figure 1d: The figure uses similar or almost identical colors to represent different groups, making it difficult to distinguish between them. It is unclear which percentage corresponds to which group. The color coding should be clarified to improve interpretability.

• Figure 1 legend: the lettering that refers to individual panels is inconsistently formatted - sometimes following a comma, other times in parentheses. Please ensure consistent formatting of panel labels throughout the figure legend.

• Figure 2a: The legend refers to continuous data representing individuals from 2 to 5, but the figure itself displays discrete values. This makes interpretation difficult - for example, it is unclear whether peptides shown in green are recognized by 2 or 3 individuals. Please ensure that the legend accurately reflects the discrete nature of the data.

• Figure 2a-b includes only 5 of the 7 samples mentioned in the text. If the other 2 samples were excluded because they did not share peptides with any others, this should be explicitly stated in the figure legend or main text to avoid confusion.

• Extended Data Figure 4: The legend appears to be incomplete, with panel d missing from the description. The text currently ends after the explanation of panels b. Please include a description of panel d to ensure the figure is fully interpretable.

• Line 56 (and elsewhere; e.g. line 176): should be anti-Plasmodium T cell response, not anti-malaria T cell response; malaria is the disease.

• Line 93: please confirm that the "7 acute malaria patients" were confirmed to be infected with P. vivax, and not P. falciparum – and include details of parasite load and clinical details etc as a supplementary table.

• Line 94: should refer to detailed HLA typing information of the subjects.

• Line 103: Include text noting that 8-10mers would be expected bot be class I restricted CD8+ T cell epitopes, whereas the longer 10-16mer peptides would likely be CD4+ T cell epitopes, and cite the relevant literature.

• Lines 104-105: move the sentence describing Pv/Pf conservation to after the text describing frequency of identification (lines 106-117).

• Lines 108–111: The identity percentages stated in the text ("These proteins share 83% and 63% amino acid sequence identity…") do not appear to match the y-axis values shown in Figure 1c. For example, ETRAMP (A5K676) appears below 60% on the graph, and 40S S2 does not reach 97% as claimed. Please verify whether the values plotted on the y-axis are correct or if the numbers reported in the text need adjustment.

• Conservation of identified peptides/proteins between all Plasmodium species should be clearly presented as a Table or Figure (or at least for the most relevant subset) – line 243. Currently, the focus is on conservation between Pv and Pf, but conservation in relation to P. knowlesi and P. yoelii is particularly relevant to the study design, and conservation across all species is relevant to the author's message of a cross-species vaccine.

• Line 126-129: clarify how may proteins were tested for liver-stage expression, and what the rationale was for selecting this subset. The statement "All genes that were tested were found to be expressed in Pf liver stages" lacks context.

• Line 126 and Extended data Figure 1. The RT-qPCR validation of Pf transcript expression in infected humanized mouse liver is informative, but the absence of a clearly stated negative control raises concerns about specificity. Inclusion of a negative control, such as uninfected humanized liver tissue, would strengthen the interpretation of Pf-specific expression during the liver stage. If such a control was not used, this limitation should be acknowledged. Alternatively, if uninfected controls were tested but not shown, it should be stated that no expression was detected.

• Line 139/140: state how many Pv or Pf subjects were tested by IFNg ELISpot.

• Line 145: The authors report no difference in IFNγ-producing SFCs between Pv- and Pf-infected patients. However, Figure 1f appears to show a lower percentage of Pf-infected individuals responding to the peptide pool compared to Pv-infected individuals. This difference in responder frequency is not statistically evaluated or discussed. Could the authors clarify whether this difference was observed to be significant, and if so, comment on its potential biological relevance, for example, whether it reflects differences in immunodominance, antigen exposure, or host response dynamics between the two infections?

• Line 159: The sentence is incomplete - "IFNγ in response to some of the peptides (Fig. 1e, f). In general, the…" ends abruptly. Please correct.

• The authors should explain why they selected the NetMHCpan program for epitope prediction algorithm in preference to other computational a=tools, and note any limitations, and what a threshold of 10% means.

• Lines 207–209: The sentence stating that "non-binder" peptides had significantly higher positive responses (55.2%) than "binder" peptides (32% reactivity) is confusing. It is unclear whether the percentages refer to the number of peptides eliciting positive ELISpot responses or to the magnitude of IFNγ responses (e.g., SFCs). If it refers to the proportion of peptides with positive responses, this should be clearly stated. Additionally, the statistical test used to compare these groups should be reported in the main text or figure legend to avoid misinterpretation.

• Lines 210-218 and 220-240: The authors propose that the unexpected immunogenicity of "non-HLA-ABC binder" peptides may result from deficiencies in peptide quality control and trimming in Pv-infected reticulocytes (iRetics), due to the absence of ERAP1 and Tapasin. This hypothesis is further supported by subsequent experiments showing HLA-E expression on iRetics and reduced IFNγ production by $CD8^+$ T cells upon HLA-E blockade. Together, these findings suggest that some of the immunogenic peptides may be presented through non-classical HLA-E rather than classical HLA-ABC pathways. However, the connection between these two sections (lines 210–218 and 220–240) could be made more explicit to help the reader follow the progression from the observed responses to the mechanistic explanation.

• Line 256: "In light of published data…" – these publications should be cited

• Lines 256- 276: The lack of boosting in liver $CD8^+$ T cell responses to CSP (Fig. 3g,h) is attributed to a single animal with unchanged responses between immunizations. While this may be a contributing factor, it would be helpful if the authors could comment more broadly on why CSP-specific responses might fail to boost despite repeated sporozoite exposure, particularly given its relevance as a vaccine target.

• Lines 293-295: The observation that VL9 blocking is not strictly peptide-dependent but varies between animals is intriguing. Further commentary on potential explanations (e.g., Mamu-E allelic differences, TCR repertoire diversity) would enrich interpretation.

• Lines 328-339: The final experiment showing protection upon immunization with recombinant RP L29 or S30 strengthens the translational value of these targets. However, the authors should clarify whether the observed protection reflects short-term effector T cell responses or involves memory T cell induction, as this has implications for vaccine durability and design.

• Line 381: The sentence is missing a verb. Please revise e.g., "the ability of certain peptides to bind to various HLA-I molecules."

• Line 976: two full stops are present instead than one.

Referee #3

(Remarks to the Author)
I co-reviewed this manuscript with one of the reviewers who provided the listed reports.

Referee #4

(Remarks to the Author)
The authors present their findings from an immunopeptidomic approach combined with mass spec analysis of peptides bound to HLA-I from reticulocytes of patients infected with Plasmodium vivax. They provide extensive characterization of the immunogenicity of many of these peptides using different animal models and clinical samples. A few peptides, particularly

from ribosomal proteins, stand out as candidates to elicit CD8+ T cells that could promote parasite killing in the liver and against Pv-infected reticulocytes. Overall, these data are of interest to the field and could inform vaccine design, but the study itself is not considered of sufficient interest to a broader audience. The methodology is not novel and has been used to identify T cell epitopes from other infectious pathogens (discussed by the authors). Also, the data are not considered immediately impactful.

Major points:
• As a general concern, the data are overinterpreted in many parts of the text including the Abstract and the Discussion. Some P values are very close to 0.05 and in certain examples, the authors generalize data across groups of samples or animals despite extensive variability. The statement in the Abstract that the epitopes were validated in Pv- and Pf-infected samples is an overstatement. This was done with a small number of donors and only from one geographical/epidemiological area. As another example of an overstatement, Line 85: "These epitopes were immunogenic in Pv- and Pf-infected patients, P. cynomolgi (Pcy)- and P. knowlesi (Pk)-infected non-human primates (NHP) and P. yoelii (Py)-infected mice".
• The authors state >99% infected reticulocytes, but there is no mention of how uninfected reticulocytes or RBC contamination was ruled out. This could confound the peptide repertoire observed.
• In the data from the immunopeptidomic analysis, there seems to be a bias where sample 35 yielded excessively more peptides than the other samples. This is not discussed.
• A large proportion of identified peptides are derived from housekeeping proteins, such as ribosomal subunits and histones. While the authors showed that these are highly conserved and expressed across parasite stages, their abundance and essential nature raise the possibility that responses may reflect immunodominance due to abundance rather than physiological relevance or protective efficacy. This should be discussed more in the context of antigen selection for vaccines.
• The central conclusion that these epitopes could support a "universal malaria vaccine" is premature, especially in the absence of direct evidence of protection in liver-stage models or human challenge studies. The in vivo killing assays in mice are informative but limited in scope and species relevance.
• The ELISPOT data in Fig. 1e do not show very strong statistical significance. For the Pv-infected patients, there seems to be a major population that does not respond and a minor population that does. I think the control should be an irrelevant peptide or one that does not meet their criteria for selection.
• The sample size for the Pf-infected PBMCs used for ELISPOT analysis was very small and differences were not significant compared to endemic controls. The experiments should be extended with a larger sample size given the importance of identifying cross-species peptides that elicit T cell responses.
• The responses in Fig. 1f are much lower in the Pv symptomatic and asymptomatic cohorts compared to the samples included in Fig. 1e. It is not clear whether these are entirely different samples, and the authors should provide an interpretation of these findings. Also, their result that there is no difference between symptomatic and asymptomatic patients in terms of responses to peptides calls into question whether they could play a role in protection.
• The immunological characterization of $CD8^+$ T cell responses lacks critical functional depth. The authors assess IFN-γ production but do not examine T cell polyfunctionality (e.g., TNF, IL-2) or cytotoxic capacity (e.g., granzyme B, perforin, CD107a). Without these markers, it is difficult to assess whether these $CD8^+$ T cells are functionally relevant or protective.
• For broader impact, it is important to show responsiveness to these peptides in another population. As HLA restriction is central to $CD8^+$ T cell epitope presentation, the limited HLA diversity in this cohort restricts the translational relevance of the findings.
• The apparent disconnect between predicted peptide–MHC binding and observed T cell responses is not addressed mechanistically. In fact, several peptides categorized as "non-binders" elicited stronger responses than "binders," which raises questions about whether these are genuinely MHC-I–restricted $CD8^+$ T cell responses or due to alternative mechanisms (e.g., HLA-E presentation, innate activation, or off-target effects). This challenges the current understanding of MHC class I peptide selection and suggests either non-classical presentation or assay artifacts. While the authors suggest that the responses may be mediated by non-classical HLA-E presentation (Fig. 2h-j), this is not experimentally confirmed beyond VL9 peptide blocking assays, making the HLA-E restriction claim speculative. Other assays could be considered to validate this claim.
• Line 285: the authors state that all animals produced anti-CSP IgG but one of them only produced low antibodies after the third vaccination.
• For the murine model, the specificity of the cell killing for the peptides from their dataset would be stronger if they showed that one of the non-stimulatory peptides had no killing effect in this assay.
• The Discussion fails to discuss the potential advantages of this approach compared to whole parasite Pf or Pv vaccines, such as PfSPZ or PvSPZ. If those epitopes are expressed by sporozoites and conserved, then a whole parasite vaccine would present these epitopes and contribute to cross-stage immunity. Generally, the Discussion mostly reiterates the results and does not provide enough discussion of the literature and the broader implications of these findings for vaccine design.

Minor points
• Ext data table 1 shows homology, not identity. This contradicts with the text.
• Transcriptomic databases do not necessarily reflect protein expression at the other life cycle stages.
• Are the peptides that elicit the strongest responses across species also conserved in other organisms (eg identity to human sequences, other pathogens?)
• Line 159: Incomplete sentence

Referee #5

(Remarks to the Author)
I co-reviewed this manuscript with one of the reviewers who provided the listed reports.

Version 1:

Reviewer comments:

Referee #1

(Remarks to the Author)
Review of revised manuscript by Barbosa et al.

As this is a revised manuscript, I will limit my comments. Simply put, I am comfortable that the authors have addressed all my initial questions and concerns adequately and had a fair go at comments from the other reviewers. The addition of the substantial new data, including new human cohorts (endemic and CHMI) as well as mechanistic validation of HLA-E presentation and exploration of liver-resident CD8+ T cell responses upon ribosomal protein immunization add substantial new supporting data to what was already a herculean effort with extensive data. The overall study will be of profound interest to the malaria immunology and vaccinology community both as a resource - in particular the identification of broadly conserved antigenic targets of cellular immunity - to compare and explore further T-cell responses and T-cell vaccines in general but also as a launch pad to move the current candidates already identified forwards towards translation. The authors should be commended on an impressive body of work.

JB.

Referee #2

(Remarks to the Author)
I commend the authors for an informative, comprehensive and well written manuscript. The authors have expanded the originally submitted study to comprehensively address most of the concerns raised upon peer review, although there are two areas considered out-of-scope that would strengthen the manuscript:

Reviewer 1: Head-to-head study with RPL6 - a single (repeated) protection study comparing RPL6 against the novel CD8 antigen leads would have been highly informative, and (in my opinion) not a lot of extra work given the comprehensiveness of the current study.

Reviewer 2/3: $CD4^+$ T cell responses - although the experimental strategy focused on HLA class I elution, and class II immunopeptidomic studies would be outside the scope of the current manuscript, which focuses on identification of novel targets of CD8+ T cell immune responses, the authors could nonetheless have assessed their novel vaccine targets for CD4 T cell responses in vitro, and could also have conducted the in silico HLA-class II peptide binding studies, to provide some consideration to this important cell type.

Reviewer 2/3 #Lines 328-339: whether protection reflects short-term effector T cell responses or involves memory T cell induction - OK, although a challenge study conducted at 90-days post immunisation using a well established T cell inducing adjuvant could have (at least in part) addressed this issue.

Referee #4

(Remarks to the Author)
The authors added important new data and modifications to the text that address my original concerns.

Referee #5

(Remarks to the Author)
I co-reviewed this manuscript with one of the reviewers who provided the listed reports.

Open Access This Peer Review File is licensed under a Creative Commons Attribution 4.0 International License, which permits use, sharing, adaptation, distribution and reproduction in any medium or format, as long as you give appropriate credit to the original author(s) and the source, provide a link to the Creative Commons license, and indicate if changes were made.
In cases where reviewers are anonymous, credit should be given to 'Anonymous Referee' and the source.
The images or other third party material in this Peer Review File are included in the article's Creative Commons license, unless indicated otherwise in a credit line to the material. If material is not included in the article's Creative Commons license and your intended use is not permitted by statutory regulation or exceeds the permitted use, you will need to obtain permission directly from the copyright holder.
To view a copy of this license, visit https://creativecommons.org/licenses/by/4.0/

Rebuttal Letter

We thank the reviewers for their thoughtful and constructive comments. We appreciate the opportunity to revise our manuscript and clarify key aspects of our study. The key new results in the revised manuscript are:

1. **Expanded Cohorts for Broader HLA Diversity**

   To address concerns about translational relevance, we included *Plasmodium falciparum*- infected samples from Mali, a geographically distinct endemic region for malaria. In this way we expanded the number of *Pf* samples utilized to perform the validation of the peptides and at the same time to explore their antigenicity in a population with a completely distinct genetic background from the one the antigens were first identified and tested. In this population, we tested 20 of the peptide candidates and confirmed that most peptides were recognized by patient T cells, leading to robust IFNγ responses in comparison to endemic health donors (Fig. 1i).

2. **Controlled Human Malaria Infection (CHMI) with *P. vivax* blood-stage**

   We incorporated analysis from a controlled *P. vivax* blood-stage infection study in six volunteers from the United States, expanding the study to another independent geographic cohort. The volunteers were followed for 34 days and the antigen-specific $CD8^+$ T cell response was assessed, allowed us to confirm that the peptide-specific $CD8^+$ T cell immunity can be induced independently of liver-stage infection, supporting a direct role for blood-stage antigen presentation in shaping these responses (Fig. 1j).

3. **Mechanistic Validation of HLA-E Presentation**

   As suggested by the reviewers, we explored the non-classical HLA-E antigen presentation. We developed an assay using a K562 cell line engineered by CRISPR Cas9 to knock out classical HLA-I molecules while overexpressing HLA-E*01:03. Purified $CD8^+$ T cells from *Pv*-infected patients were primed with the 50 peptides pool, subsequently cocultured with peptide-pulsed K562 HLA-$I^{-/-}$ cells or K562 HLA-E*01:03. Antigen-specific responses were quantified by anti-IFNγ ELISpot, revealing $CD8^+$ T cell activation only in the presence of HLA-E–expressing cells. These results support our conclusion that, in addition to classical HLA-A, B and C, HLA-E also presents some of the identified peptides, leading to $CD8^+$ T cell activation (Fig. 2k).

4. **Liver-resident CD8+ T cell response upon ribosomal proteins immunization**

   One question that arose regarding our immunization methodology with recombinant proteins + CpG adjuvant was the ability of this formulation to induce activation of liver-resident $CD8^+$ T cell and then trigger protectivity immunity at pre-erythrocytic stage. To answer this question, we evaluated the response of resident $CD8^+$ T cells after the immunization protocol with 60S RP L29 and 40S RP L30. The new data represented as figure 4i show that both ribosomal proteins + CpG formulations were able to boost antigen-specific $CD8^+$ T in the liver.

Reviewer #1

We thank Reviewer #1 for their positive assessment of our work and for recognizing the novelty and breadth of our immunopeptidomic analysis. We appreciate the detailed questions and suggestions, which helped us to clarify several aspects of our study.

1. In the back of my mind is that Pf infects both normocytes and reticulocytes. Is there any surprise that peripherally isolated PBMCs are recognizing these proteins or do the authors feel the degree of reticulocyte infection by Pf is sufficient (as per Pv) to elicit a cellular immune response in this way (i.e. independent of the liver). Could the authors, for example, have done the same experiment with Pf-reticulocyte infected cells? Clarification around this (using Pf to validate but their biological differences) would be good.

Indeed, Pf preferentially infects reticulocytes, but since it is only ~1% of the total red blood cells (RBC) in circulation, it predominantly infects normocytes. Thus, we do expect that Pf-infected reticulocytes are also presenting antigens to $CD8^+$ T cells. Methodologically, it's impossible to separate infected reticulocytes (iRetics) from infected normocytes (iRBC) and still obtain enough iRetics to pursue functional assays (~10^7), and specially the immunopeptidome workflow (~10^8). This has also been an object of study for our group for some time but obtaining enough Pf-iRetics and $CD8^+$ T cells from the same patient has not yet been possible. Additionally, co-culturing all infected red blood cells without distinguishing between Retics (<3%) and normocytes (~97%), although inducing a small response, is not statistically different.

2. The logic here just about works, but the reliance on in silico definition of binding / non-binding is only as good as the NetMHCpan algorithm – which isn't perfect. It might have been good at this point to do some SPR analysis on a few peptides just to show their binding. Is there a danger here that it's being used as a slightly disingenuous segue from identification to HLA-E?

We acknowledge the limitations of *in silico* prediction tools. While MHC- antigens prediction tools provide useful insights, historically they are mainly focused on classical MHC and are not robust enough for HLA-E peptide prediction. In fact, we did some binding stability assays to HLA-E and HLA-A (Extended fig. 7), which, although weak, demonstrates the binding to HLA-E. Unfortunately, we did not have the technically capability to perform SPR in a timely manner. Thus, to better explore the whether the peptides identified in this study are presented by HLA-E, we performed an *in vitro* assay using a K562 cell line engineered by CRISPR to lack classical HLA-I molecules ($HLA^{-/-}$), while overexpress of HLA-E*01:03 or not. These cells were pulsed with the Total pool (all 50 synthesized peptides) and co-cultured with primed $CD8^+$ T cells isolated from *P. vivax*–infected patients. $CD8^+$ T cell activation was assessed by IFNγ ELISpot. As shown in figure 2l, $CD8^+$ T cells co-cultured with Total pool–pulsed K562 HLA-E*01:03 cells produced significantly higher levels of IFNγ compared to those co-cultured with Total pool–pulsed K562 $HLA^{-/-}$ cells. These results provide direct experimental evidence that a subset of peptides, previously attributed to "non-binder" peptides, is in fact mediated by non-classical HLA-E presentation, thereby supporting the mechanistic basis of our initial hypothesis.

Having established the identity of target proteins conserved between Pv and Pf, and the role of HLA-E presentation, the authors switch to non-human models, starting with non-human primates. Using P. knowlesi (Pk) NHP infections via sporozoite challenge – giving rise to blood patency and curing – generates a lymphocyte data set that can then be probed against pools of peptides (focussed on those that are 100% conserved). CD8+ T-cell responses confirmed the cross-species recognition of these key peptides (to varying degrees) between human and primate malarias, with infected-dependent boosting.

3. I am curious why Pk was chosen as the first model, and that infection was established using sporozoite immunisation. Like Pf, Pk also shows no tropism to reticulocytes, so will have a predominant of normocyte blood stages of infection. Further confounding the picture here, the route of immunization is via sporozoites,

so there will be an element of circulating immunity deriving from liver infection. Does this conflation of blood/liver/retic/normocyte make it hard to genuinely interpret what is happening here in terms of boosting and by what route of antigen presentation? Is there a risk that liver stage might impact blood stage immunity? Some clarification around this feels important to address any potential conflation arising.

Pertinent to this and point 5 below, we agree that the initial presentation of the NHP models was confusing. We have now tried to clarify that *Pk* provides an experimentally tractable and established system that shares key biological characteristics with *P. falciparum*. Given its use in sporozoite challenge and immunization, it allows us to study immunity in the context of liver and blood stages. As arranged now (*Pcy* blood stage infection to validate human results→Pk sporozoite infection as an established challenge model→Pk sporozoite immunization as an established liver-directed immunization), we hope this creates a more logical flow. While liver-stage exposure may influence blood-stage immunity, this reflects the natural infection process and is integral to understanding potential boosting mechanisms. Please see responses below for in-text modifications.

Speaking somewhat to my question, chemoprophylaxis vaccination (CVac) in NHPs is used to isolate the liver-specific response. Investigation of liver-derived lymphocytes showed a predictable response with boosting to known liver-specific T-cell targets (e.g. RPL6). Blood-derived lymphocyte responses, however, only showed boosting to the novel peptide pools.

4. Are the authors claiming here then that, for example, SSP2 and RPL6 are bona fide liver-specific T-cell targets, whereas the peptide pool (and it's antigens) are presented during both stages? I feel this could be more cleanly stated – de-conflating some of the intertwining of blood/liver in this section.

We agree this was not clear. We have rearranged the NHP studies and made changes to the text to directly address this.

In the results relevant to the sporozoite challenges, we tried to clarify the sporozoite vs. blood stage antigens by adding: "Interestingly, this was not the case for overlapping peptides covering more established antigens found on the sporozoite (CSP and sporozoite surface protein 2, SSP2) or for apical membrane antigen 1 (AMA1) which is present in the sporozoite and abundant at the blood stage (Fig. 3f)."

In the results relevant to the CVac immunization we added: "While responses to the three known antigens could be seen in the blood after the first immunization, these were not boosted by subsequent immunizations (Fig. 3h, i). This is similar to data in humans which fail to show a boosting of peripheral $CD8^+$ T cells with subsequent immunizations, likely due to the low/absent circulation of tissue-resident T cells in the liver (Murphy, Deye et al. 2021). In support of this, $CD8^+$ T cell responses in the liver were significantly boosted between vaccinations 1 and 3 to all antigens except CSP; although this failure to demonstrate significant boosting against CSP is likely driven by a single animal which had unchanged responses to CSP from immunization one to three (Fig. 3j,k). Together, these results demonstrate that at least a subset of these novel antigens are conserved in *Pk* and are antigenic in the context of both repeated sporozoite-induced blood stage infection and liver-directed sporozoite immunization.

Finally, in the Discussion, we have tried to further contextualize this with: "Our study reveals that repeated *Pk* blood stage infections induced by a small number of sporozoites can significantly boost $CD8^+$ T cell responses to conserved peptides, particularly to a pool of 9 peptides highly conserved between *Plasmodium*, while responses to more traditional antigens like CSP, SSP2, and AMA1 remain relatively unchanged. In the context of liver-directed CVac immunization wherein the sporozoite inoculation of the liver is much higher (50,000 vs. 2,500 sporozoites) and the blood stage severely diminished or absent at boost, we observed that pre-erythrocytic immunization with CVac enhanced T cell responses not only to established antigens such as SSP2 and RPL6 but also to Pool-9 antigens, suggesting that $CD8^+$ T cells targeting these newly identified antigens may play a crucial role in both blood and liver stage immunity."

The final work with NHP involves related P. cynomolgi (Pcy, closely related to Pv and reticulocyte trophic) to validate some of the cross-species conservation of targets (35/50 peptides tested) and the involvement of HLA-E (which is conserved as Manu-E in rhesus macaques). Given this is delivered via blood stage inoculation, I find this section much clearer and more compelling in terms of conservation of targets.

5. I wonder whether if the focus of the work is HLA-E presentation, and conservation whether the Pcy work might not have taken priority over the Pk work?

We agree that the *P. cynomolgi* (*Pcy*) model provides more direct evidence of reticulocyte-stage immunity and HLA-E involvement. To reflect this, we have reorganized the manuscript so that the NHP section now begins with the *Pcy* data, emphasizing its strength and relevance to our central hypothesis. The inclusion of *Pk* was intended to explore cross-stage and cross-species recognition of the identified antigens under conditions that mimic natural infection, do not include reticulocyte restriction in the blood, and include liver-stage exposure. This complements the *Pcy* findings by addressing antigen presentation across different parasite stages. Together, these models provide a broader perspective on conservation and immune mechanisms, which we believe strengthens the overall conclusions.

Finally, leaving human and non-human primate models, the final analysis of antigen targets and their conservation is focussed on the rodent malaria model, P. yoelii (Py) which favours murine reticulocytes and is self-resolving. As with Pcy, Pv infections are established via blood stage inoculation to explore the reactivity of mouse lymphocytes against the conserved peptides (several of which reacted); and then a focus on two specific targets 60S RPL29 and 40S RPLS30 (as broad conserved responders across the species models tested). Splenocytes stimulated with conserved peptides from these two proteins elicited in vitro killing of Py infected erythrocytes and as expected, reduced parasitemia when adoptively transferred to infected mice. The final experiment, then immunised mice with recombinant versions of these 2 proteins, demonstrating their capacity to reduce – albeit modestly – infection levels in the blood stages.

6. The modest level of reduction is somewhat disappointing, and it would be good to comment on why a more potent response wasn't seen, e.g. sterile protection (perhaps this is the nature of any future blood stage vaccine incorporating a CD8 T-cell component – it'll require many antigens). Also, it might have been good to test liver samples in these immunised animals, as my understanding is CpG whilst not naturally liver-tropic can influence liver immune responses – e.g. RPL6, combined with a CpG adjuvant, can induce robust liver-resident memory $CD8^+$ T cell responses in mice.

Despite the modest reduction in parasitemia, we did not expect to observe sterilizing immunity. Blood-stage challenge is very aggressive, and no vaccine formulation will be able to control parasitemia 100%. The most advanced vaccine for blood stage is the PfRh5, which was never tested in mice model, but failed to induce sterilizing protection in NHP model (Douglas, Baldeviano et al. 2015, Ilani, Nyarko et al. 2025). Furthermore, it also did not fully control parasitemia in CHMI (Minassian, Silk et al. 2021). Still, a single antigen vaccine that reduces parasitemia is an extremely important tool for the great goal of malaria elimination. Another important aspect is that our objective here was not to develop a final vaccine formulation, but to have a proof-of-concept that some of the identified new antigens have immunogenic and protective properties. Further studies should be performed to optimize the vaccine formulation, either by using different adjuvants, virus vectors, or mRNA platform. Regarding the CpG adjuvant, we did observe an induction of antigen-specific liver $CD8^+$ T cells (Fig. 4i). This desired characteristic should be better explored with sporozoite challenge in future studies.

The authors conclude that these two proteins, and likely more from their broad study, are conserved across species and lifecycle stages, and make compelling candidates for exploring a universal, T-cell focussed malaria vaccine.

7. I would have liked to see use in the context of a lethal infection like P. berghei to explore the potential of these antigen targets to elicit a properly protective response and in particular to isolate the liver-specific response (eluded to in the NHP work) which may make a very powerful and attractive vaccine approach (emulating RPL6). i.e. sporozoite immunization.

We appreciate the suggestion to explore liver-specific responses using a lethal rodent model such as *P. berghei*. However, our study prioritized models with greater translational relevance to human malaria. For this reason, we incorporated a non-human primate (NHP) model for *P. knowlesi*, which is phylogenetically closer to human infection and provides a more meaningful context for evaluating antigen conservation and immune mechanisms. While rodent models are valuable for certain experimental approaches, their immunological differences limit direct applicability to human malaria. Thus, we believe that the inclusion of NHP data significantly strengthens the relevance of our findings.

Additionally, as we demonstrated that our immunization protocol with recombinant proteins + CpG elicited liver-resident $CD8^+$ T cells (Fig. 4i), we tested the potential of these vaccine formulations (as a pilot test) to induce protective response against *P. yoelii* sporozoite challenge. We prioritized the *P. yoelli* model to be able to compare the immunological response at pre-erythrocytic stage and blood-stage (Py infects reticulocytes). However, we faced technical challenges with *P. yoelii* sporozoite infection, which model was not well established by our research team. Due to bureaucratic and logistical difficulties in shipping vaccines between laboratories/ countries, we were unable to complete the desired experiment. Despite this impossibility, we are confident that the absence of this data does not diminish in any way the high relevance of the work presented here, which had as its main objective the unprecedented identification of antigens presented via HLA-I.

8. Finally, I would have loved to see a head-to-head comparison with RPL6 in this study as it feels like it would be important in benchmarking where this data will eventually land.

We acknowledge the importance of RPL6, which has been shown to confer a bona fide sterilizing liver-stage immunity in rodent models following sporozoite challenge (Valencia-Hernandez, Ng et al. 2020). We included in our discussion a more detailed comparison of our finds to the RPL6 antigen, highlighting that our study brings an extensive value to the use of ribosomal proteins as an antigen for malaria vaccine, since we identified a broader set of ribosomal antigens, including 60S and 40S subunits, that are highly conserved, expressed across liver and blood stages, and antigenic in humans, non-human primates, and mice. Indeed, a study comparing RPL6 with the new antigens, herein identified, would be of great value to the field of malaria vaccine research. However, such a detailed head-to-head comparative study, which would itself be another manuscript, is outside the scope of the work presented here, which aimed at the unbiased identification of new $CD8^+$ T cell antigens.

9. If HLA-E is so important, and there's a degree of cross species reactivity, for Pf, is this based on reticulocytes as well (even though Pf has no preference for retics) or is there an assumption here that this is also true of liver stages?

Based on the cross-stage expression of the antigens identified in this study (Extended Fig. 2, SI. 4) and supported by the data from the Human Protein Atlas showing that hepatocytes express HLA-E (Uhlen, Fagerberg et al. 2015), we hypothesize that many of these antigens could also be presented by HLA-E during liver-stage infection. However, our group has historically focused on antigen presentation by reticulocytes, as demonstrated in (Junqueira, Barbosa et al. 2018). While the HLA-E presentation in the liver was not the main focus of the current study, it represents an important avenue for future investigation.

10. I am struck by the recollection that many in the field have talked a lot about how exported proteins will likely be T-cell targets. Yet here, the repertoire of hits is dominated by house-keeping cytoplasmic proteins.

What then is the likely mechanism the authors feel underlies how these proteins get presented? Leakage across the parasitophorous vacuole?

We agree that the predominance of housekeeping cytoplasmic proteins among the identified antigens is striking. We propose two non-mutually exclusive mechanisms that could explain their presentation: (i) protein translocation through PTEX, since most of the identified antigens contains export motifs, like PEXEL or PNEP, and (ii) leakage across the parasitophorous vacuole membrane (PVM), which may allow parasite-derived proteins to access the host cytosol and antigen processing pathways. Evidence from studies on *Plasmodium* protein export supports the notion that the PVM is a dynamic structure with selective permeability and trafficking of parasite proteins, including non-exported proteins, into the host cell cytoplasm (Matz, Beck et al. 2020, Gabriela, Matthews et al. 2022). Moreover, unconventional antigen processing in reticulocytes, which lack key peptide-editing components such as ERAP1 and Tapasin, potentially favouring the loading of atypical peptides on HLA-I molecules. Together, these findings suggest that leakage or translocation from the PVM and atypical processing pathways may underlie the presentation of cytoplasmic proteins observed in our study. Further mechanistic studies will be required to confirm these hypotheses

11. I think the authors need to do some clarification around immunogenic versus protective – as it's the latter that we desperately need to identify without being distracted by the former. Is there anything in their work, beyond the 2 tested and the iRBC lysis assay, that could advance some of the other targets further as protective antigens for inclusion in a vaccine? Will we have to test them one at a time? What is our best predictor to date?

We appreciate this important point regarding the distinction between immunogenicity and true protective efficacy. We made several modifications in the text to clarify this issue, which include: (i) changing the term "immunogenic/ immunogenicity per "antigenic/antigenicity" when describing and discussing the data obtained *ex vivo* upon stimulation with peptides (Figs. 1c-j; 2d-f,i-l; 3b,e,f,h-k; 4a,b); (ii) immunogenicity was used to describe the antigen-specific immune response elicited by immunization (Fig 4i-l); (iii) the term "protection" was exclusively associated with the immune response that led to iRetics-specific lysis *in vitro* or a reduction in parasitemia *in vivo* (Fig 4c-g,k,l).

In the current study, we provide immunogenic and protective evidence only for 2 tested ribosomal proteins (60S RP L29 and 40S RP S30), as demonstrated by both *in vitro Py*-iRetics lysis by antigen-specific $CD8^+$ T cells and *in vivo* parasitemia reduction following vaccination with the recombinant proteins. These findings support the potential of ribosomal proteins as protective antigens. Although only two RP candidates were evaluated for *in vivo* protection in the present work, our data suggest that additional RPs with similar immunological profiles may also serve as protective targets. Systematic testing of each antigen is ideal but not always feasible. Importantly, our integrated pipeline—including immunopeptidomics, cross-species conservation analyses, and functional $CD8^+$ T cell assays—provides a rational framework to prioritize antigens with the highest potential for protective $CD8^+$ T cell immunity. This combination currently represents our best predictor of protective potential even if restricted to iRBCs.

We agree that advancing few antigens into optimized vaccine formulations is a critical next step. Indeed, such studies are underway, employing antigens one-by-one or in bulk combinations. For this manuscript we focus on identifying antigens capable of eliciting broad, cross-stage, and cross-species $CD8^+$ T cell responses represent a significantly transformative leap forward for the field as it enables, for the first time, building a protective T cell-based vaccine.

Minor Points:

Line 59: "making inducing T cells a major challenge". It might be good from the start to clarify that it's not just T-cell induction, but induction of protective T-cell responses that is desired.

We thank the reviewer for this suggestion. The text has been revised accordingly and now highlights that the challenge is in inducing protective T cell responses, rather than T cell induction in general.

Throughout: There is a bit of inconsistency with the titling of ELIspot/ELISpot, I believe the correct abbreviation for the Enzyme-Linked Immunospot assay is ELISpot (capital S).

We thank the reviewer for pointing this out. The terminology has now been standardized throughout the manuscript, and all instances have been corrected to 'ELISpot' (capital S), the correct abbreviation for the Enzyme-Linked Immunospot assay.

Line 245: "NHP were infected with 2,500 cryopreserved P. knowlesi (Pk)". Presumably IV?

The text has been updated accordingly and now specifies that NHPs were infected intravenously (IV) with 2,500 cryopreserved *P. knowlesi* parasites (Line 346).

Figure 3: I found panels b and e particularly hard to interpret – is there an easier way of displaying this data?

We modified all the schematic panels for better interpretation.

Line 203: One protein is listed as coming from 50S RP3? Is this a mito- or apiocoplast ribosomal protein?

According to UniProt GO annotation Acession A5K3E2, it is a cytosolic small ribosomal subunit.

- All other minor corrections (e.g., figure clarifications, grammar, and nomenclature) have been addressed in the revised manuscript.

Reviewer #2 & #3

We thank Reviewers #2 and #3 for their enthusiastic support and detailed suggestions to improve clarity and impact.

• The Abstract states that most of the 166 different proteins identified by immunopeptidomic analysis were housekeeping proteins, and that many peptide sequences were highly conserved in P. falciparum and P. vivax. For clarity, the authors should specify exact numbers: how many non-house-keeping proteins were identified; how many non-house-keeping proteins were tested for antigenicity in P. vivax- and P. falciparum infected humans, or Plasmodium-infected/immunized primates (and what Plasmodium species); how many were tested for protection in mice, and what the results were.

We appreciate the reviewer's suggestion and have revised the Abstract and Results sections to include specific numbers for clarity. Of the 166 proteins identified by immunopeptidomic analysis, 75 proteins are known to be housekeeping, whereas the other 91 were either non-housekeeping or unknown proteins, now classified in SI. 3. It was synthetized 13 peptides of non-housekeeping proteins, among these, 11 were tested for antigenicity in samples from *Pv*- and *Pf*-infected humans, 13 were evaluated in non-human primates (NHPs) *Plasmodium* infection, and 11 for rodent malaria infection. None of the non-housekeeping proteins were evaluated for immunogenicity and protection in the current study. The summary of these finding is shown as SI. 7.

• The vast majority of identified peptides were derived from essential housekeeping proteins (line 409), and more than half from ribosomal proteins; but arguably use of housekeeping proteins as vaccine targets may

not be a biologically appropriate vaccine strategy. The authors should clarify and expand their presentation (in abstract and in main text) of findings with the non-housekeeping proteins as potential vaccine candidates; and justify how vaccine based on housekeeping genes could be developed into an effective vaccine for humans.

While these proteins are essential for parasite survival and not necessary surface-exposed, several studies have demonstrated that housekeeping proteins, including ribosomal and heat shock proteins, can elicit strong T cell responses and confer protection in different infectious disease models. For example, ribosomal proteins have been validated as vaccine candidates against *Mycobacterium tuberculosis*, where boosting BCG with ribosomal protein RplJ enhanced protective immunity (Kennedy, Johnson et al. 2018). Similarly, ribosomal proteins have been explored as DNA vaccine targets in *Leishmania* species, inducing Th1 responses and reducing lesion development in murine models (Ramirez, Corvo et al. 2014). Heat shock proteins, another class of housekeeping proteins, have also been widely studied as immunogenic antigens and adjuvants in vaccines for parasitic diseases (Corigliano, Sander et al. 2020).

Importantly, licensed bacterial vaccines also include housekeeping proteins as components. For instance, pneumococcal protein-based vaccines incorporate conserved cytoplasmic proteins such as pneumococcal surface protein A (PspA) and pneumolysin derivatives, which are essential for bacterial physiology yet highly immunogenic (Briles, Paton et al. 2019). Similarly, meningococcal vaccines include factor H binding protein (fHbp), a housekeeping protein critical for complement evasion (Yee, Barnes et al. 2023). These examples demonstrate that targeting conserved intracellular proteins can be biologically appropriate and effective.

Within the malaria context, the only bona fide CD8$^+$ T cell antigen identified to date is the ribosomal 60S subunit protein L6 (RPL6). This antigen was originally identified in mice infected exclusively with blood-stage *Plasmodium* parasites (Briles, Paton et al. 2019), yet it was subsequently shown to be the most potent liver-stage CD8$^+$ T cell target described so far (Valencia-Hernandez, Ng et al. 2020). These findings provide strong evidence that housekeeping proteins—despite their intracellular roles—can be naturally processed and presented to the immune system in a manner that elicits robust protective T-cell immunity. Based on this precedent, and given the high conservation and cross-stage expression of ribosomal proteins across *Plasmodium* species, we prioritized ribosomal antigens for further evaluation in this study

• The study does not explore CD4$^+$ T cell responses or HLA class II restricted epitopes, which are important for the induction and maintenance of CD8$^+$ T cell responses, for providing help for optimal antibody responses, and for coordinating broader long-lasting protective immunity. Given the importance of CD4$^+$ T cells in effective vaccine-induced protective immunity, the authors should clarify their rationale for focusing exclusively on HLA class I epitopes and address whether class II restricted responses were considered or assessed. This focus exclusively on CD8+ T cells seems to be a deficiency in an otherwise comprehensive study, especially since many longer peptides (10-16mers) were identified (line 103).

Our focus on HLA class I epitopes was driven by the biology of *Plasmodium vivax*-infected reticulocytes, which uniquely express HLA-I molecules and present antigens to CD8$^+$ T cells, as previously demonstrated by our group (Junqueira, Barbosa et al. 2018). The immunopeptidomic employed workflow specifically isolates peptides bound to HLA-I complexes, and the peptides identified were predominantly recognized by CD8$^+$ T cells in functional assays, confirming their relevance to cytotoxic responses. While many peptides were longer than canonical 8–10mers, this likely reflects unconventional processing in reticulocytes, which lack key trimming components such as ERAP1 and Tapasin, rather than HLA-II presentation.

We fully agree that CD4$^+$ T cells and HLA-II-restricted epitopes play a critical role in sustaining CD8$^+$ T cell responses and coordinating humoral immunity. However, these were outside the scope of the current study, which aimed to address a major knowledge gap regarding CD8$^+$ T cell targets in malaria. Similar immunopeptidome studies employing professional antigen-presenting T cells in the context of *Plasmodium* infection should contribute to the elucidation of HLA-II-restricted epitopes.

• The sample IDs in Figure 1a (e.g., 24, 26, 28) are not clearly explained. Manuscript text (line 94) states that "Patient samples were anonymized as 24, 26, 28, 30, 31, 34, and 35". This should, therefore, be stated explicitly in the figure legend to avoid confusion about potential experimental or temporal ordering. Suggested rewording: "Samples (labeled 24, 26, 28, 30, 31, 34, and 35) were anonymized identifiers assigned arbitrarily to each sample."

This has been modified in the figure 1a legend and highlighted in the SI. 1.

• In Figure 1a, the transition from All peptides to P. vivax–specific peptides is logical, but it is not explicitly stated that the per-sample counts shown (Total and Unique) represent only peptides matching the P. vivax Sal-1 proteome. Please clarify this in the figure legend to avoid confusion.

This has been modified in the figure 1a legend.

• Legend Figure 1c "indicated by colour intensity where light blue is the minimum and dark pink the maximum" is vague - minimum of what? It seems to refer to number of patients, which is already on the x-axis. The figure legend should be revised for clarity, e.g., to explicitly state that: (i) the x-axis represents the number of patients, (ii) the y-axis shows percent identity to P. falciparum orthologs, (iii) dot size reflects the level of sequence identity, and (iv) dot color ranges from light blue (minimum patients) to dark pink (maximum). Also, including a color bar and size scale legend within the figure would further enhance clarity for the reader.

This figure 1c has now been moved to Extended data figure 1c and composed with its related table 1d. The legend was modified following the reviewer suggestion.

• Figure 1d: The figure uses similar or almost identical colors to represent different groups, making it difficult to distinguish between them. It is unclear which percentage corresponds to which group. The color coding should be clarified to improve interpretability.

This figure now corresponds to figure 1b. It has been modified with different range of colors for a clearer interpretation.

• Figure 1 legend: the lettering that refers to individual panels is inconsistently formatted - sometimes following a comma, other times in parentheses. Please ensure consistent formatting of panel labels throughout the figure legend.

We reviewed the panel labels and modified where it was needed, so it follows the same formatting throughout all the figure legends.

• Figure 2a: The legend refers to continuous data representing individuals from 2 to 5, but the figure itself displays discrete values. This makes interpretation difficult - for example, it is unclear whether peptides shown in green are recognized by 2 or 3 individuals. Please ensure that the legend accurately reflects the discrete nature of the data.

The figure legend has been modified according to the reviewer suggestion. It now states clearly the color scheme according to the number of patients for whom peptides were predicted to bind to HLA-A,B or C alleles.

• Figure 2a-b includes only 5 of the 7 samples mentioned in the text. If the other 2 samples were excluded because they did not share peptides with any others, this should be explicitly stated in the figure legend or main text to avoid confusion.

We thank the Reviewer for drawing attention to this issue. One of the samples (24) indeed did not share any peptides predicted to bind to the HLA-ABC of the other samples and was therefore excluded from the analysis. In addition, due to a default display setting of the Intervene UpSet plot, unintentionally only five samples were shown in the original figure. We sincerely apologize for this. The figure has now been corrected to ensure appropriate sample representation, and the figure legend and main text have been revised to have a clearer description of this figure and state that only sample 24 was not included in this analysis

• Extended Data Figure 4: The legend appears to be incomplete, with panel d missing from the description. The text currently ends after the explanation of panels b. Please include a description of panel d to ensure the figure is fully interpretable.

This data is now represented in Extended data figure 6. The legend has been updated and contain a full description of all panels.

• Line 56 (and elsewhere, e.g. line 176): should be anti-Plasmodium T cell response, not anti-malaria T cell response; malaria is the disease.

This has been corrected in the manuscript.

• Line 93: please confirm that the "7 acute malaria patients" were confirmed to be infected with P. vivax, and not P. falciparum – and include details of parasite load and clinical details etc as a supplementary table.

The manuscript has been updated, stating that the donors were confirmed to be infected with *P. vivax*, and not *P. falciparum* with parasitemia ranging from 501-100,000 parasites/mm^3. Clinical details, as well as parasite load are included in SI. 1.

• Line 94: should refer to detailed HLA typing information of the subjects.

We thank the reviewer for this suggestion. While we agree that providing detailed HLA typing information is important, incorporating the full allele list directly into the main text disrupted the narrative flow. The detailed HLA typing of all subjects can be found as a figure in Extended Data Fig. 6a.

• Line 103: Include text noting that 8-10mers would be expected bot be class I restricted CD8+ T cell epitopes, whereas the longer 10-16mer peptides would likely be CD4+ T cell epitopes, and cite the relevant literature.

Our immunopeptidome experimental workflow is specifically designed to detect HLA class I–restricted peptides, which correspond to $CD8^+$ T cell epitopes. This is also consistent with the fact that reticulocytes do not express HLA class II molecules and therefore cannot present $CD4^+$ T cell epitopes (Junqueira, Barbosa et al. 2018).

Classical HLA class I molecules generally present peptides of 8–10 amino acids, recognized by $CD8^+$ T cells (Blum, Wearsch et al. 2013), but in a less extend, present peptides up to 15 mer (Trolle, McMurtrey et al. 2016) (reference included in the suggested line.). HLA class II molecules typically present longer peptides (10–16 amino acids) to $CD4^+$ T cells (Rudensky, Preston-Hurlburt et al. 1991, Blum, Wearsch et al. 2013). Reticulocytes lack HLA class II expression (Sieff, Bicknell et al. 1982), supporting the exclusivity of HLA-I presentation in our experimental system.

This led us to investigate whether the longer peptides observed could be presented by unconventional HLA molecules, such as HLA-E, which has been reported to bind longer peptides and play a role in immune modulation (Celik, Kraemer et al. 2016).

• Lines 108–111: The identity percentages stated in the text ("These proteins share 83% and 63% amino acid sequence identity…") do not appear to match the y-axis values shown in Figure 1c. For example, ETRAMP (A5K676) appears below 60% on the graph, and 40S S2 does not reach 97% as claimed. Please verify whether the values plotted on the y-axis are correct or if the numbers reported in the text need adjustment.

The percentage of identity for ETRAMP (A5K676) is 43% as described in the text and in the Extended figure 1b,c. 40S S2 representation in the figure has been updated.

• Conservation of identified peptides/proteins between all Plasmodium species should be clearly presented as a Table or Figure (or at least for the most relevant subset) – line 243. Currently, the focus is on conservation between Pv and Pf, but conservation in relation to P. knowlesi and P. yoelii is particularly relevant to the study design, and conservation across all species is relevant to the author's message of a cross-species vaccine.

We appreciate this suggestion and we expanded our homology analysis not only to other species (*P. falciparum, P. yoelii, P. bergheii, P. cynomolgi and P. Knowlesii*) but also different stages of the parasite. This can be found in the Supplementary information 3: Housekeeping and non-housekeeping peptide – Proteins homology across species.

• Line 126-129: clarify how may proteins were tested for liver-stage expression, and what the rationale was for selecting this subset. The statement "All genes that were tested were found to be expressed in Pf liver stages" lacks context.

We appreciate the reviewer's comment, and we have clarified this section in the revised manuscript. Specifically, we evaluated five representative housekeeping genes for liver-stage expression: 40S ribosomal proteins S25 and S30, 60S ribosomal proteins L29 and L13, and Histone H2A. These genes were selected as representative members of the major protein families most enriched in our immunopeptidomics dataset (40S RP, 60S RP, and histones), allowing us to test whether the highly abundant housekeeping proteins identified in blood-stage reticulocytes are also transcribed during liver infection. Furthermore, we also performed the same expression analysis in *Pv*-infected livers (Extended data fig. 2a). The results description was expanded to clarify number of antigens evaluated, as well as their relative expression in comparison to CSP and TRAP.

• Line 126 and Extended data Figure 1. The RT-qPCR validation of Pf transcript expression in infected humanized mouse liver is informative, but the absence of a clearly stated negative control raises concerns about specificity. Inclusion of a negative control, such as uninfected humanized liver tissue, would strengthen the interpretation of Pf-specific expression during the liver stage. If such a control was not used, this limitation should be acknowledged. Alternatively, if uninfected controls were tested but not shown, it should be stated that no expression was detected.

We thank the reviewer for raising this important point. In this experiment, RT-qPCR data were normalized to *Pf* 18S rRNA, rather than to uninfected humanized liver samples. Our goal was specifically to quantify the relative expression of *Pf*, and now *Pv*, transcripts during the liver stage; therefore, uninfected liver tissue—which does not contain *Pf*—would not provide meaningful normalization for parasite-derived genes of interest. For this reason, uninfected controls were not included in the expression analysis.

• Line 139/140: state how many Pv or Pf subjects were tested by IFNg ELISpot.

This information has now been added to the manuscript. The Brazilian cohort consists of *Pv*-infected patients (n=24), *Pf* (n=7), or healthy donors from malaria endemic (E-HD; n=15) and non-endemic (NE-HD; n=6), *Pv* symptomatic (SYN, n=8), *Pv* asymptomatic (ASY, n=20). We also included the n for the new cohorts from Mali (E-HD, n=7; *Pf*, n=5) and CHMI (n=6).

• Line 145**:** The authors report no difference in IFNγ-producing SFCs between Pv- and Pf-infected patients. However, Figure 1f appears to show a lower percentage of Pf-infected individuals responding to the peptide pool compared to Pv-infected individuals. This difference in responder frequency is not statistically evaluated or discussed. Could the authors clarify whether this difference was observed to be significant, and if so, comment on its potential biological relevance, for example, whether it reflects differences in immunodominance, antigen exposure, or host response dynamics between the two infections?

We thank the reviewer for this comment. Although Figure 1f (now Fig. 1c) visually suggests a lower responder frequency among *P. falciparum*–infected patients, formal statistical analysis did not identify a significant difference between the two groups. Given the absence of a statistically significant difference, our data are consistent with the possibility that *Pv* and *Pf* infections elicit comparable levels of immunodominance and antigen exposure to the peptides included in the pool.

• Line 159: The sentence is incomplete - "IFNγ in response to some of the peptides (Fig. 1e, f). In general, the…" ends abruptly. Please correct.

This has been corrected in the manuscript.

• The authors should explain why they selected the NetMHCpan program for epitope prediction algorithm in preference to other computational a=tools, and note any limitations, and what a threshold of 10% means.

We selected NetMHCpan for epitope prediction because it is one of the most widely validated and comprehensive algorithms for predicting peptide binding to HLA class I molecules across diverse alleles. Unlike allele-specific tools, NetMHCpan integrates both binding affinity and eluted ligand data, enabling accurate predictions even for rare or less-characterized HLA alleles (Jurtz, Paul et al. 2017). This approach is particularly relevant for our study, given the high HLA diversity in the Brazilian population and the need to assess multiple alleles simultaneously.

The 10% threshold refers to the percentile rank of predicted binding affinity compared to a large set of random natural peptides; peptides with a rank ≤10% are considered potential binders. It is a common practice in used immunopeptidomics workflows to select a minimum threshold to balance sensitivity and specificity (Purcell, Ramarathinam et al. 2019).

We acknowledge that computational predictions have limitations, including possible false positives and the inability to fully account for unconventional antigen processing observed in reticulocytes. Therefore, all peptides were experimentally validated for antigenicity in patient samples and animal models, ensuring biological relevance beyond in silico predictions**.**

• Lines 207–209: The sentence stating that "non-binder" peptides had significantly higher positive responses (55.2%) than "binder" peptides (32% reactivity) is confusing. It is unclear whether the percentages refer to the number of peptides eliciting positive ELISpot responses or to the magnitude of IFNγ responses (e.g., SFCs). If it refers to the proportion of peptides with positive responses, this should be clearly stated. Additionally, the statistical test used to compare these groups should be reported in the main text or figure legend to avoid misinterpretation.

We appreciate the reviewer's comment and agree that the original wording was unclear. We now demonstrate the analysis in 2 different graphs (Fig. 2f and 2g). Figure 2f shows that non-binder peptides had significantly higher magnitude of IFNγ responses than binder peptides. The statistical test utilised was Mann Whitney. It is stated at the end of the legend. Figure 2g displays the percentages in bar graph and refer specifically to the frequency of individuals who showed positive IFNγ ELISpot responses —within the "binder" and "non-binder" categories.

• Lines 210-218 and 220-240: The authors propose that the unexpected immunogenicity of "non-HLA-ABC binder" peptides may result from deficiencies in peptide quality control and trimming in Pv-infected reticulocytes (iRetics), due to the absence of ERAP1 and Tapasin. This hypothesis is further supported by subsequent experiments showing HLA-E expression on iRetics and reduced IFNγ production by $CD8^+$ T cells upon HLA-E blockade. Together, these findings suggest that some of the immunogenic peptides may be presented through non-classical HLA-E rather than classical HLA-ABC pathways. However, the connection between these two sections (lines 210–218 and 220–240) could be made more explicit to help the reader follow the progression from the observed responses to the mechanistic explanation.

We thank the reviewer for this helpful suggestion. We have now revised the manuscript to make the connection between these two sections explicit, clarifying the logical progression from the observed antigenicity of non-HLA-ABC binder peptides to the mechanistic hypothesis involving altered peptide processing and potential HLA-E–mediated presentation.

• Line 256: "In light of published data…" – these publications should be cited

The corresponding publication has been cited in the manuscript.

• Lines 256- 276: The lack of boosting in liver $CD8^+$ T cell responses to CSP (Fig. 3g,h) is attributed to a single animal with unchanged responses between immunizations. While this may be a contributing factor, it would be helpful if the authors could comment more broadly on why CSP-specific responses might fail to boost despite repeated sporozoite exposure, particularly given its relevance as a vaccine target.

We thank the reviewer for this comment. While one animal with unchanged CSP responses contributed to the lack of statistically significant boosting, limited recall expansion of CSP-specific $CD8^+$ T cells after repeated sporozoite exposure has been previously documented. In whole-sporozoite immunization models, secondary sporozoite exposure often produces minimal or no boosting of CSP-specific T cells, despite effective priming, in part because CSP is rapidly shed after hepatocyte invasion, reducing the duration of antigen presentation (Richie, Church et al. 2023).

Moreover, clinical and experimental vaccination studies show that CSP-specific responses frequently plateau after the initial priming immunization, with booster doses eliciting only modest or transient recall responses, even under repeated sporozoite exposure. These patterns are consistent with our observation of limited CSP boosting in the liver (Ali, Stockdale et al. 2025).

• Lines 293-295: The observation that VL9 blocking is not strictly peptide-dependent but varies between animals is intriguing. Further commentary on potential explanations (e.g., Mamu-E allelic differences, TCR repertoire diversity) would enrich interpretation.

We thank the reviewer for highlighting this point. We agree that the variable degree of VL9-mediated blocking across animals suggests biological heterogeneity in HLA-E/Mamu-E–restricted presentation. Several non-exclusive mechanisms may explain this observation. First, Mamu-E allelic variation among rhesus macaques can influence peptide-binding preferences and the stability of Mamu-E–peptide complexes, potentially altering the extent to which VL9 competes with presented ligands. Second, differences in the $CD8^+$ T cell repertoire between animals, including variation in TCR affinity, breadth, and clonal dominance, may shape the sensitivity of antigen-specific T cells to competitive blockade. Finally, variation in antigen processing or peptide supply during infection may alter which peptides are preferentially loaded onto Mamu-E, contributing to animal-specific blocking outcomes.

• Lines 328-339: The final experiment showing protection upon immunization with recombinant RP L29 or S30 strengthens the translational value of these targets. However, the authors should clarify whether the observed protection reflects short-term effector T cell responses or involves memory T cell induction, as this has implications for vaccine durability and design.

We appreciate the reviewer's question regarding whether the observed protection reflects short-term effector $CD8^+$ T cell responses or the induction of long-lived memory T cells, as this distinction is indeed critical for vaccine durability and design. In a parallel work, our group has begun evaluating memory T cell responses elicited by ribosomal protein–based immunization, including assessments of central and effector memory subsets. These preliminary data indicate that memory T cell induction does occur; however, these studies extend beyond the scope of the present manuscript, which focuses on identifying protective $CD8^+$ T cell targets and establishing a proof-of-concept for their protective capacity. We also note that memory formation is strongly influenced by adjuvant choice—memory is as much a feature of the adjuvant as of the antigen itself. Accordingly, future studies will incorporate more vaccine-relevant adjuvants to better model clinically applicable immune responses and to refine antigen prioritization based on their ability to induce durable protection. We fully agree that evaluating the longevity and quality of the memory response is an essential next step in advancing these antigens toward vaccine development.

Reviewer #4 & #5

We appreciate the reviewers' recognition of the value of our immunopeptidomic approach and the potential of our findings to inform future malaria vaccine design.

Major points

• As a general concern, the data are overinterpreted in many parts of the text including the Abstract and the Discussion. Some P values are very close to 0.05 and in certain examples, the authors generalize data across groups of samples or animals despite extensive variability. The statement in the Abstract that the epitopes were validated in Pv- and Pf-infected samples is an overstatement. This was done with a small number of donors and only from one geographical/epidemiological area. As another example of an overstatement, Line 85: "These epitopes were immunogenic in Pv- and Pf-infected patients, P. cynomolgi (Pcy)- and P. knowlesi (Pk)-infected non-human primates (NHP) and P. yoelii (Py)-infected mice".

We appreciate the reviewer's concern regarding overinterpretation and have addressed this by revising the Abstract and Discussion to ensure statements reflect the scope of our data. Specifically, we have clarified that initial validation of antigenicity was performed in a limited cohort from Brazil and have now expanded the study to include samples from two additional endemic regions: Mali (*P. falciparum*) and a blood-stage *P. vivax* Controlled Human Malaria Infection (CHMI) in the US. These new data confirm the antigenicity of several candidate peptides across distinct geographical and epidemiological settings, strengthening the generalizability of our findings (Fig 1i,j).

• The authors state >99% infected reticulocytes, but there is no mention of how uninfected reticulocytes or RBC contamination was ruled out. This could confound the peptide repertoire observed.

We confirm that the purity of infected reticulocyte preparations was verified for each sample by Giemsa-stained smear microscopy, which showed >99% infected reticulocytes and minimum contamination with uninfected reticulocytes or mature RBCs. This step ensured that the immunopeptidomic analysis reflected peptides presented by infected reticulocytes only. Moreover, mature RBCs do not express HLA molecules, therefore not interfering within the peptide repertoire. To further support this, we are adding an image of a representative smear as an example bellow.

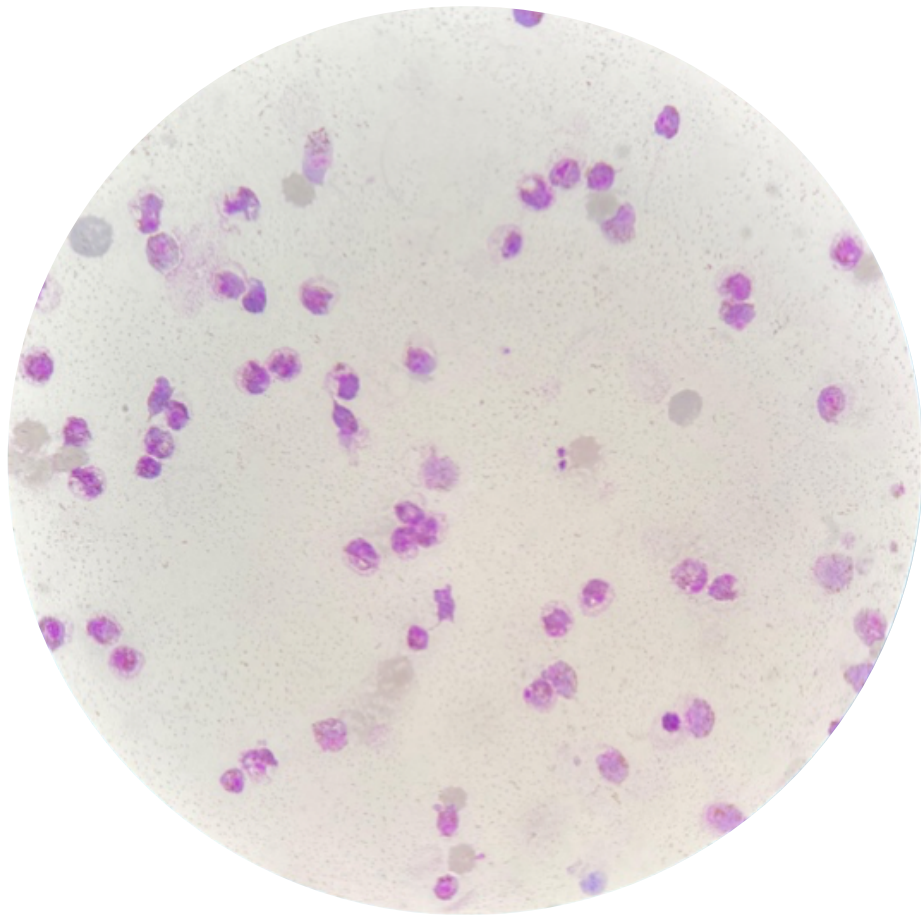

Rebuttal letter Figure 1. Representative smear showing highly purified *Pv*-infected reticulocytes.

• In the data from the immunopeptidomic analysis, there seems to be a bias where sample 35 yielded excessively more peptides than the other samples. This is not discussed.

Peptide yield variability is widely recognized in the field and does not necessarily indicate biological differences. The higher number of identified peptides in this sample is most likely due to technical factors inherent to immunopeptidomic workflows rather than biological bias. For example, the immunoaffinity capture can vary between samples due to differences in antibody binding or bead performance (Pandey, Ramarathinam et al. 2021). If the sample 35 had more efficient recovery of HLA complexes, this would directly increase peptide yield.

A large proportion of identified peptides are derived from housekeeping proteins, such as ribosomal subunits and histones. While the authors showed that these are highly conserved and expressed across parasite stages, their abundance and essential nature raise the possibility that responses may reflect immunodominance due to abundance rather than physiological relevance or protective efficacy. This should be discussed more in the context of antigen selection for vaccines.

We agree that many identified peptides originate from housekeeping proteins such as ribosomal subunits and histones. This reflects the unbiased nature of the immunopeptidomic pipeline, which captures the most abundantly presented peptides on HLA molecules. While abundance may contribute to immunodominance, our antigen selection was not based solely on frequency. We performed extensive functional validation, including IFNγ ELISpot and intracellular cytokine staining with PBMCs from *P. vivax* and *P. falciparum* patients, non-human primate studies during infection and liver-directed immunization, and murine challenge models. These assays demonstrated that several of these antigens elicit strong $CD8^+$ T cell responses and confer protective efficacy, including reduced parasitemia after adoptive transfer and active immunization with recombinant proteins. Thus, these antigens are not only abundant but also physiologically relevant and protective, supporting their consideration for vaccine development.

• The central conclusion that these epitopes could support a "universal malaria vaccine" is premature, especially in the absence of direct evidence of protection in liver-stage models or human challenge studies. The in vivo killing assays in mice are informative but limited in scope and species relevance.

Our conclusion is based on evidence that these antigens are expressed across parasite stages and species and are antigenic in multiple systems. Importantly, we provide direct evidence of liver-stage immunity in non-human primates: repeated *P. knowlesi* sporozoite infections and chemoprophylaxis vaccination significantly boosted $CD8^+$ T cell responses to our antigen pool in both blood and liver compartments (Fig. 3g–k), a hallmark of liver-directed protection. Furthermore, we have expanded the study to include a controlled human malaria infection (CHMI) with *P. vivax* blood-stage, where several of our antigen

candidates elicited measurable CD8+ T cell responses in volunteers post-challenge, confirming their antigenicity in humans (Fig 1j).

The ELISPOT data in Fig. 1e do not show very strong statistical significance. For the Pv-infected patients, there seems to be a major population that does not respond and a minor population that does. I think control should be an irrelevant peptide or one that does not meet their criteria for selection.

We thank the reviewer for this observation. The apparent lack of strong statistical significance is driven by heterogeneity in the magnitude of T cell responses among *P. vivax*–infected donors. As shown in the heat map in Fig. 1d, most donors exhibit positive but relatively low-magnitude ELISpot responses, while a smaller subset shows very strong responses. This distribution results in higher variability and therefore wider confidence intervals, despite the majority of donors responding above background.

Regarding the choice of control, we used DMSO-only stimulated cells as the standard background control for *ex vivo* human ELISpot assays, enabling us to detect antigen-specific IFN-γ production relative to each donor's baseline.

• The sample size for the Pf-infected PBMCs used for ELISPOT analysis was very small and differences were not significant compared to endemic controls. The experiments should be extended with a larger sample size given the importance of identifying cross-species peptides that elicit T cell responses.

We acknowledge the limited number of *P. falciparum* (Pf)-infected patients' samples in our ELISpot analysis. This constraint reflects the epidemiological reality in Brazil, where Pf malaria cases have markedly declined over the past decade, now representing only ~15% of infections and concentrated in remote Amazon regions (Garcia, Rodovalho et al. 2024). Despite this limitation, to strengthen our conclusions, we expanded the study by including Pf samples from Mali, a high-transmission setting. These additional experiments confirmed the antigenicity of several cross-species antigens in Pf-infected individuals, supporting their relevance beyond the Brazilian context (Fig. 1i).

• The responses in Fig. 1f are much lower in the Pv symptomatic and asymptomatic cohorts compared to the samples included in Fig. 1e. It is not clear whether these are entirely different samples, and the authors should provide an interpretation of these findings. Also, their result that there is no difference between symptomatic and asymptomatic patients in terms of responses to peptides calls into question whether they could play a role in protection.

We believe the reviewer is referring to current figure 1h, which shows data from the asymptomatic cohort. These samples were indeed different from those in figure 1c,d, and the number of samples tested in Fig. 1c,d was much higher (n=24) compared to Fig. 1i (n=8). Additionally, in figure. 1c, a small subset of donors exhibited very high IFNγ responses (>4000 SFC/million PBMC), which increased the overall mean.

Regarding the observation that responses were similar between symptomatic and asymptomatic individuals, we have addressed this in the discussion: the ability of asymptomatic patients to mount IFNγ responses to these peptides suggests that these antigens may contribute to controlling parasitemia and preventing clinical symptoms, supporting their potential role in protection.

• The immunological characterization of CD8+ T cell responses lacks critical functional depth. The authors assess IFN-γ production but do not examine T cell polyfunctionality (e.g., TNF, IL-2) or cytotoxic capacity (e.g., granzyme B, perforin, CD107a). Without these markers, it is difficult to assess whether these CD8+ T cells are functionally relevant or protective.

As suggested, we expanded our immunological characterization of CD8+ T cells beyond IFNγ, including functional markers upon stimulation with Total peptide pool (Fig 1g). Specifically, we assessed CD8+ T cell activation by markers (CD69), IL-2 production as indicators of effector functionality, and its cytotoxic

potential by degranulation (CD107a). These results strengthen the conclusion that the identified antigens can elicit effector $CD8^+$ T cell responses.

For broader impact, it is important to show responsiveness to these peptides in another population. As HLA restriction is central to $CD8^+$ T cell epitope presentation, the limited HLA diversity in this cohort restricts the translational relevance of the findings.

We agree that broader HLA diversity is important for translational relevance. To address this and other referee's suggestions, we expanded our study to include cohorts from two distinct geographic regions—Mali and US—in addition to Brazil. The results in these populations with different genetic background corroborated our initial findings: most peptides identified and tested in the Brazilian cohort also showed an antigenic profile in these new populations. (Fig. 1i-j) This strengthens the evidence that the selected antigens are broadly recognized across diverse genetic backgrounds and supports their potential for inclusion in a cross-species malaria vaccine strategy.

• The apparent disconnect between predicted peptide–MHC binding and observed T cell responses is not addressed mechanistically. In fact, several peptides categorized as "non-binders" elicited stronger responses than "binders," which raises questions about whether these are genuinely MHC-I–restricted $CD8^+$ T cell responses or due to alternative mechanisms (e.g., HLA-E presentation, innate activation, or off-target effects). This challenges the current understanding of MHC class I peptide selection and suggests either non-classical presentation or assay artifacts. While the authors suggest that the responses may be mediated by non-classical HLA-E presentation (Fig. 2h-j), this is not experimentally confirmed beyond VL9 peptide blocking assays, making the HLA-E restriction claim speculative. Other assays could be considered to validate this claim.

We appreciate this comment, which was also raised by other reviewers, and agree that additional validation was warranted. In response, we performed an *in vitro* assay using a K562 cell line engineered by CRISPR to lack classical HLA-I expression, while overexpress of HLA-E*01:03 or not. These cells were pulsed with the Total pool (all 50 synthesized peptides) and co-cultured with primed $CD8^+$ T cells isolated from *Pv*–infected patients. $CD8^+$ T cell activation was assessed by IFN-γ ELISpot. As shown in figure 2l, $CD8^+$ T cells co-cultured with Total pool–pulsed K562 HLA-E*01:03 cells produced significantly higher levels of IFN-γ compared to those co-cultured with Total pool–pulsed K562 $HLA^{-/-}$ cells. These results provide direct experimental evidence that a subset of responses previously attributed to "non-binder" peptides is in fact mediated by non-classical HLA-E presentation, thereby supporting the mechanistic basis of our initial hypothesis.

• Line 285: the authors state that all animals produced anti-CSP IgG but one of them only produced low antibodies after the third vaccination.

Thank you for noting this discrepancy. The statement regarding anti-CSP IgG responses has been corrected in the results section of the revised manuscript to accurately reflect the data.

• For the murine model, the specificity of the cell killing for the peptides from their dataset would be stronger if they showed that one of the non-stimulatory peptides had no killing effect in this assay.

We appreciate the reviewer's insightful comment. To address this point, we included an additional control using peptide S25.1, which was identified as non-antigenic in our murine model. $CD8^+$ T cells were stimulated with S25.1 and subsequently evaluated in the killing assay. As shown in the updated figure 4e, S25.1-stimulated $CD8^+$ T cells exhibited only minimal killing activity, comparable to the unstimulated control. Although a small but statistically significant difference was observed at the 5:1 effector-to-target ratio relative to naïve $CD8^+$ T cells, the overall killing remained low and clearly distinct from the robust

cytotoxicity induced by the antigenic peptides. These results strengthen the conclusion that the killing effect observed in our assay is specific to the stimulatory peptides identified in our dataset.

• The Discussion fails to discuss the potential advantages of this approach compared to whole parasite Pf or Pv vaccines, such as PfSPZ or PvSPZ. If those epitopes are expressed by sporozoites and conserved, then a whole parasite vaccine would present these epitopes and contribute to cross-stage immunity. Generally, the Discussion mostly reiterates the results and does not provide enough discussion of the literature and the broader implications of these findings for vaccine design.

We appreciate this comment and have revised the discussion to include a comparison of our approach with whole-parasite vaccines such as PfSPZ and PvSPZ. Whole-parasite vaccines can present a broad repertoire of antigens, including those identified in our study, and have demonstrated strong protection in controlled human malaria infection (CHMI) models. However, they face significant challenges, including complex manufacturing, requirement for cryopreservation and intravenous administration, high cost, and logistical hurdles for large-scale deployment in endemic regions (Richie, Church et al. 2023, Moita and Prudencio 2024).

In contrast, epitope- or protein-based vaccines, which was the focus of this study, offer several advantages, such as: simpler production and stability; safety and regulatory feasibility; flexibility for multistage and multivalent design; potential for combination with other platforms (e.g., viral vectors, mRNA) to enhance immunogenicity and durability (Duffy, Gorres et al. 2024, Chen, Wang et al. 2025).

Minor points

• Ext data table 1 shows homology, not identity. This contradicts with the text.

This has been corrected in the text and in the figures and legends.

• Transcriptomic databases do not necessarily reflect protein expression at the other life cycle stages.

We agree that transcriptomic datasets do not necessarily reflect protein expression across life-cycle stages. The text has been revised throughout the manuscript to clarify that transcriptomic data indicate transcriptional evidence only and do not directly infer protein abundance or expression.

• Are the peptides that elicit the strongest responses across species also conserved in other organisms (eg identity to human sequences, other pathogens?)

We thank the reviewer for this comment. To avoid the inclusion of peptides with potential homology to human proteins, our peptide-identification pipeline was designed to retain only parasite-derived peptides that differed from the human proteome by a minimum of two amino acids. Therefore, peptides with high identity to human sequences were explicitly excluded from the analysis.

We thank all reviewers again for their valuable feedback. We hope the revised manuscript and this rebuttal letter address all concerns satisfactorily, and that the manuscript is now suitable for publication.

References

Ali, M. S., L. Stockdale, I. Sagara, I. Zongo, R. S. Yerbanga, A. Mahamar, F. Nikiema, A. Tapily, F. Sompougdou, M. Diarra, D. Bellamy, S. Provstgaard-Morys, C. Zoungrana, D. Issiaka, A. Haro, K. Sanogo, A. A. Sienou, M. Kaya, S. Traore, O. M. Dicko, Y. Kone, H. Yalcouye, I. Thera, K. Diarra, P. Snell, O. Ofori-Anyinam, C. Ockenhouse, C. Lee, K. Ewer, H. Tinto, A. Djimde, J. B. Ouedraogo, A. Dicko, D. Chandramohan and B. Greenwood (2025). "The anti-circumsporozoite antibody response to repeated, seasonal booster doses of the malaria vaccine RTS,S/AS01(E)." NPJ Vaccines **10**(1): 26.

Blum, J. S., P. A. Wearsch and P. Cresswell (2013). "Pathways of antigen processing." Annu Rev Immunol **31**: 443-473.

Briles, D. E., J. C. Paton, R. Mukerji, E. Swiatlo and M. J. Crain (2019). "Pneumococcal Vaccines." Microbiol Spectr **7**(6).

Celik, A. A., T. Kraemer, T. Huyton, R. Blasczyk and C. Bade-Doding (2016). "The diversity of the HLA-E-restricted peptide repertoire explains the immunological impact of the Arg107Gly mismatch." Immunogenetics **68**(1): 29-41.

Chen, J., Q. Wang, X. He and B. Yang (2025). "Malaria Vaccines: Current Achievements and Path Forward." Vaccines (Basel) **13**(5).

Corigliano, M. G., V. A. Sander, E. F. Sanchez Lopez, V. A. Ramos Duarte, L. F. Mendoza Morales, S. O. Angel and M. Clemente (2020). "Heat Shock Proteins 90 kDa: Immunomodulators and Adjuvants in Vaccine Design Against Infectious Diseases." Front Bioeng Biotechnol **8**: 622186.

Douglas, A. D., G. C. Baldeviano, C. M. Lucas, L. A. Lugo-Roman, C. Crosnier, S. J. Bartholdson, A. Diouf, K. Miura, L. E. Lambert, J. A. Ventocilla, K. P. Leiva, K. H. Milne, J. J. Illingworth, A. J. Spencer, K. A. Hjerrild, D. G. Alanine, A. V. Turner, J. T. Moorhead, K. A. Edgel, Y. Wu, C. A. Long, G. J. Wright, A. G. Lescano and S. J. Draper (2015). "A PfRH5-based vaccine is efficacious against heterologous strain blood-stage Plasmodium falciparum infection in aotus monkeys." Cell Host Microbe **17**(1): 130-139.

Duffy, P. E., J. P. Gorres, S. A. Healy and M. Fried (2024). "Malaria vaccines: a new era of prevention and control." Nat Rev Microbiol **22**(12): 756-772.

Gabriela, M., K. M. Matthews, C. Boshoven, B. Kouskousis, T. K. Jonsdottir, H. E. Bullen, J. Modak, D. L. Steer, B. E. Sleebs, B. S. Crabb, T. F. de Koning-Ward and P. R. Gilson (2022). "A revised mechanism for how Plasmodium falciparum recruits and exports proteins into its erythrocytic host cell." PLoS Pathog **18**(2): e1009977.

Garcia, K. K. S., S. R. Rodovalho and A. M. Siqueira (2024). "Towards malaria elimination: a reflection about digital notification modules to improve malaria cases notification speed and follow-up in the Brazilian Amazon region." Malar J **23**(1): 162.

Ilani, P., P. B. Nyarko, A. Camara, L. N. Amenga-Etego and Y. Aniweh (2025). "PfRH5 vaccine; from the bench to the vial." NPJ Vaccines **10**(1): 82.

Junqueira, C., C. R. R. Barbosa, P. A. C. Costa, A. Teixeira-Carvalho, G. Castro, S. Sen Santara, R. P. Barbosa, F. Dotiwala, D. B. Pereira, L. R. Antonelli, J. Lieberman and R. T. Gazzinelli (2018). "Cytotoxic CD8(+) T cells recognize and kill Plasmodium vivax-infected reticulocytes." Nat Med **24**(9): 1330-1336.

Jurtz, V., S. Paul, M. Andreatta, P. Marcatili, B. Peters and M. Nielsen (2017). "NetMHCpan-4.0: Improved Peptide-MHC Class I Interaction Predictions Integrating Eluted Ligand and Peptide Binding Affinity Data." J Immunol **199**(9): 3360-3368.

Kennedy, S. C., A. J. Johnson, S. Bharrhan, C. S. Lindestam Arlehamn, J. Xu, S. J. Garforth, J. Chan, W. R. Jacobs, Jr., A. Sette, S. C. Almo and S. A. Porcelli (2018). "Identification of Mycobacterial Ribosomal Proteins as Targets for CD4(+) T Cells That Enhance Protective Immunity in Tuberculosis." Infect Immun **86**(9).

Matz, J. M., J. R. Beck and M. J. Blackman (2020). "The parasitophorous vacuole of the blood-stage malaria parasite." Nat Rev Microbiol **18**(7): 379-391.

Minassian, A. M., S. E. Silk, J. R. Barrett, C. M. Nielsen, K. Miura, A. Diouf, C. Loos, J. K. Fallon, A. R. Michell, M. T. White, N. J. Edwards, I. D. Poulton, C. H. Mitton, R. O. Payne, M. Marks, H. Maxwell-Scott, A. Querol-Rubiera, K. Bisnauthsing, R. Batra, T. Ogrina, N. J. Brendish, Y. Themistocleous, T. A. Rawlinson, K. J. Ellis, D. Quinkert, M. Baker, R. Lopez Ramon, F. Ramos Lopez, L. Barfod, P. M. Folegatti, D. Silman, M. Datoo, I. J. Taylor, J. Jin, D. Pulido, A. D. Douglas, W. A. de Jongh, R. Smith, E. Berrie, A. R. Noe, C. L. Diggs, L. A. Soisson, R. Ashfield, S. N. Faust, A. L. Goodman, A. M. Lawrie, F. L. Nugent, G. Alter, C. A. Long and S. J. Draper (2021). "Reduced blood-stage malaria growth and immune correlates in humans following RH5 vaccination." Med **2**(6): 701-719 e719.

Moita, D. and M. Prudencio (2024). "Whole-sporozoite malaria vaccines: where we are, where we are going." EMBO Mol Med **16**(10): 2279-2289.

Murphy, S. C., G. A. Deye, B. K. L. Sim, S. Galbiati, J. K. Kennedy, K. W. Cohen, S. Chakravarty, N. Kc, Y. Abebe, E. R. James, J. G. Kublin, S. L. Hoffman, T. L. Richie and L. A. Jackson (2021). "PfSPZ-CVac efficacy against malaria increases from 0% to 75% when administered in the absence of erythrocyte stage parasitemia: A randomized, placebo-controlled trial with controlled human malaria infection." PLoS Pathog **17**(5): e1009594.

Pandey, K., S. H. Ramarathinam and A. W. Purcell (2021). "Isolation of HLA Bound Peptides by Immunoaffinity Capture and Identification by Mass Spectrometry." Curr Protoc **1**(3): e92.

Purcell, A. W., S. H. Ramarathinam and N. Ternette (2019). "Mass spectrometry-based identification of MHC-bound peptides for immunopeptidomics." Nat Protoc **14**(6): 1687-1707.

Ramirez, L., L. Corvo, M. C. Duarte, M. A. Chavez-Fumagalli, D. G. Valadares, D. M. Santos, C. I. de Oliveira, M. R. Escutia, C. Alonso, P. Bonay, C. A. Tavares, E. A. Coelho and M. Soto (2014). "Cross-protective effect of a combined L5 plus L3 Leishmania major ribosomal protein based vaccine combined with a Th1 adjuvant in murine cutaneous and visceral leishmaniasis." Parasit Vectors **7**: 3.

Richie, T. L., L. W. P. Church, T. Murshedkar, P. F. Billingsley, E. R. James, M. C. Chen, Y. Abebe, C. N. K, S. Chakravarty, D. Dolberg, S. A. Healy, H. Diawara, M. S. Sissoko, I. Sagara, D. M. Cook, J. E. Epstein, B. Mordmuller, M. Kapulu, A. Kreidenweiss, B. Franke-Fayard, S. T. Agnandji, M. A. Lopez Mikue, M. B. B. McCall, L. Steinhardt, M. Oneko, A. Olotu, A. M. Vaughan, J. G. Kublin, S. C. Murphy, S. Jongo, M. Tanner, S. B. Sirima, M. B. Laurens, C. Daubenberger, J. C. Silva, K. E. Lyke, C. J. Janse, M. Roestenberg, R. W. Sauerwein, S. Abdulla, A. Dicko, S. H. I. Kappe, B. K. L. Sim, P. E. Duffy, P. G. Kremsner and S. L. Hoffman (2023). "Sporozoite immunization: innovative translational science to support the fight against malaria." Expert Rev Vaccines **22**(1): 964-1007.

Rudensky, Y., P. Preston-Hurlburt, S. C. Hong, A. Barlow and C. A. Janeway, Jr. (1991). "Sequence analysis of peptides bound to MHC class II molecules." Nature **353**(6345): 622-627.

Sieff, C., D. Bicknell, G. Caine, J. Robinson, G. Lam and M. F. Greaves (1982). "Changes in cell surface antigen expression during hemopoietic differentiation." Blood **60**(3): 703-713.

Trolle, T., C. P. McMurtrey, J. Sidney, W. Bardet, S. C. Osborn, T. Kaever, A. Sette, W. H. Hildebrand, M. Nielsen and B. Peters (2016). "The Length Distribution of Class I-Restricted T Cell Epitopes Is Determined by Both Peptide Supply and MHC Allele-Specific Binding Preference." J Immunol **196**(4): 1480-1487.

Uhlen, M., L. Fagerberg, B. M. Hallstrom, C. Lindskog, P. Oksvold, A. Mardinoglu, A. Sivertsson, C. Kampf, E. Sjostedt, A. Asplund, I. Olsson, K. Edlund, E. Lundberg, S. Navani, C. A. Szigyarto, J. Odeberg, D. Djureinovic, J. O. Takanen, S. Hober, T. Alm, P. H. Edqvist, H. Berling, H. Tegel, J. Mulder, J. Rockberg, P. Nilsson, J. M. Schwenk, M. Hamsten, K. von Feilitzen, M. Forsberg, L. Persson, F. Johansson, M. Zwahlen, G. von Heijne, J. Nielsen and F. Ponten (2015). "Proteomics. Tissue-based map of the human proteome." Science **347**(6220): 1260419.

Valencia-Hernandez, A. M., W. Y. Ng, N. Ghazanfari, S. Ghilas, M. N. de Menezes, L. E. Holz, C. Huang, K. English, M. Naung, P. S. Tan, K. M. Tullett, T. M. Steiner, M. H. Enders, L. Beattie, Y. C. Chua, C. M.

Jones, A. Cozijnsen, V. Mollard, Y. Cai, D. G. Bowen, A. W. Purcell, N. L. La Gruta, J. A. Villadangos, T. de Koning-Ward, A. E. Barry, W. Barchet, I. A. Cockburn, G. I. McFadden, S. Gras, M. H. Lahoud, P. Bertolino, R. B. Schittenhelm, I. Caminschi, W. R. Heath and D. Fernandez-Ruiz (2020). "A Natural Peptide Antigen within the Plasmodium Ribosomal Protein RPL6 Confers Liver T(RM) Cell-Mediated Immunity against Malaria in Mice." Cell Host Microbe **27**(6): 950-962 e957.

Yee, W. X., G. Barnes, H. Lavender and C. M. Tang (2023). "Meningococcal factor H-binding protein: implications for disease susceptibility, virulence, and vaccines." Trends Microbiol **31**(8): 805-815.

Rebuttal Letter

We thank the reviewers for their contributions to improving the quality and clarity of the manuscript and for acknowledging our input in compiling high-quality data.

Reviewers #1, 4, and 5

We sincerely appreciate reviewers 1, 4, and 5 for their very positive comments and for recognizing the quality of our work and its novelty.

Reviewers #2, 3

I commend the authors for an informative, comprehensive and well written manuscript. The authors have expanded the originally submitted study to comprehensively address most of the concerns raised upon peer review, although there are two areas considered out-of-scope that would strengthen the manuscript:

We thank reviewers 2 and 3 for their thoughtful and constructive comments

Reviewer 1: Head-to-head study with RPL6 - a single (repeated) protection study comparing RPL6 against the novel CD8 antigen leads would have been highly informative, and (in my opinion) not a lot of extra work given the comprehensiveness of the current study.

As mentioned in our previous revision, in our hands, the rodent malaria sporozoite challenge is not well established; therefore, a head-to-head study with RPL6 is not feasible at the moment.

Reviewer 2/3: $CD4^{+}$ T cell responses - although the experimental strategy focused on HLA class I elution, and class II immunopeptidomic studies would be outside the scope of the current manuscript, which focuses on identification of novel targets of CD8+ T cell immune responses, the authors could nonetheless have assessed their novel vaccine targets for CD4 T cell responses in vitro, and could also have conducted the in silico HLA-class II peptide binding studies, to provide some consideration to this important cell type.

In fact, we evaluated the $CD4^{+}$ T cell response in samples from Pv- and Pf-infected individuals. As shown in figures 1f and 2j, the peptides did not elicit much $CD4^{+}$ T cell response. For this reason, we did not explore this type of response in more detail. Regarding the in silico HLA-II prediction, in fact, we could perform it. However, since the available algorithms for HLA-II prediction are less reliable than those for HLA-I, they require further experimental validation. Accordingly, a simple in silico prediction without biological validation would yield no valuable information.

Reviewer 2/3 #Lines 328-339: whether protection reflects short-term effector T cell responses or involves memory T cell induction - OK, although a challenge study conducted at 90 days post immunization using a well-established T cell-inducing adjuvant could have (at least in part) addressed this issue.

We agree that further evaluations should be conducted to better characterize the potential of the suggested vaccine formulation. In the current manuscript, we introduce the two vaccine formulations as a proof-of-concept of the potential of the newly identified antigens as vaccine candidates. Since the development of a final vaccine formulation was not within the scope of this project, we understand that this type of analysis should be explored in follow-up work.
