# Supplementary material for: Identification of cross-stage, cross-species malaria CD8+ T cell antigens
Source: Nature. 2026 Jul 1;655(8123):747–57. doi: 10.1038/s41586-026-10730-1 (PMC13661247; doi:10.1038/s41586-026-10730-1)
Supplement: Supplementary file 3 [file 41586_2026_10730_MOESM5_ESM.pdf]

SOURCE DATA

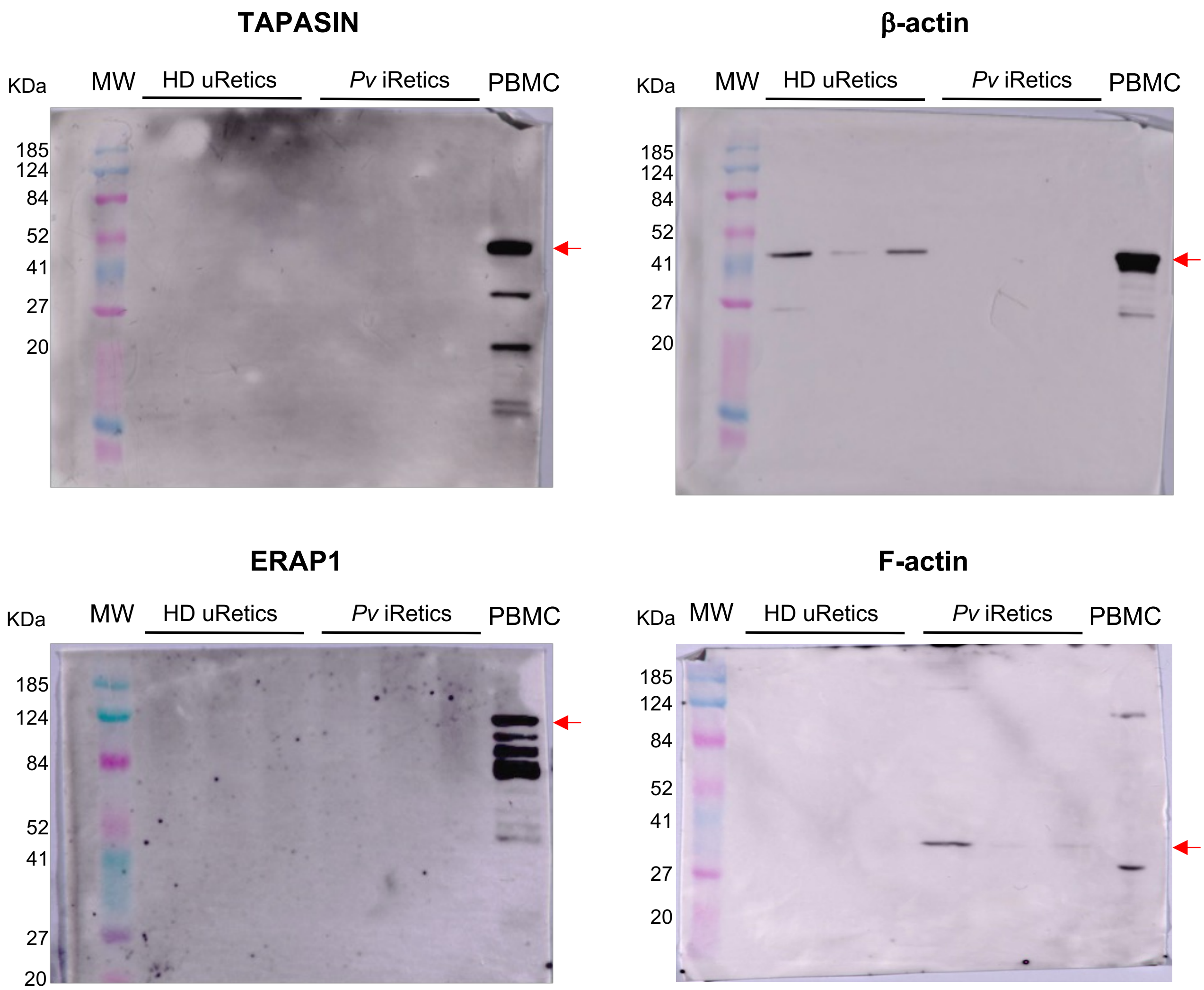

EXTENDED DATA 5a | Full gel Western Blots anti-TAPASIN (top-left), anti-ERAP1 (bottom-left), and loading controls anti- β-actin (top-right), anti-F-actin (bottom-right). One 12% SDS PAGE gel (top), and two mirrored 10% SDS PAGE gels (botton), and were run simultaneously with equivalent sample lysates: uninfected reticulocytes (uRetics) from 3 healthy donors (HD), *Pv*-infected reticulocytes (iRetics) from 3 patients. The gels were simultaneously transferred to nitrocellulose membranes for respective blotting. Anti-β-actin blotting was performed after stripping of the anti-TAPASIN antibody from the membrane.
