# Supplementary material for: Identification of cross-stage, cross-species malaria CD8+ T cell antigens
Source: Nature. 2026 Jul 1;655(8123):747–57. doi: 10.1038/s41586-026-10730-1 (PMC13661247; doi:10.1038/s41586-026-10730-1)
Supplement: Supplementary file 4 [file 41586_2026_10730_MOESM1_ESM.zip › 2025-05-11433B-s1/Barbosa_et_al_Supplementary_Information_Guide.docx]

**SUPPLEMENTARY INFORMATION (SI) GUIDE**

Supplementary information 1⎥ Clinical information

Supplementary information 2⎥ Identified peptides

Supplementary information 3⎥ Housekeeping and non-housekeeping peptide – Proteins homology across	species

Supplementary information 4⎥ *Plasmodium vivax* orthologous gene expression in parasite life cycle.

Supplementary information 5⎥ Selected peptides used for *in vitro* stimulation

Supplementary information 6⎥ Spectral match validation

Supplementary information 7⎥ HLA-ABC binding prediction

Supplementary information 8⎥ Summary of peptide antigenicity across *Plasmodium* species

Supplementary information 9⎥ Reagent list

Supplementary information 10⎥ Flow cytometry gating strategies
