# Supplementary material for: Identification of cross-stage, cross-species malaria CD8+ T cell antigens
Source: Nature. 2026 Jul 1;655(8123):747–57. doi: 10.1038/s41586-026-10730-1 (PMC13661247; doi:10.1038/s41586-026-10730-1)
Supplement: Supplementary file 4 [file 41586_2026_10730_MOESM1_ESM.zip › 2025-05-11433B-s1/Supplementary_Information_10.pdf]

Supplementary information 10 | Flow cytometry gating strategies

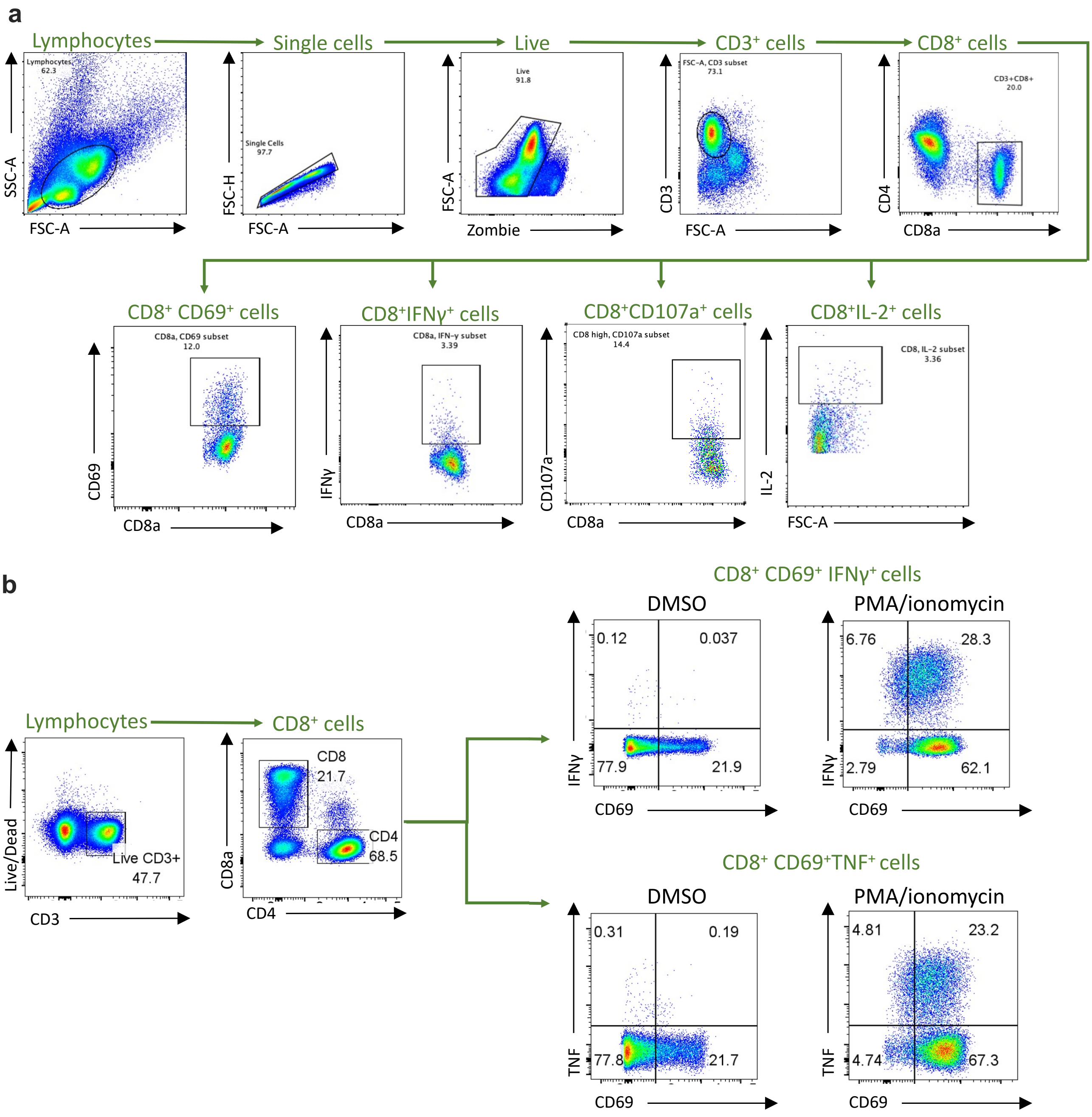

SI. 10.1 | Flow cytometry gating strategy for human samples. a, Gating strategy for PBMCs from *Pv*-infected patients from Brazil. Lymphocytes were first identified based on forward and side scatter properties (SSC-A vs FSC-A), followed by exclusion of doublets (FSC-H vs FSC-A), and viability dye (Zombie^{+}). Live single lymphocytes were then gated on CD3^{+} and CD8^{+} T cells, and the frequencies of CD69^{+} cells, CD107a^{+} cells, and cytokine-producing cells (IFNγ and IL-2) were verified. **b,** Gating strategy for PBMCs from CHMI volunteers. Live single cells were gated on CD3^{+} and CD8^{+}, and activation (CD69) and cytokine production (IFNγ or TNFα) were assessed.

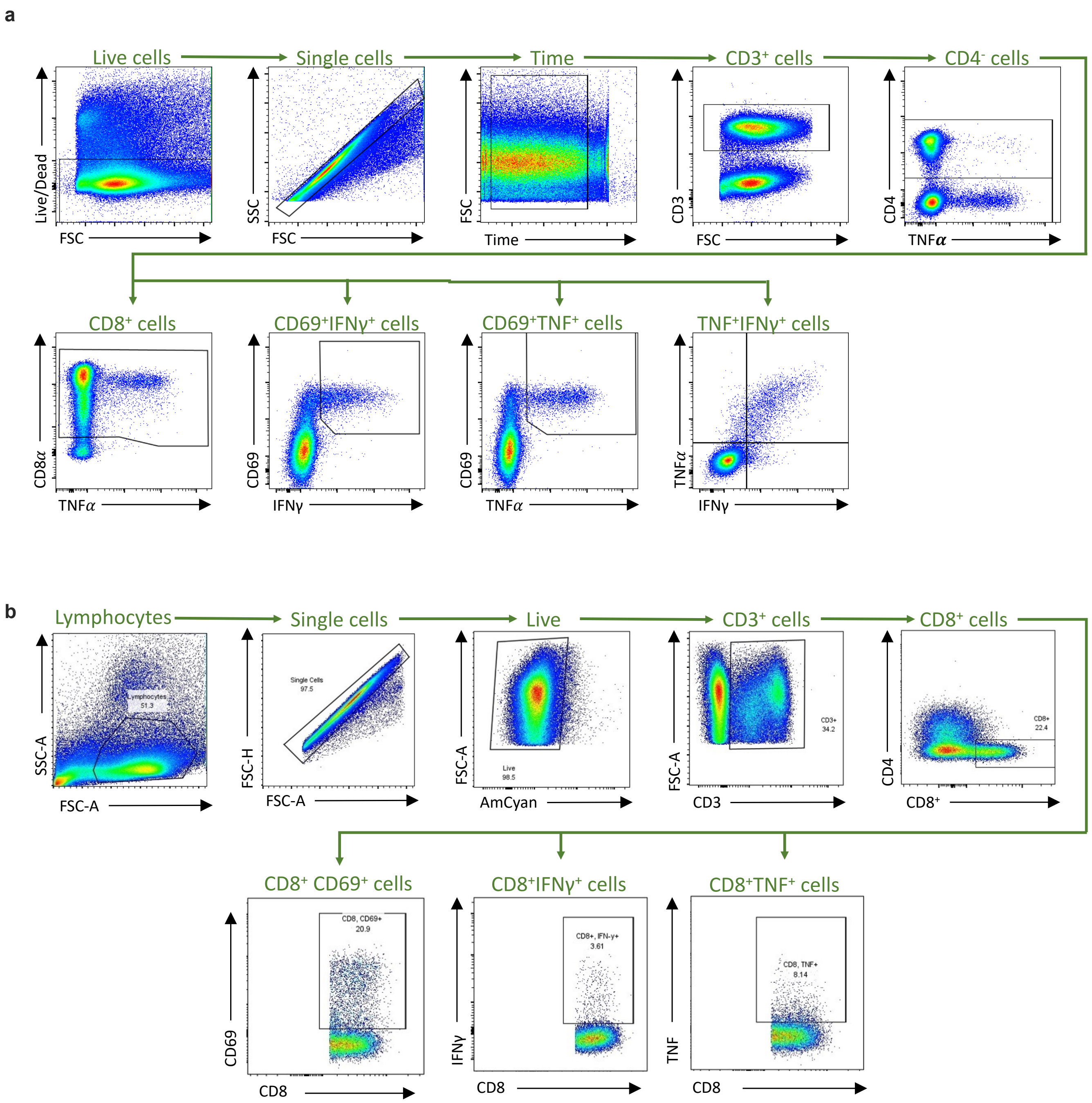

SI. 10.2 | Flow cytometry gating strategy for NHP and mice samples. a, Gating strategy for PBMC of NHPs. Live Lymphocytes were first identified (Live/Dead vs FSC), followed by exclusion of doublets (FSC-H vs FSC-A), and removal of acquisition artifacts using time gating. Live singlet lymphocytes were then gated on $CD3^+$ and $CD8^+$ T cells, and the frequencies of $CD69^+$ cells and cytokine-producing cells (IFNγ and/or TNFα) were verified. **b,** Gating strategy for mice splenocytes. Lymphocytes were first identified based on forward and side scatter properties (SSC-A vs FSC-A), followed by exclusion of doublets and dead cells ($Zombie^+$). Live single lymphocytes were then gated on $CD3^+$ and $CD8^+$ T cells, and the frequencies of $CD69^+$ cells and IFNγ or TNFα-producing cells were assessed.
