# Supplementary material for: Identification of cross-stage, cross-species malaria CD8+ T cell antigens
Source: Nature. 2026 Jul 1;655(8123):747–57. doi: 10.1038/s41586-026-10730-1 (PMC13661247; doi:10.1038/s41586-026-10730-1)
Supplement: Supplementary file 4 [file 41586_2026_10730_MOESM1_ESM.zip › 2025-05-11433B-s1/Supplementary_Information_6.pdf]

Supplementary information 6 | Spectral match validation

Peptide			Biological peptide				Synthetic peptide				Validation
Sequence	ID	n	[m/z]	z	RT	Peaks Score	[m/z]	z	RT	Peaks Score	(y/n)
AIEPSLAQLAQK	Ubqt/ribos-1	12	635.3643	2	41.42	37.49	634.8646	2	41.64	48.35	Y
AKAVKKQKKTLKPV	60S L31	14	523.0190	3	21.67	45.30	NA	NA	NA	NA	NA
ANKALLPTAGDD	60S L4/L1-1	12	593.3108	2	34.53	50.03	593.3093	2	34.31	52.84	Y
APISAGIKKPHR	Hist. H3	12	425.5951	3	25.01	40.59	425.5946	3	23.20	23.34	Y
AQKIKKKKKLTPA	Don Juan	13	494.6678	3	24.09	36.08	494.6680	3	20.52	41.55	Y
ASGGVLPNIHNV	Hist. H2A-2	12	589.3206	2	39.78	25.89	589.3203	2	39.89	37.05	Y
AVKKVGKIVK	60S L32-2	10	357.2537	3	22.97	22.03	357.2534	3	20.62	27.81	Y
DNNEHVVQEKTV	Unch A5JZN7.2	12	706.3463	2	27.81	53.74	706.3451	2	26.94	49.45	Y
DNNEHVVQEKTVSF	Unch A5JZN7.1	14	549.2657	3	35.20	61.62	549.2657	3	34.69	47.01	Y
EDYSPRKV	Unch A5K8G9	8	497.2546	2	26.23	39.03	497.2538	2	24.87	29.67	Y
GAFGKPNGV	60S L10	9	423.7279	2	30.37	19.14	423.7272	2	29.41	29.60	Y
GKGKNKEKL	40S S25-1	9	501.3087	2	20.20	33.76	501.3089	2	19.19	25.14	Y
GKGKNKEKLNHAVF	40S S25-2	14	523.9681	3	25.17	48.81	523.9667	3	24.09	25.39	Y
GLNQKQPTKGSNIQ	ETRAMP-2	14	757.4133	2	24.53	37.29	756.9106	2	23.51	45.90	Y
GPKRATKIRK	40S S6-3	10	385.5873	3	20.09	38.30	385.5876	3	18.95	33.14	Y
GRIGRYLKKGKYA	Hist. H2A-4	13	378.2308	4	32.42	33.32	378.2307	4	26.84	33.94	Y
GRIGRYLKKGKYAK	Hist. H2A-1	14	410.2536	4	30.86	33.56	410.2541	4	25.27	17.95	Y
GSKQVHVV	40S S8-2	8	427.2482	2	24.78	41.76	427.2479	2	23.31	37.69	Y
GTGRKKGPNSKL	40S S30-2	12	414.9137	3	20.78	46.04	414.9140	3	19.77	36.32	Y
GTPIEKLHPI	60S L13-2	10	552.8251	2	34.59	27.43	552.8255	2	34.19	35.45	Y
GVKKDVAK	40S S6-2	8	422.7661	2	19.95	34.31	422.7660	2	18.91	26.58	Y
KKVAAGYKKLTD	ETRAMP-1	12	441.2663	3	24.33	41.58	441.2662	3	22.62	39.46	Y
KKYKNKKFKPY	60S L35	11	491.2969	3	27.30	25.41	491.6320	3	23.68	19.14	Y
KNIKSKNGIGGIPAD	60S L13-3	15	504.6231	3	27.71	31.77	504.6229	3	26.84	16.62	Y
KSAGADSKSLKKLD	ETRAMP-5	14	483.6093	3	23.95	34.26	483.2758	3	22.52	44.23	Y
KVPVPPTQAKKPKKN	Hist. H2A-5	15	415.7615	4	23.19	35.10	415.7620	4	21.87	40.76	Y
LETYQNMKIQKQTP	40S S2	14	861.4510	2	32.68	40.56	861.9502	2	31.64	48.98	Y
LGGLNQKQPT	ETRAMP-4	10	528.2960	2	28.65	37.38	528.2964	2	27.59	39.32	Y
LILRAAIKTK	Unch A5K8J8	10	1126.7635	1	54.45	17.89	376.2605	3	29.11	20.42	N
LNQKQPTKGSNIQ	ETRAMP-3	13	728.4005	2	23.52	25.98	728.4000	2	22.49	41.12	Y
MYKKVYVID	60S 13a	9	579.8156	2	32.90	39.80	579.8154	2	31.92	31.61	Y
NKALLPTAGDD	60S L4/L1-2	11	557.7906	2	34.65	43.43	557.7903	2	35.38	50.46	Y
NQLRPKKKLK	Ubqt/ribos-2	10	418.2789	3	21.63	26.12	418.2785	3	19.94	19.81	Y
PKFFKNQRY	60S L29	9	409.8929	3	28.72	38.08	409.8927	3	27.66	31.37	Y
RLTGGKKKIHKKK	40S S8-1	13	381.5072	4	20.20	43.53	NA	NA	NA	NA	NA
SDGTGRKKGPNSKL	40S S30-1	14	482.2673	3	21.44	48.01	482.2670	3	20.18	37.03	Y
SFFNSKKIKKGSKS	40S S11	14	529.3069	3	25.90	41.15	397.2319	4	23.01	37.69	Y
SGGVLPNIHNV	Hist. H2A-3	11	553.8022	2	39.63	29.28	533.8016	2	39.69	34.16	Y
SKNGKNRFIKPKIQ	40S S6-1	14	415.2520	4	26.02	36.88	415.2518	4	24.19	30.73	Y
SSHAKGIVVEKV	40S S23	12	418.5784	3	27.41	44.55	418.5784	3	25.94	43.89	Y
TGRKKGPNSKL	40S S30-3	11	395.9069	3	20.74	37.56	297.1819	4	19.67	25.63	N
TPIITNKPFG	ETRAMP-6	10	544.3114	2	38.69	36.66	544.3113	2	38.87	46.35	Y
VITDVGDSDIIKTNE	60S L30	15	809.9135	2	41.55	55.90	809.9135	2	41.11	55.13	Y
VSEVTTVEKDE	60S L9	11	618.3045	2	30.31	35.50	618.3038	2	29.67	41.63	Y
YESIEVSKID	60S L13-1	10	591.7995	2	38.03	40.35	591.7988	2	37.62	41.05	Y
YGRIFKKKITKK	60S L4/L1-3	12	378.4963	4	28.58	35.21	378.2462	4	23.62	15.05	N

n amino acid length
[m/z] Mass over Charge
z charge state
RT Retention Time
NA Not Available

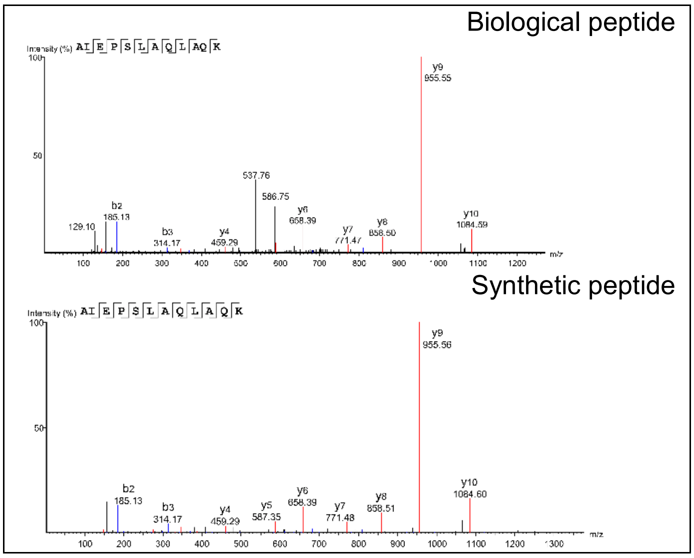
Biological peptide
Intensity (%) AIEPSLAQLAQK
y9 955.55
537.76
586.75
b2 185.13
129.10
b3 314.17
y4 459.29
y6 658.39
y7 771.47
y8 858.50
y10 1084.59
Synthetic peptide
Intensity (%) AIEPSLAQLAQK
y9 955.56
b2 185.13
b3 314.17
y4 459.29
y5 587.35
y6 658.39
y7 771.48
y8 858.51
y10 1084.60
m/z

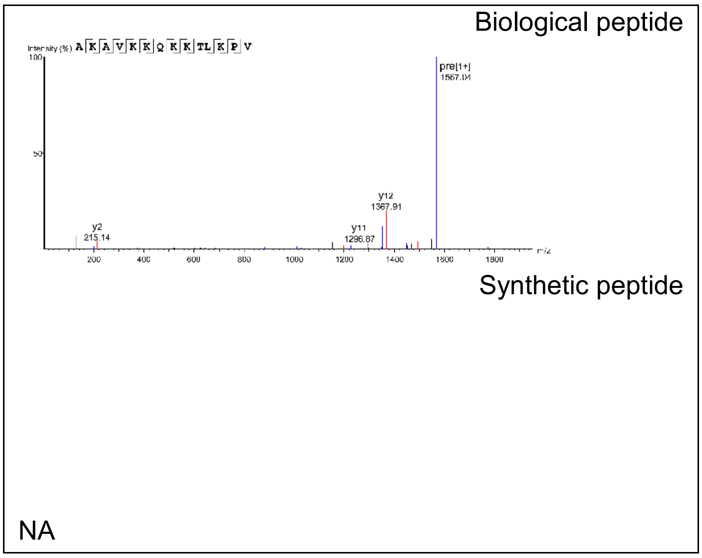
Biological peptide
Intensity (%) AKAVKKQKKTLKPV
pre[1+] 1567.04
y12 1367.91
y11 1296.87
y2 215.14
Synthetic peptide
NA

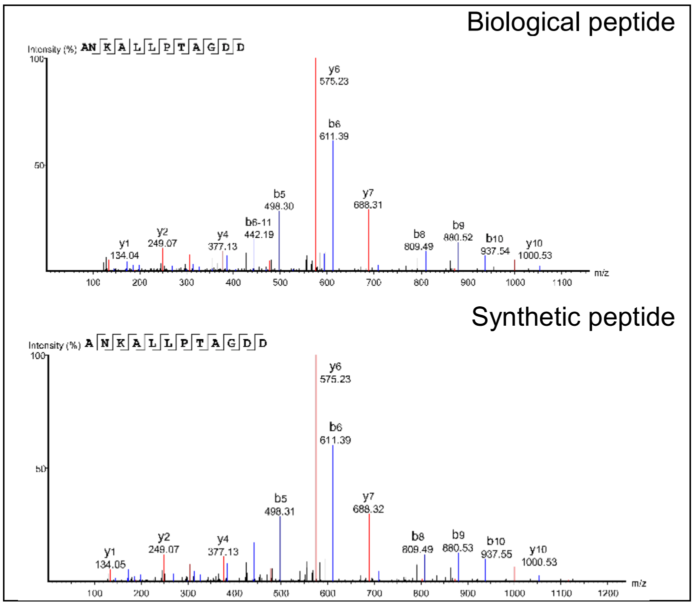
Biological peptide
Intensity (%) ANKALLPTAGDD
y6 575.23
b6 611.39
b5 498.30
y7 688.31
b6-11 442.19
y2 249.07
y4 377.13
y1 134.04
b8 809.49
b9 880.52
b10 937.54
y10 1000.53
m/z
Synthetic peptide
Intensity (%) ANKALLPTAGDD
y6 575.23
b6 611.39
b5 498.31
y7 688.32
y2 249.07
y4 377.13
y1 134.05
b8 809.49
b9 880.53
b10 937.55
y10 1000.53
m/z

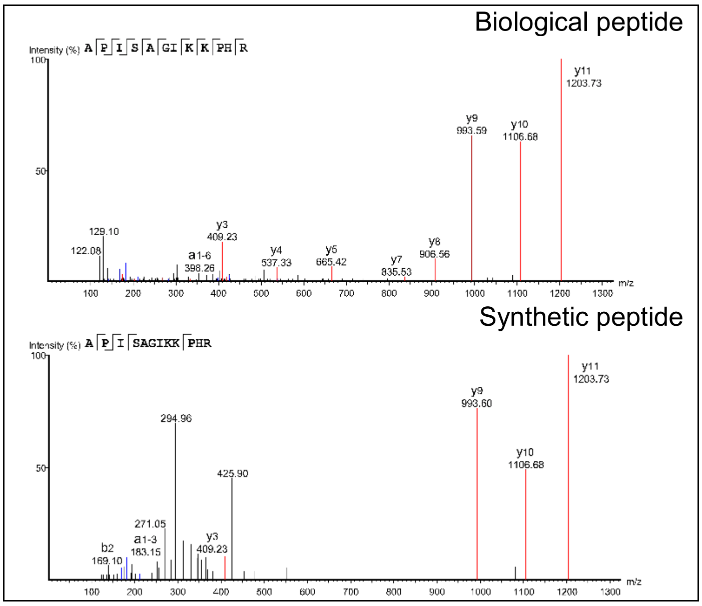
Biological peptide
Intensity (%) APISAGIKKPHR
y11 1203.73
y9 993.59
y10 1106.68
129.10
122.08
y3 409.23
a1-6 398.26
y4 537.33
y5 665.42
y7 835.53
y8 906.56
m/z
Synthetic peptide
Intensity (%) APISAGIKKPHR
y11 1203.73
y9 993.60
y10 1106.68
294.96
425.90
271.05
a1-3 183.15
b2 169.10
y3 409.23
m/z

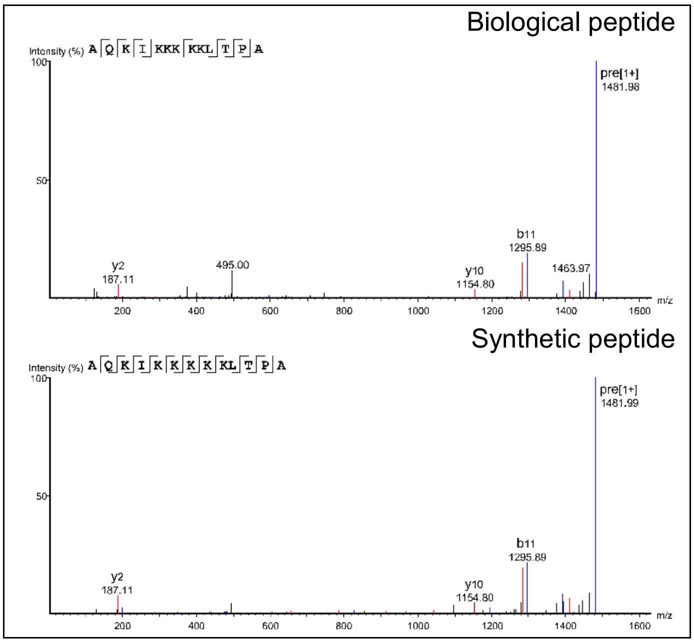
Biological peptide
Intensity (%) AQKIKKKKKLTPA
pre[1+] 1481.98
b11 1295.89
1463.97
y10 1154.80
495.00
y2 187.11
m/z
Synthetic peptide
Intensity (%) AQKIKKKKKLTPA
pre[1+] 1481.99
b11 1295.89
y10 1154.80
y2 187.11
m/z

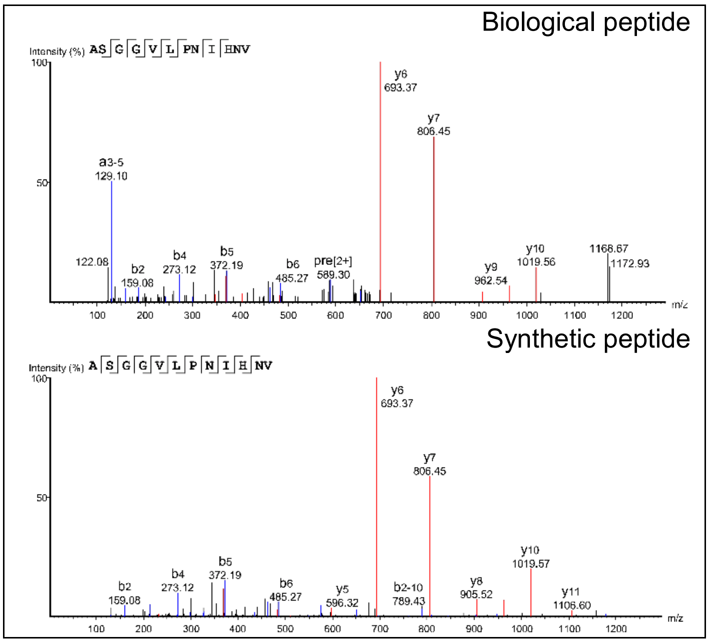
Biological peptide
Intensity (%) ASGGVLPNIHNV
y6 693.37
y7 806.45
a3-5 129.10
122.08
b2 159.08
b4 273.12
b5 372.19
b6 485.27
pre[2+] 589.30
y9 962.54
y10 1019.56
1168.67
1172.93
m/z
Synthetic peptide
Intensity (%) ASGGVLPNIHNV
y6 693.37
y7 806.45
b2 159.08
b4 273.12
b5 372.19
b6 485.27
y5 596.32
b2-10 789.43
y8 905.52
y10 1019.57
y11 1106.60
m/z

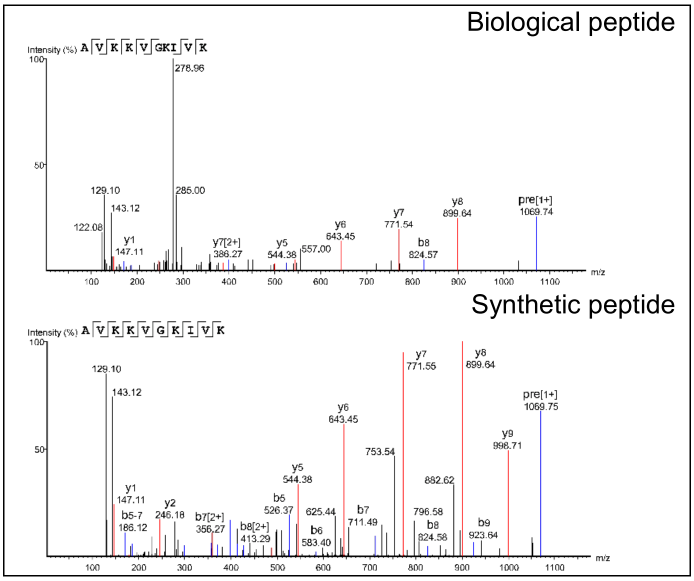
Biological peptide
Intensity (%) AVKKVGKIVK
pre[1+] 1069.74
y8 899.64
y7 771.54
y6 643.45
278.96
285.00
129.10
143.12
Synthetic peptide
Intensity (%) AVKKVGKIVK
pre[1+] 1069.75
y8 899.64
y7 771.55
y9 998.71
y6 643.45
129.10
143.12
m/z

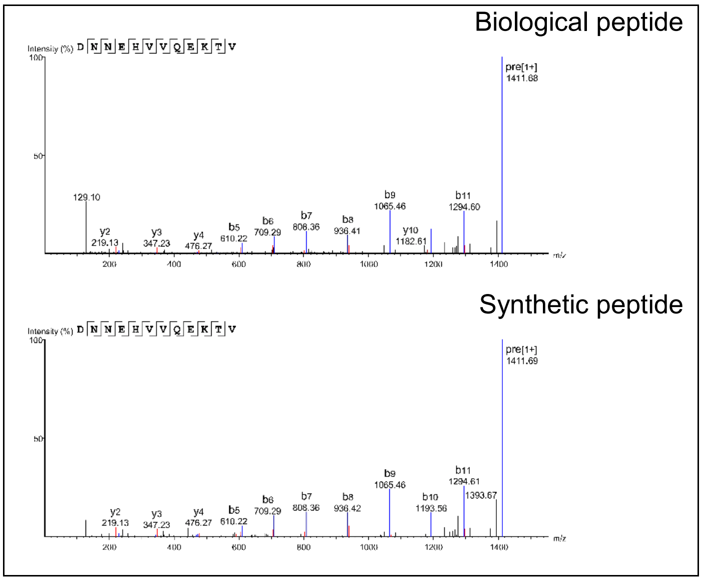
Biological peptide
Intensity (%) DNNEHVVQEKTV
pre[1+] 1411.68
b9 1065.46
b11 1294.60
129.10
Synthetic peptide
Intensity (%) DNNEHVVQEKTV
pre[1+] 1411.69
b9 1065.46
b11 1294.61
1393.67
m/z

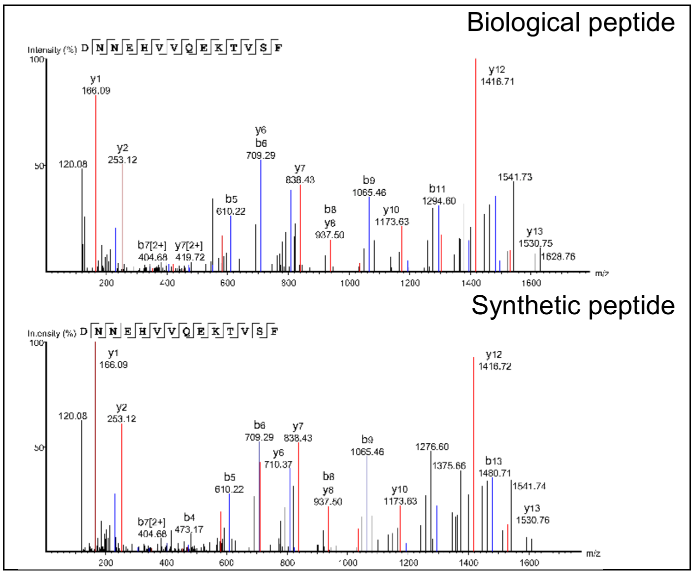
Biological peptide
Intensity (%) DNNEHVVQEKTVSF
y12 1416.71
y1 166.09
y2 253.12
y6 b6 709.29
y7 838.43
1541.73
Synthetic peptide
Intensity (%) DNNEHVVQEKTVSF
y12 1416.72
y1 166.09
y2 253.12
b6 709.29
y7 838.43
m/z

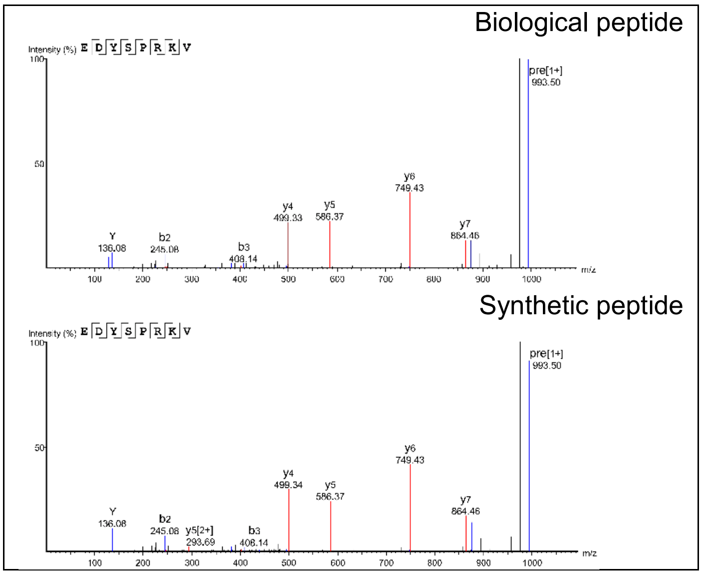
Biological peptide
Intensity (%) EDYSPRKV
pre[1+] 993.50
y6 749.43
y4 499.33
y5 586.37
y7 864.46
Synthetic peptide
Intensity (%) EDYSPRKV
pre[1+] 993.50
y6 749.43
y4 499.34
y5 586.37
y7 864.46
m/z

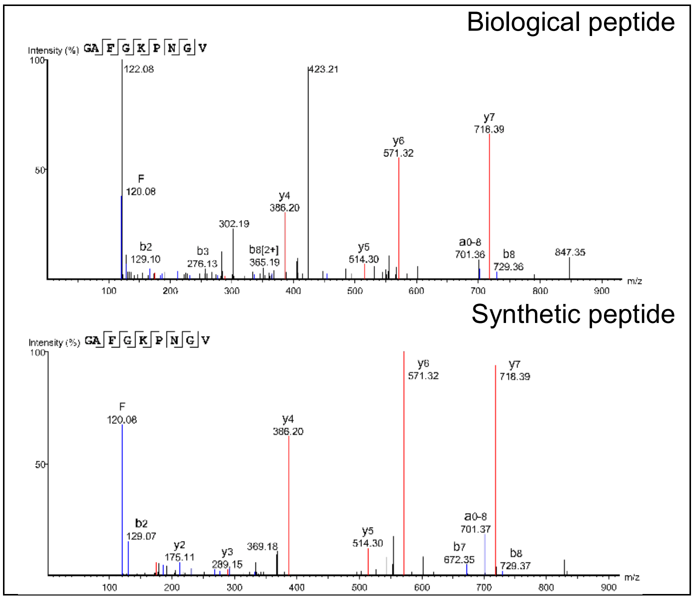
Biological peptide
Intensity (%) GAFGKPNGV
122.08
423.21
y7 718.39
y6 571.32
F 120.08
y4 386.20
Synthetic peptide
Intensity (%) GAFGKPNGV
y6 571.32
y7 718.39
F 120.08
y4 386.20
m/z

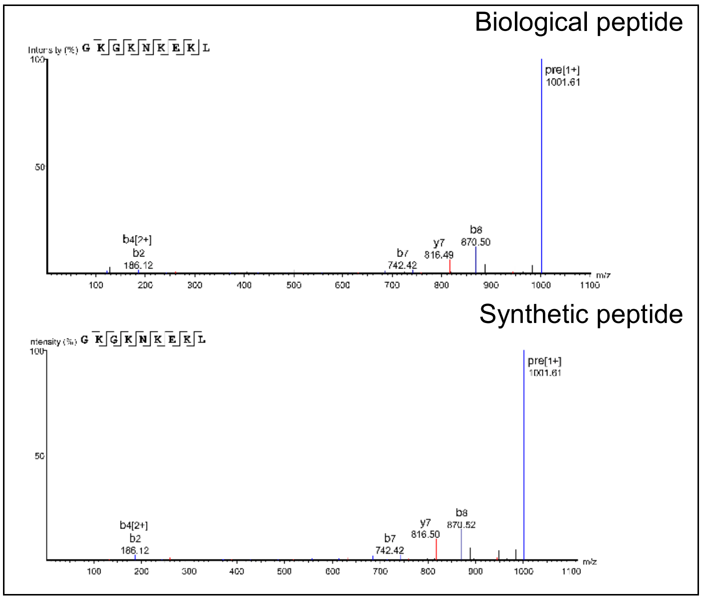
Biological peptide
Intensity (%) GKGKNKEKL
pre[1+] 1001.61
b8 870.50
y7 816.49
b7 742.42
Synthetic peptide
Intensity (%) GKGKNKEKL
pre[1+] 1001.61
b8 870.52
y7 816.50
b7 742.42
m/z

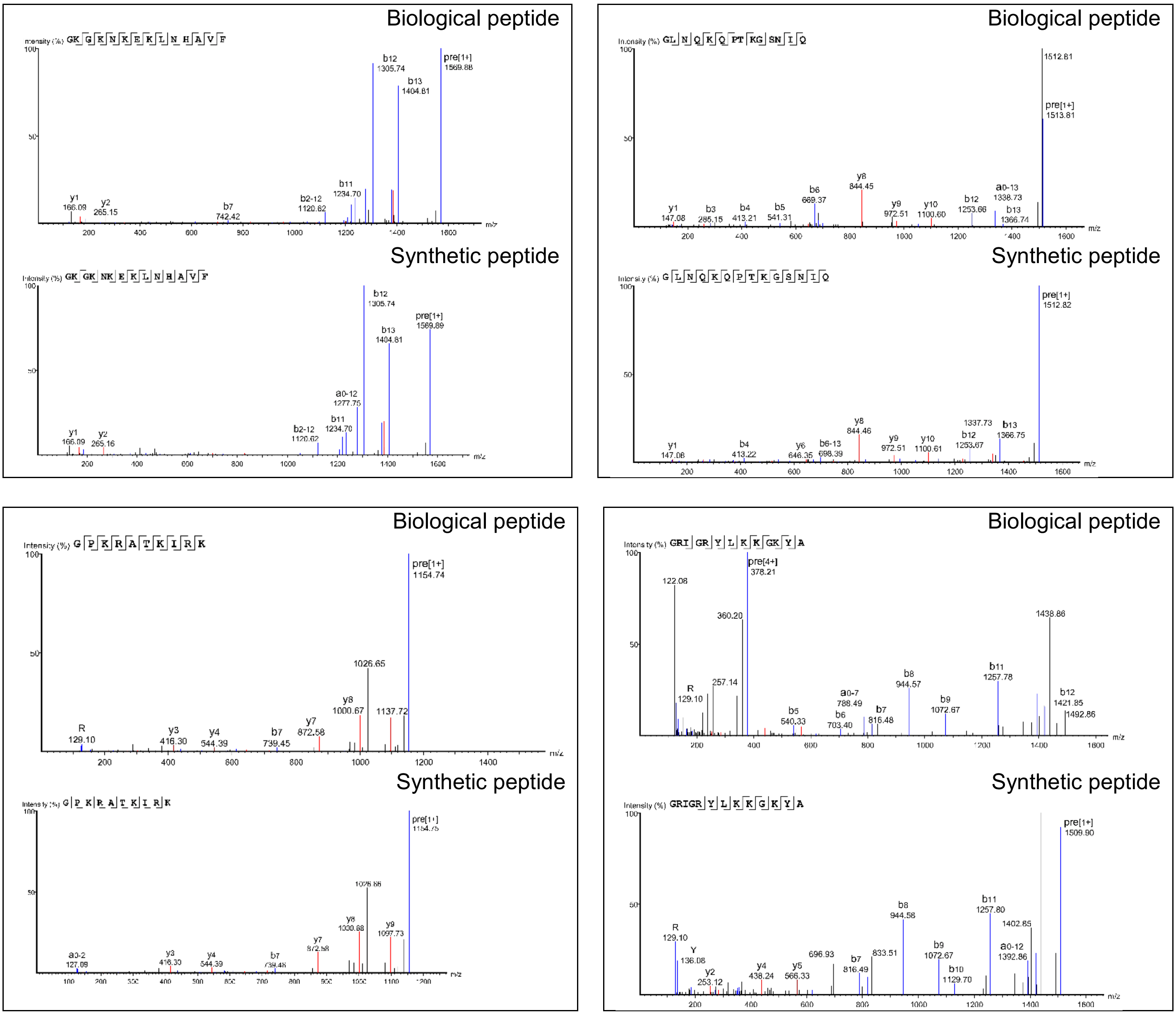

Biological peptide
Intensity (%) GKGKNKEKLNHAVF
b12 1305.74
pre[1+] 1569.88
b13 1404.81
b11 1234.70
b2-12 1120.62
b7 742.42
y1 166.09
y2 265.15
m/z
Synthetic peptide
pre[1+] 1569.89
a0-12 1277.75
b2-12 1120.62
y2 265.16
Biological peptide
Intensity (%) GLNQKQPTKGSNIQ
1512.81
pre[1+] 1513.81
y8 844.45
b6 669.37
a0-13 1338.73
b12 1253.66
b13 1366.74
y9 972.51
y10 1100.60
b5 541.31
b4 413.21
b3 285.15
y1 147.08
Synthetic peptide
pre[1+] 1512.82
y8 844.46
1337.73
b13 1366.75
b12 1253.67
y9 972.51
y10 1100.61
b6-13 698.39
y6 646.35
b4 413.22
Biological peptide
Intensity (%) GPKRATKIRK
pre[1+] 1154.74
1026.65
y8 1000.67
1137.72
y7 872.58
b7 739.45
y4 544.39
y3 416.30
R 129.10
Synthetic peptide
pre[1+] 1154.75
1026.68
y8 1000.68
y9 1097.73
y7 872.58
b7 739.48
y4 544.39
y3 416.30
a0-2 127.09
Biological peptide
Intensity (%) GRIGRYLKKGKYA
pre[4+] 378.21
122.08
360.20
1438.86
b11 1257.78
b8 944.57
a0-7 788.49
b12 1421.85
1492.86
b9 1072.67
b7 816.48
b6 703.40
b5 540.33
257.14
R 129.10
Synthetic peptide
pre[1+] 1509.90
b11 1257.80
b8 944.58
1402.85
a0-12 1392.86
b9 1072.67
b10 1129.70
833.51
b7 816.49
696.93
y5 566.33
y4 438.24
y2 253.12
Y 136.08
R 129.10

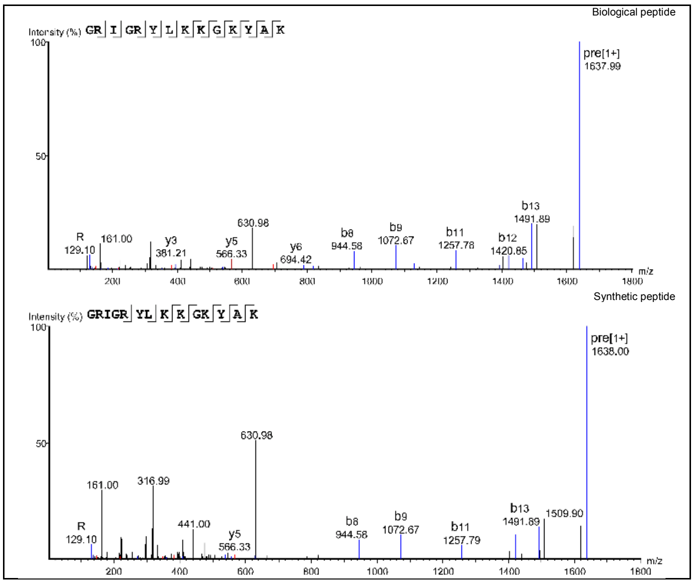
Biological peptide
Intensity (%) GR I GR Y L K K G K Y A K
pre[1+] 1637.99
R 129.10
161.00
y3 381.21
630.98
y5 566.33
y6 694.42
b8 944.58
b9 1072.67
b11 1257.78
b12 1420.85
b13 1491.89
m/z
Synthetic peptide
Intensity (%) GRIGR YL K K GK Y A K
pre[1+] 1638.00
630.98
161.00
316.99
R 129.10
441.00
y5 566.33
b8 944.58
b9 1072.67
b11 1257.79
b13 1491.89
1509.90
m/z

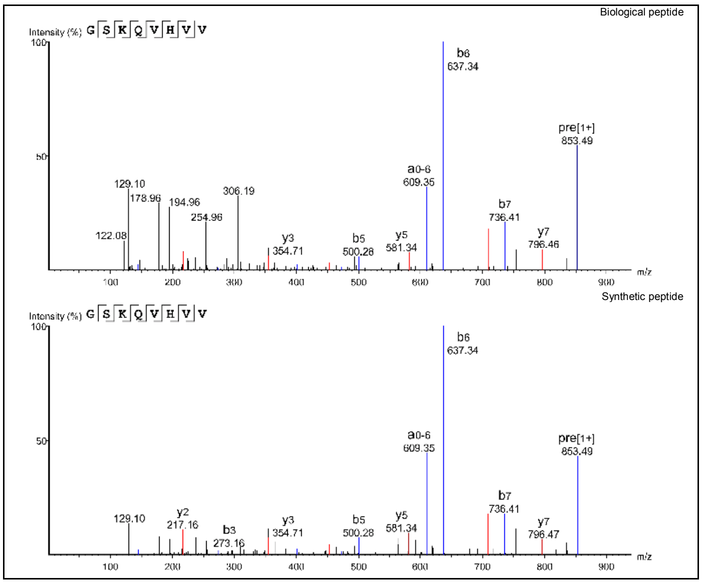
Biological peptide
Intensity (%) G S K Q V H V V
b6 637.34
pre[1+] 853.49
a0-6 609.35
129.10
178.96
194.96
306.19
254.96
122.08
y3 354.71
b5 500.28
y5 581.34
b7 736.41
y7 796.46
m/z
Synthetic peptide
Intensity (%) G S K Q V H V V
b6 637.34
a0-6 609.35
pre[1+] 853.49
b7 736.41
129.10
y2 217.16
b3 273.16
y3 354.71
b5 500.28
y5 581.34
y7 796.47
m/z

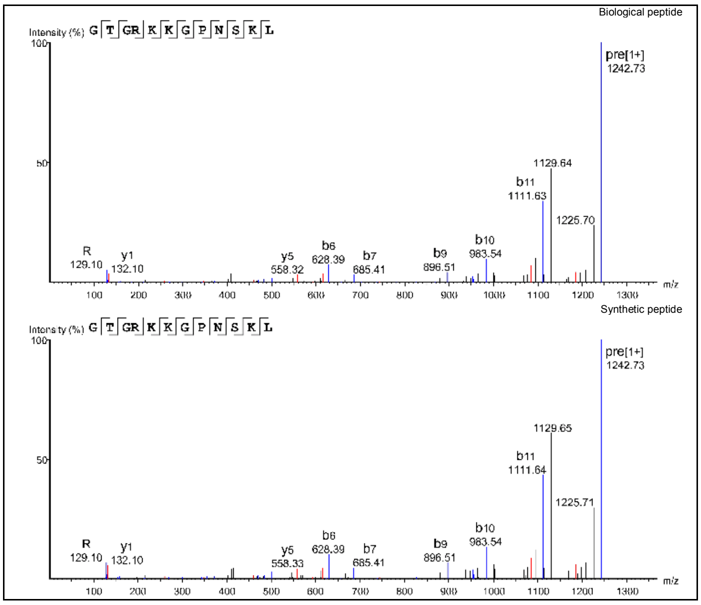
Biological peptide
Intensity (%) G T GR K K G P N S K L
pre[1+] 1242.73
1129.64
b11 1111.63
1225.70
R 129.10
y1 132.10
y5 558.32
b6 628.39
b7 685.41
b9 896.51
b10 983.54
m/z
Synthetic peptide
Intensity (%) G T GR K K G P N S K L
pre[1+] 1242.73
1129.65
b11 1111.64
1225.71
R 129.10
y1 132.10
y5 558.33
b6 628.39
b7 685.41
b9 896.51
b10 983.54
m/z

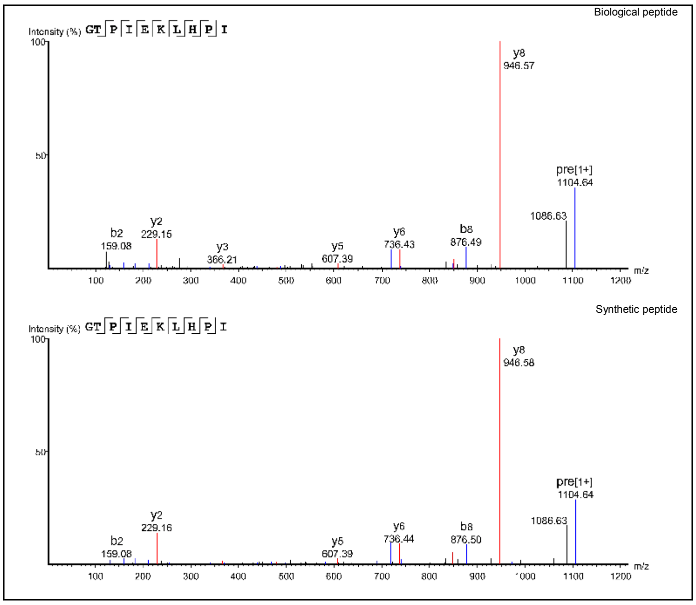
Biological peptide
Intensity (%) GT P I E K L H P I
y8 946.57
pre[1+] 1104.64
1086.63
b2 159.08
y2 229.15
y3 366.21
y5 607.39
y6 736.43
b8 876.49
m/z
Synthetic peptide
Intensity (%) GT P I E K L H P I
y8 946.58
pre[1+] 1104.64
1086.63
y2 229.16
b2 159.08
y5 607.39
y6 736.44
b8 876.50
m/z

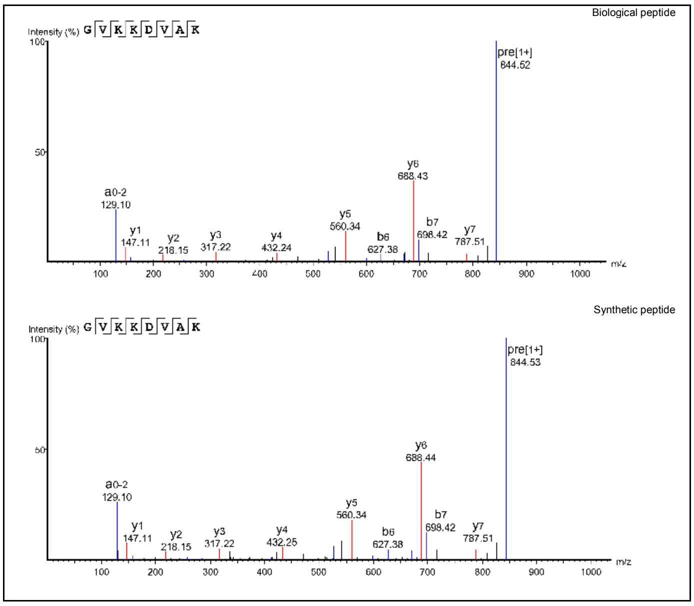
Biological peptide
Intensity (%) G V K K D V A K
pre[1+] 844.52
y6 688.43
a0-2 129.10
y1 147.11
y2 218.15
y3 317.22
y4 432.24
y5 560.34
b6 627.38
b7 698.42
y7 787.51
m/z
Synthetic peptide
Intensity (%) G V K K D V A K
pre[1+] 844.53
y6 688.44
a0-2 129.10
y1 147.11
y2 218.15
y3 317.22
y4 432.25
y5 560.34
b6 627.38
b7 698.42
y7 787.51
m/z

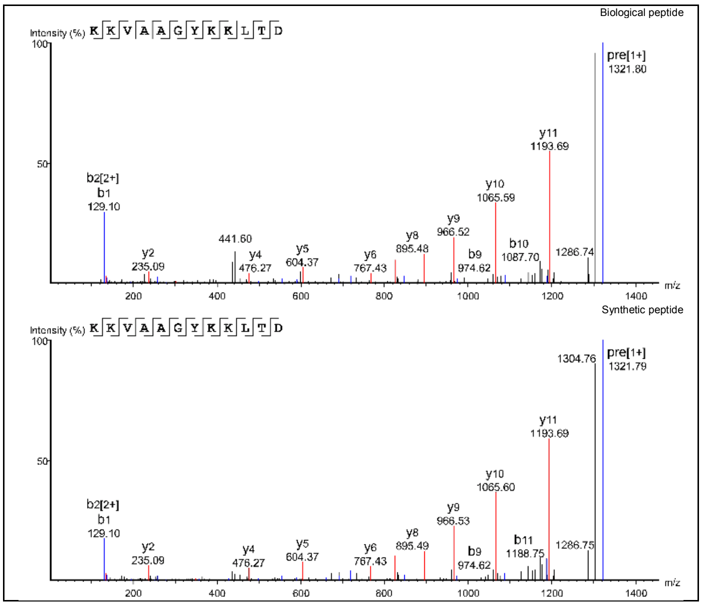
Biological peptide
Intensity (%) K K V A A G Y K K L T D
pre[1+] 1321.80
y11 1193.69
y10 1065.59
b2[2+]
b1
129.10
y9 966.52
y8 895.48
b10 1087.70
1286.74
441.60
y2 235.09
y4 476.27
y5 604.37
y6 767.43
b9 974.62
m/z
Synthetic peptide
Intensity (%) K K V A A G Y K K L T D
1304.76
pre[1+] 1321.79
y11 1193.69
y10 1065.60
y9 966.53
b2[2+]
b1
129.10
y2 235.09
y4 476.27
y5 604.37
y6 767.43
y8 895.49
b9 974.62
b11 1188.75
1286.75
m/z

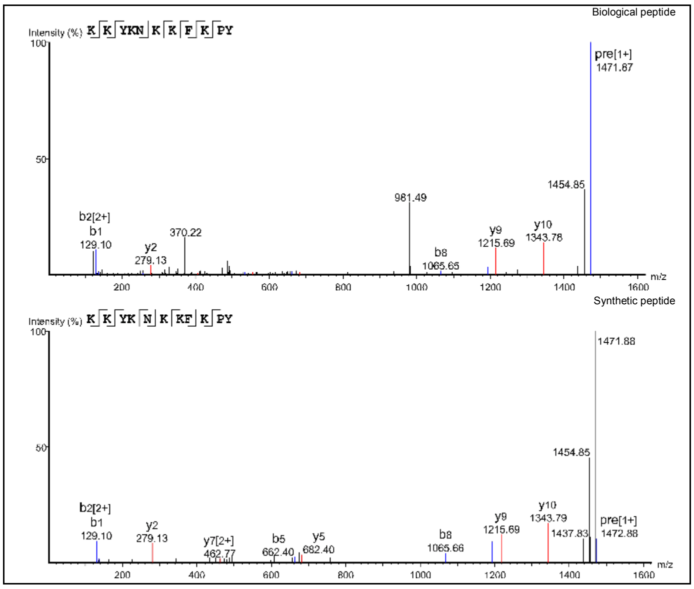
Biological peptide
Intensity (%) K K YKN K K F K PY
pre[1+] 1471.87
1454.85
981.49
b2[2+]
b1 129.10
370.22
y2 279.13
b8 1065.65
y9 1215.69
y10 1343.78
m/z
Synthetic peptide
Intensity (%) K K YK N K KF K PY
1471.88
1454.85
b2[2+]
b1 129.10
y2 279.13
y7[2+] 462.77
b5 662.40
y5 682.40
b8 1065.66
y9 1215.69
y10 1343.79
1437.83
pre[1+] 1472.88
m/z

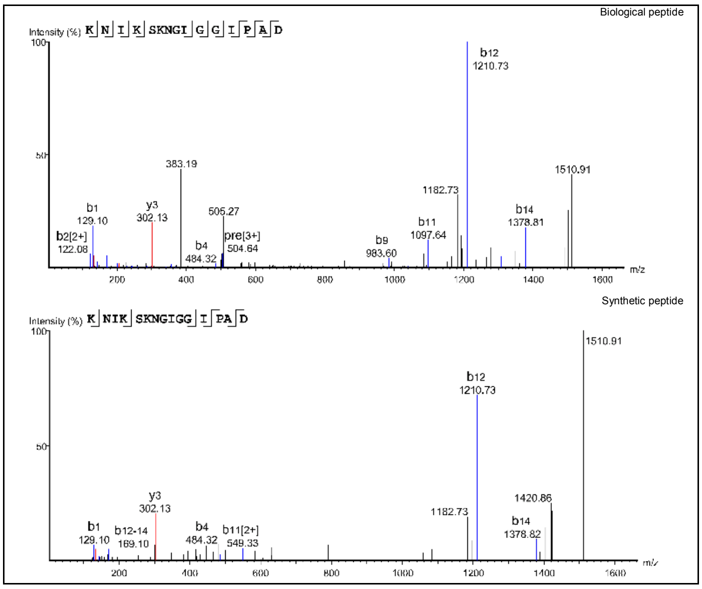
Biological peptide
Intensity (%) K N I K SKNGI G G I P A D
b12 1210.73
383.19
1510.91
1182.73
b1 129.10
y3 302.13
505.27
b2[2+] 122.08
b4 484.32
pre[3+] 504.64
b9 983.60
b11 1097.64
b14 1378.81
m/z
Synthetic peptide
Intensity (%) K NIK SKNGIGG I PA D
1510.91
b12 1210.73
y3 302.13
1420.86
1182.73
b1 129.10
b12-14 169.10
b4 484.32
b11[2+] 549.33
b14 1378.82
m/z

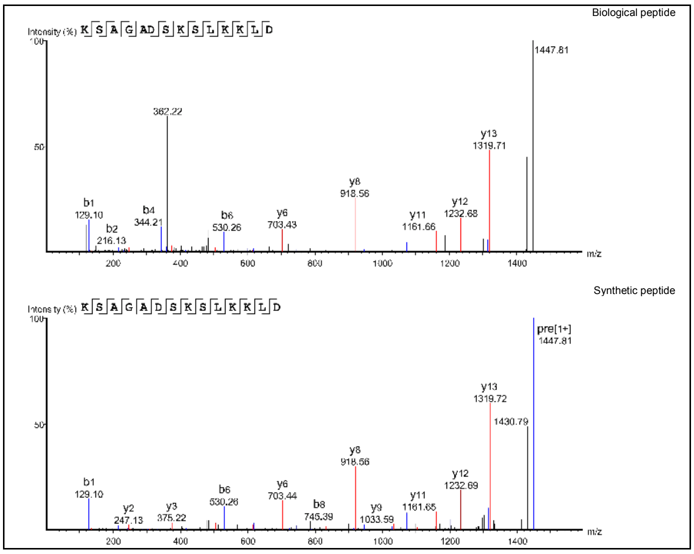
Biological peptide
Intensity (%) K S A G AD S K S L K K L D
1447.81
362.22
y13 1319.71
y8 918.56
b1 129.10
b4 344.21
b2 216.13
b6 530.26
y6 703.43
y11 1161.66
y12 1232.68
m/z
Synthetic peptide
Intensity (%) K S A G A D S K S L K K L D
pre[1+] 1447.81
y13 1319.72
1430.79
y8 918.56
b1 129.10
y2 247.13
y3 375.22
b6 530.26
y6 703.44
b8 745.39
y9 1033.59
y11 1161.65
y12 1232.69
m/z

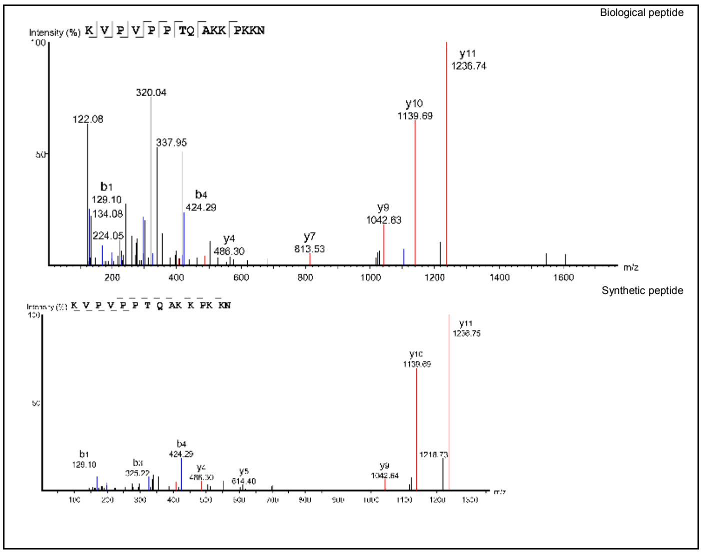
Biological peptide
Intensity (%) K V P V P P TQ AKK PKKN
y11 1236.74
320.04
y10 1139.69
122.08
337.95
b1 129.10
134.08
224.05
b4 424.29
y9 1042.63
y4 486.30
y7 813.53
m/z
Synthetic peptide
y11 1236.75
y10 1139.69
b4 424.29
1218.73
m/z

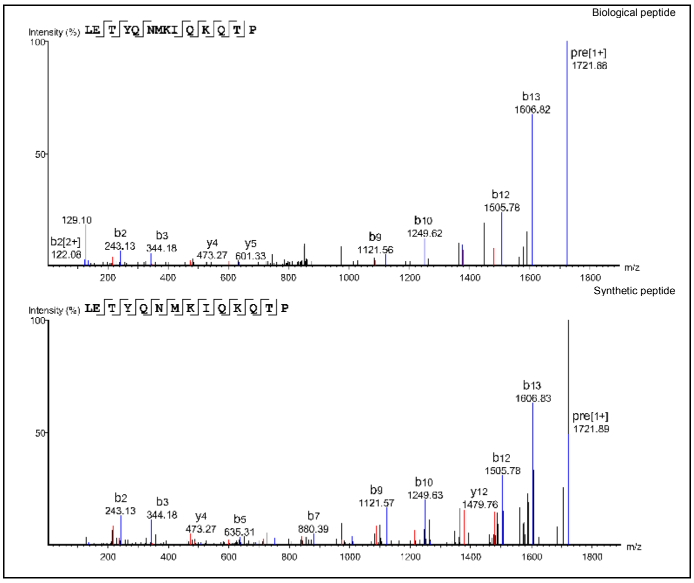
Biological peptide
Intensity (%) LE T YQ NMKI Q K Q T P
pre[1+] 1721.88
b13 1606.82
b12 1505.78
129.10
b10 1249.62
b2[2+] 122.08
b2 243.13
b3 344.18
y4 473.27
y5 601.33
b9 1121.56
m/z
Synthetic peptide
Intensity (%) LE T Y Q N M K I Q K Q T P
b13 1606.83
pre[1+] 1721.89
b12 1505.78
b2 243.13
b3 344.18
y4 473.27
b5 635.31
b7 880.39
b9 1121.57
b10 1249.63
y12 1479.76
m/z

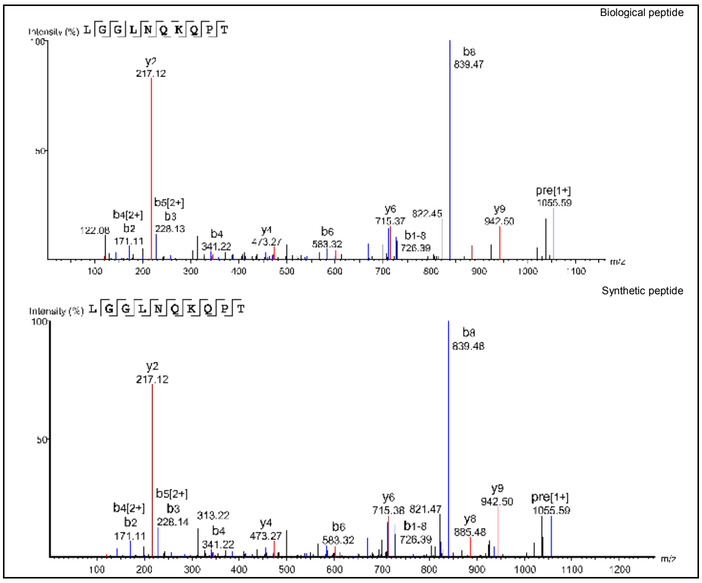
Biological peptide
Intensity (%) L G G L N Q K Q P T
y2 217.12
b8 839.47
pre[1+] 1055.59
b5[2+]
b4[2+]
b2 171.11
122.08
b3 228.13
b4 341.22
y4 473.27
b6 583.32
y6 715.37
822.45
b1-8 726.39
y9 942.50
m/z
Synthetic peptide
Intensity (%) L G G L N Q K Q P T
b8 839.48
y2 217.12
b5[2+]
b4[2+]
b2 171.11
b3 228.14
313.22
b4 341.22
y4 473.27
b6 583.32
y6 715.38
821.47
b1-8 726.39
y8 885.48
y9 942.50
pre[1+] 1055.59
m/z

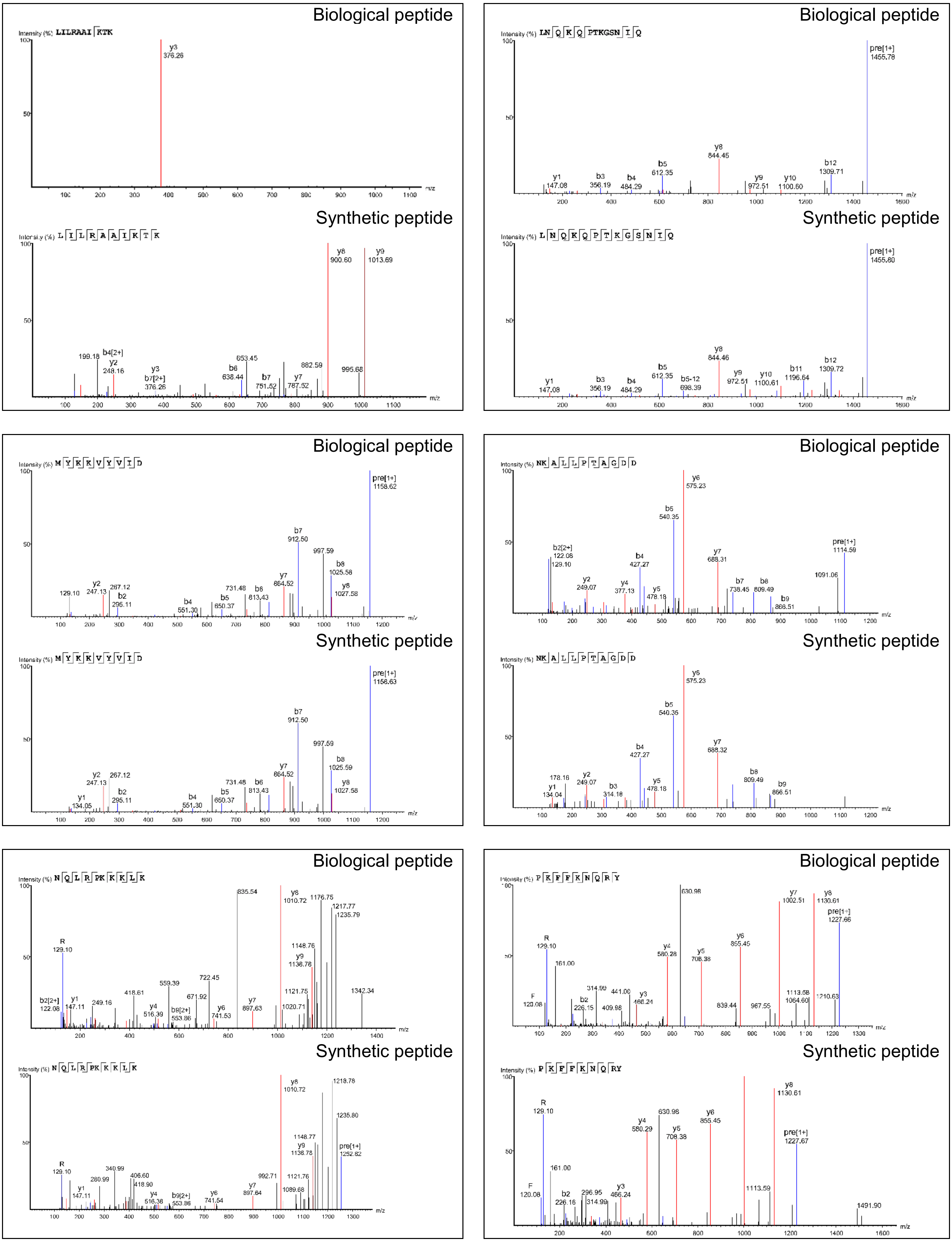
Biological peptide
Intensity (%) LILRAAI KTK
Synthetic peptide
Intensity (%) L I L R A A I K T K
Biological peptide
Intensity (%) LN Q K Q PTKGSN I Q
Synthetic peptide
Intensity (%) L N Q K Q P T K G S N I Q
Biological peptide
Intensity (%) M Y K K V Y V I D
Synthetic peptide
Intensity (%) M Y K K V Y V I D
Biological peptide
Intensity (%) NK A L L P T A G D D
Synthetic peptide
Intensity (%) NK A L L P T A G D D
Biological peptide
Intensity (%) N Q L R PK K K L K
Synthetic peptide
Intensity (%) N Q L R PK K K L K
Biological peptide
Intensity (%) P K F F K N Q R Y
Synthetic peptide
Intensity (%) P K F F K N Q RY
m/z

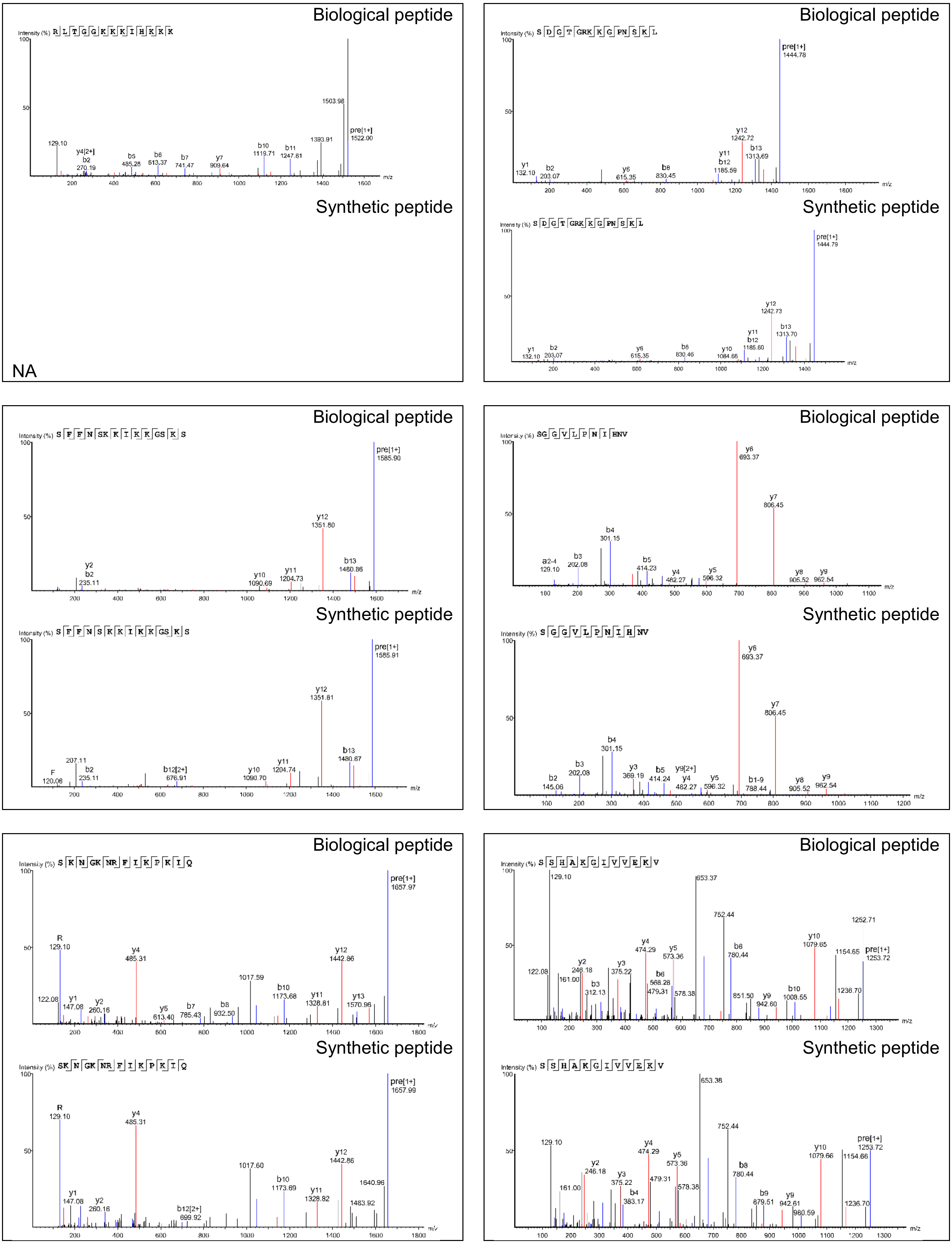
Biological peptide
R L T G G K K K I H K K K
pre[1+] 1522.00
1503.98
1393.91
Synthetic peptide
NA
Biological peptide
S D G T GRK K G PN S K L
pre[1+] 1444.78
Synthetic peptide
S D G T GRK K G PN S K L
pre[1+] 1444.79
Biological peptide
S F F N SK K I K K GS K S
pre[1+] 1585.90
Synthetic peptide
S F F N S K K I K K GS K S
pre[1+] 1585.91
Biological peptide
SG G V L P N I HNV
Synthetic peptide
S G G V L P N I H NV
Biological peptide
S K N GK NR F I K P K I Q
pre[1+] 1657.97
Synthetic peptide
SK N GK NR F I K P K I Q
pre[1+] 1657.99
Biological peptide
S S H A K G I V V E K V
pre[1+] 1253.72
Synthetic peptide
S S H A K G I V V E K V
pre[1+] 1253.72
Intensity (%)
m/z

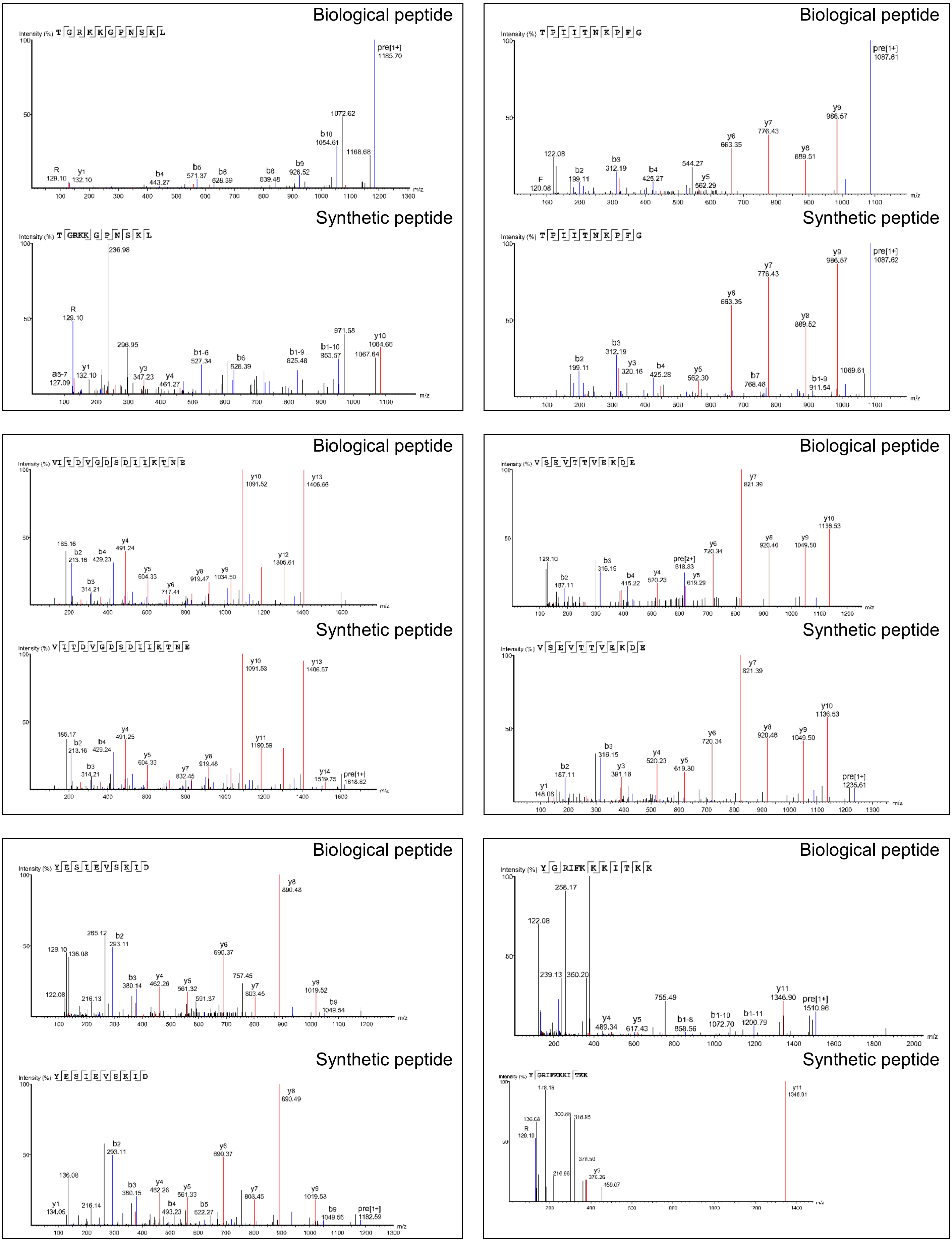
Biological peptide
T G R K K G P N S K L
Synthetic peptide
T GRKK G P N S K L
Biological peptide
T P I I T N K P F G
Synthetic peptide
T P I I T N K P F G
Biological peptide
V I T D V G D S D I I K T N E
Synthetic peptide
V I T D V G D S D I I K T N E
Biological peptide
V S E V T T V E K D E
Synthetic peptide
V S E V T T V E K D E
Biological peptide
Y E S I E V S K I D
Synthetic peptide
Y E S I E V S K I D
Biological peptide
Y G RIFK K K I T K K
Synthetic peptide
Y GRIFKKKI TKK
